# The Role of Wildfires in the Interplay of Forest Carbon Stocks and Wood Harvest in the Contiguous United States During the 20th Century

**DOI:** 10.1029/2023GB007813

**Published:** 2023-08-17

**Authors:** Andreas Magerl, Simone Gingrich, Sarah Matej, Geoff Cunfer, Matthew Forrest, Christian Lauk, Stefan Schlaffer, Florian Weidinger, Cody Yuskiw, Karl‐Heinz Erb

**Affiliations:** ^1^ Institute of Social Ecology University of Natural Resources and Life Sciences Vienna Austria; ^2^ Department of History University of Saskatchewan Saskatoon SK Canada; ^3^ Senckenberg Gesellschaft für Naturforschung Frankfurt am Main Germany; ^4^ Vienna University of Technology Vienna Austria; ^5^ College of Law University of Saskatchewan Saskatoon SK Canada

**Keywords:** carbon dynamics, wildfires, forest fires, United States, biomass use, fire suppression

## Abstract

Wildfires and land use play a central role in the long‐term carbon (C) dynamics of forested ecosystems of the United States. Understanding their linkages with changes in biomass, resource use, and consumption in the context of climate change mitigation is crucial. We reconstruct a long‐term C balance of forests in the contiguous U.S. using historical reports, satellite data, and other sources at multiple scales (national scale 1926–2017, regional level 1941–2017) to disentangle the drivers of biomass C stock change. The balance includes removals of forest biomass by fire, by extraction of woody biomass, by forest grazing, and by biomass stock change, their sum representing the net ecosystem productivity (NEP). Nationally, the total forest NEP increased for most of the 20th century, while fire, harvest and grazing reduced total forest stocks on average by 14%, 51%, and 6%, respectively, resulting in a net increase in C stock density of nearly 40%. Recovery from past land‐use, plus reductions in wildfires and forest grazing coincide with consistent forest regrowth in the eastern U.S. but associated C stock increases were offset by increased wood harvest. C stock changes across the western U.S. fluctuated, with fire, harvest, and other disturbances (e.g., insects, droughts) reducing stocks on average by 14%, 81%, and 7%, respectively, resulting in a net growth in C stock density of 14%. Although wildfire activities increased in recent decades, harvest was the key driver in the forest C balance in all regions for most of the observed timeframe.

## Key Points

- We present a new data set, reconstructing biomass removals by fire, harvest, and grazing in U.S. forests on a regional scale from 1941 to 2017
- Harvest and fire were the most intensive removals of biomass over the 20th century. Regional trends diverge from national trends
- In the context of natural climate solutions, long‐term and regional dynamics of interconnected drivers of forest biomass change need to be considered

## 1 Introduction

Wildfires have been a key feature of the Earth system for millions of years, affecting a large variety of biogeochemical processes, ecosystems, and organisms (Bowman et al., 2020; Pausas & Keeley, 2009). Fire is caused either naturally (primarily by lightning) or by humans. In most populated places, humans have been the main cause of fire ignitions for hundreds of years (Pyne, 1995) and have fundamentally altered natural fire regimes on Earth (Bowman et al., 2011; Pyne, 1991), with land use being an integral objective and driver for the emergence of human‐influenced fire regimes (Bowman et al., 2020; O’Connor et al., 2011). The interconnections between burned area, climate, human, and other biotic processes are highly complex (Teckentrup et al., 2019). Indeed, many applications of landscape fire are beneficial to human wellbeing, or even necessary for regional subsistence and survival. In the context of anthropogenic climate change, fire activity and its far‐reaching impact on atmospheric and biotic processes have gained increased attention (Keywood et al., 2013; Moritz et al., 2012; Pausas & Ribeiro, 2017). Still, the extent, as well as the quantitative relationships between land use, biomass use, and fire impacts have large uncertainties (Bowman et al., 2020; Chuvieco et al., 2019; Erb et al., 2017; Lauk & Erb, 2016; Pechony & Shindell, 2010; van Marle et al., 2017). With changing climate and enhanced concentration of atmospheric CO_2_, temperatures and droughts are already rising, possibly triggering more frequent fire occurrence and higher fire severity in the future (Flannigan et al., 2009; Xu et al., 2020). Changing climate has diverse impacts on vegetation, including increased biomass productivity and thus more fuel available for burning (Teckentrup et al., 2019). Whether or not fires will increase in a given place may largely depend on local climate, combinations of fire characteristics, geographic location, and human activities in these locations (Archibald et al., 2013).

In light of projected changes in the size, location, and severity of wildfires, on the one hand, and forest C dynamics, on the other hand, it is essential to better understand the interlinkages between changing socio‐metabolic activities and fire patterns. Land use—fire interrelations can consist of fire suppression for management and protection of forests, crops, livestock, people, and infrastructure, or fire ignition by humans, either unintended or for specific purposes, including biome conversion, burning of agricultural residues, land‐use change or shifting cultivation (Chuvieco et al., 2019; Malamud et al., 2005; Marlon et al., 2013; Parisien et al., 2016). In both cases, there may be consequences for ecosystem functioning including factors like biodiversity, ecosystem services provision or changes in carbon cycles (Haugo et al., 2010; Hurteau & Brooks, 2011; Kelly et al., 2020; Parks et al., 2015). Fire regimes are defined by the pattern of fire occurrence in a specific place, including the size, intensity, frequency, ignition source, and fuel types affected. They describe seasonal timing of landscape conflagrations within geographic units that may be local, regional, ecosystem types, or species communities, and time frames that are typically annual but may change over the course of decades, centuries, or millennia (Conedera et al., 2009; Krebs et al., 2010; Pyne, 2019).

Due to the well‐researched land‐use history and the abundance of detailed and long‐term data sources for wildfires, and forest use, as well as forest inventories, the contiguous United States (U.S., 48 states excluding Alaska and Hawaii) offers a unique opportunity to investigate the interrelation between wildfires, socio‐metabolic activities connected to land use, and carbon (C) dynamics in forested ecosystems. Land use and the management of forest ecosystems in the U.S. are inextricably linked to landscape fires (Pyne, 1982). Fire has been used by Native Americans for thousands of years to transform ecosystems for hunting, habitat enhancement, and small‐scale agriculture (Lake et al., 2017; Tom et al., 2023). European settlers adopted and expanded native fire practices and reduced forests largely in favor of agricultural land between 1600 and 1900 (Courtwright, 2011; Gregg, 2010; Hessburg & Agee, 2003; Liebmann et al., 2016). Especially during the 18th and 19th centuries, large‐scale land use changes due to agricultural expansion, as well as clear‐cutting of forests led to an increase in burned area, particularly in the south‐eastern region of the U.S. (Fowler & Konopik, 2007; Pyne, 1997). In the early 20th century, large‐scale fire suppression was introduced to decrease “catastrophic” wildfires, often connected to timber‐harvest activities such as slash and debris burning, spark ignition by railways, arson, and other factors. Together with changing forest management, afforestation, natural resource conservation efforts, and the modernization of local subsistence‐based economies (Fedkiw, 1989; Gregg, 2010), fire prevention and suppression, as well as prescribed burning, drastically reduced fire activities in the contiguous U.S. after the 1930s (MacCleery, 1993; Steen, 2004). Consequently, since the early 20th century, U.S. forests have been in a phase of recovery from depletions in the past in terms of area and C‐density (Magerl et al., 2019), a process denoted as forest transition (Mather, 1992). During industrialization, forest productivity to provide construction materials to the rapidly expanding economy superseded subsistence farming (Gregg, 2010). Thus, forest recovery was driven mainly by commercial timber forests in the Eastern U.S., while in the West, less pronounced regrowth occurred in more diverse ecosystems, including newly reserved national forests and low‐productive shrub‐ or woodlands (Magerl et al., 2019; Steen, 2004).

Globally, changes in forest area and biomass are the result of the complex interplay of natural processes, land‐use change, wood consumption and trade, economic development, government policy adjustment, and changes in fuelwood substitution, and vary by country and region (Gingrich et al., 2019). In this context, the role of fire management is surprisingly understudied (Iriarte‐Goñi & Ayuda, 2018), although the impact of naturally or human‐induced fires on forest biomass, especially at local scales, is well documented (Frost & Sweeney, 2000; Wilson et al., 2021). Many studies have analyzed different aspects of wildfires in the U.S., including the influence of humans on contemporary fire regimes or recent increases in size and severity of wildfires, often in connection to climate change, with a particular focus on the West (Abatzoglou & Williams, 2016; Barbero et al., 2015; Dennison et al., 2014; Gedalof et al., 2005; Malamud et al., 2005; Mitchell et al., 2014; Singleton et al., 2019). A range of studies have investigated the dynamics and interplay between forest biomass changes and disturbances by fire, harvest, insects or windthrow (e.g., Gu et al., 2016; Sleeter et al., 2022; Williams et al., 2016; Zhou et al., 2021) but only for short timescales or specific regions. Other works investigated trends over longer time‐periods, including burned area, emissions or other processes connected to changing fire activity, such as fuel accumulation due to fire suppression policies (Wuerthner, 2006). While satellites provide comprehensive data from the 1980s onwards for the U.S., long‐term studies have mostly focused on the regional to local scales (Grala & Cooke, 2010; Guyette et al., 2003; Syphard & Keeley, 2016), with a particular focus on the American West (Higuera et al., 2015; Littell et al., 2009; Marlon et al., 2012). Only a few studies have quantified long‐term changes in biomass burned for the total contiguous U.S. at the national level or on sub‐national scales (Houghton et al., 2000; Leenhouts, 1998). However, the interlinkage between past fire management and land‐use activities is mostly mentioned only implicitly, and rarely empirically explored. National, or comprehensive multi‐regional, long‐term studies linking land‐use, socio‐metabolic activities, and wildfire trajectories are still rare (Balch et al., 2017; Hawbaker et al., 2013; O’Connor et al., 2011).

This study makes those linkages and quantifies forest biomass burned by wildfires in the contiguous U.S. across nearly a century of significant change. We analyze the relative role of fires in the country's forest C‐balance in comparison to harvested forebiomass (wood for energy and material purposes, and grazing) at the national level from 1926, and at the regional level from 1940 to 2017. We synthesize historical statistics and contemporary satellite data on forest area burned by wildfires and derive fuel loading and combustion completeness factors from field measurements and a fire‐enabled dynamic global vegetation model. We address the following questions: How did wildfires in comparison to biomass extraction contribute to observed changes in biomass C stocks in forests of the contiguous U.S.? In which regions and on which timeframes were wildfires particularly significant? We aim to increase knowledge about the drivers of the change of forest biomass stocks, with implications for management options and models of future fires, which can then be used for climate change mitigation and adaptation strategies (Ford et al., 2021; Rogers et al., 2020).

## 2 Data and Methods

### 2.1 Input Data

We reconstructed burned area for the contiguous U.S. by synthesizing and integrating information from historical reports from the United States Forest Service (USFS) and contemporary satellite data (Table 1). We collected and compared the most complete and widely used data‐sources for burned forest area available for the U.S. for different spatial levels and timescales: on the national scale from 1926 to 1984 from the United States Bureau of the Census (1975, 1984), denoted hereafter as “Bureau of the Census” and “Statistical Abstract,” on the scale of broad geographic regions (Northeast, Southeast, Rocky Mountains (RM), and Pacific Coast (PC)) from 1938 to 1979 by Ciesla and Mason (2005), on the state level from the USFS's *Forest Fire Statistics* for several years between 1938 and 1964, and from the *National Forest Fire Reports* and *Annual Fire Report for the National Forests* for 1971–1984, hereafter denoted as “USFS reports.” For the period 1985–2017, remote sensing data were available at high spatial resolution (Hawbaker et al., 2020), denoted as “Landsat data”.

**Table 1:** Data Sources for Reconstructing Burned Forest Area in the Contiguous U.S. 1926–2017 on the National and Regional Scale

Source	Abbreviation	Spatial level	Min. fire size (hectare)	Type of publication/data	Ownership	Land cover type	Protection status	Data points per decade									
								1920s	1930s	1940s	1950s	1960s	1970s	1980s	1990s	2000s	2010s
United States Bureau of the Census^a^, Statistical Abstract of the United States^b^	Bureau of the Census	National	<0.4	Statistical compilation, based on USFS agency reports	Federal	Forest	Protected	4^c^	10^c^	10^c^	10^c^	10^c^	6^j^	4^d^	0	0	0
						Other forest^e^	Protected	0	9^c^	5^c^	0	0	0	0	0	0	0
	Statistical Abstract				State and private	Forest	Protected	4^c^	10^c^	10^c^	10^c^	10^c^	6^c^	4^d^	0	0	0
							Unprotected	4^c^	10^c^	10^c^	10^c^	10^c^	6^c^	4^d^	0	0	0
United States Forest Service, Forest Fire Statistics^f^, Annual Fire Reports for the National Forests^g^, National Forest Fire Report^h^	USFS reports	State	<0.4	Agency reports	Federal	Forest	Protected	0	1^d^	8^c^	6^c^	8^c^	9^c^	6^d^	0	0	0
							Unprotected	0	0	0	3^c^	0	0	0	0	0	0
						Other land inside national forest boundaries^e^	Protected	0	1^d^	8^c^	6^c^	8^c^	9^c^	6^d^	0	0	0
					State and private	Forest	Protected	0	0	7^c^	6^c^	0	0	0	0	0	0
							Unprotected	0	0	7^c^	6^c^	0	0	0	0	0	0
						Other forest^e^	Protected	0	0	7^c^	6^c^	0	0	0	0	0	0
						Unknown/other land^e^	Unprotected	0	0	6^c^	6^c^	0	0	0	0	0	0
Ciesla and Mason^i^	Ciesla and Mason	Regional	<0.4	Study, based on USFS reports	Aggregated	Forest	Not specified	0	2^d^	9^c^	10^c^	10^c^	8^c^	0	0	0	0
USGS Landsat Burned Area^j^	Landsat data	Spatially explicit data (30 m), intersected with state and ownership boundaries	0.4	Satellite data	Federal, state and private	Forest (deciduous, evergreen, mixed)	Not specified	0	0	0	0	0	0	5^c^	10^c^	10^c^	9^c^
						Shrub/scrubland		0	0	0	0	0	0	5^c^	10^c^	10^c^	9^c^
						Herbaceous		0	0	0	0	0	0	5^c^	10^c^	10^c^	9^c^

^a^ United States Bureau of the Census (1975).

^b^ U.S. Bureau of the Census (1984); U.S. Department of Commerce and Labor (1984, 1908, 1921, 1930).

^c^ Used for reconstruction.

^d^ Used for comparison and validation.

^e^ Assume shrub/scrub/woodland.

^f^ United States Forest Service (1941, 1942, 1945, 1946, 1947, 1948, 1949, 1950, 1951, 1954, 1955, 1956, 1958, 1964b).

^g^ United States Forest Service (1960, 1961, 1962, 1963, 1964a, 1965, 1966, 1969).

^h^ United States Forest Service (1971, 1972, 1973, 1974, 1975, 1976, 1977, 1978, 1979, 1980, 1981, 1982, 1983, 1984, 1985).

^i^ Ciesla and Mason (2005).

^j^ Urbanski et al. (2018).

#### 2.1.1 USFS Agency Reports

The original printed USFS reports were available for the years 1938, 1941, 1942, 1945–1951, 1954–1956, 1958, 1964, and from 1971 to 1985 (Table 1). For these respective years, they report burned area on the state level for “federal,” and “state and private” forests, but also for a small share of ecosystems with sparse or no tree cover (wood‐, shrub‐, scrublands), called “other forests” or “other land inside national forest boundaries.” Houghton et al. (2000) assumed in their study that all of these reported burned areas referred to forests. However, although other forests represent only a relatively small share of the reported total burned area, they also contain woody perennial vegetation storing carbon over more than 1 year. Thus, they also contribute to total C stocks in above‐ and belowground biomass, but to a much lesser extent than productive forests.

To account for these differences, we consider three different land‐cover types, based on information in the USFS reports and from USFS forest category definitions (Oswalt et al., 2014): “state and private” forests are almost entirely commercially used productive timber forests, while “federal forests” subsume commercial and non‐commercial forests under federal administration, including national and reserved forests. We assume “other forests and other land inside national forest boundaries,” hereafter simply termed “other forests,” to be areas with sparse woody vegetation (wood‐, shrub‐, and scrubland). The USFS reports stopped publishing burned area for protected and unprotected “state and private forests,” as well as unprotected “federal forests” and “other land” (which represent only a small share of total burned area) after 1960, whereas burnt area statistics for protected “federal forests” were reported for the whole period. Aggregated national scale “forest fire” data were reported by the United States Bureau of the Census (1975, 1984) for the period 1926–1984 in *Historical Statistics of the United States—Colonial times to 1970* and the *Statistical Abstract of the United States*. Both sources are compiled from USFS reports. We used these data sources to reconstruct burned area on the national scale.

#### 2.1.2 USGS Landsat Burned Area

The U.S. Geological Survey's (USGS) *Landsat Burned Area Product* for the period 1985–2017 is based on data acquired since 1985 by the Landsat 5, 7, and 8 satellites (Hawbaker et al., 2020), reported by land‐cover type as defined by the USGS *National Landcover Database* (NLCD, Homer et al., 2012). Each of the Landsat satellites has a revisit cycle of 16 days, which is too long for tracking active fires. Hence, their burned area estimates are based on spectral indices known to be sensitive to recently burned vegetation. These indices are, however, subject to uncertainties due to other factors relating to vegetation dynamics or environmental conditions affecting the observation, for example, shadowing by clouds or topography (e.g., Escuin et al., 2008). Cloud cover can further add to the uncertainty and incompleteness of the record of wildfire events (Hawbaker et al., 2017). Data are provided as date‐specific burned area classified rasters with a spatial resolution of 30 m and as yearly composites in vector format. The latter was used in our estimation. It provides polygons of contiguous patches burned during a year with derived information, such as date of first detection, burn probability and the number of pixels of a burn patch belonging to each NLCD class. The burned forest and shrubland area used in this study was aggregated by year and state. See Text S1 in Supporting Information S1 for more details.

### 2.2 Reconstruction of Burned Area

**Figure 1:**
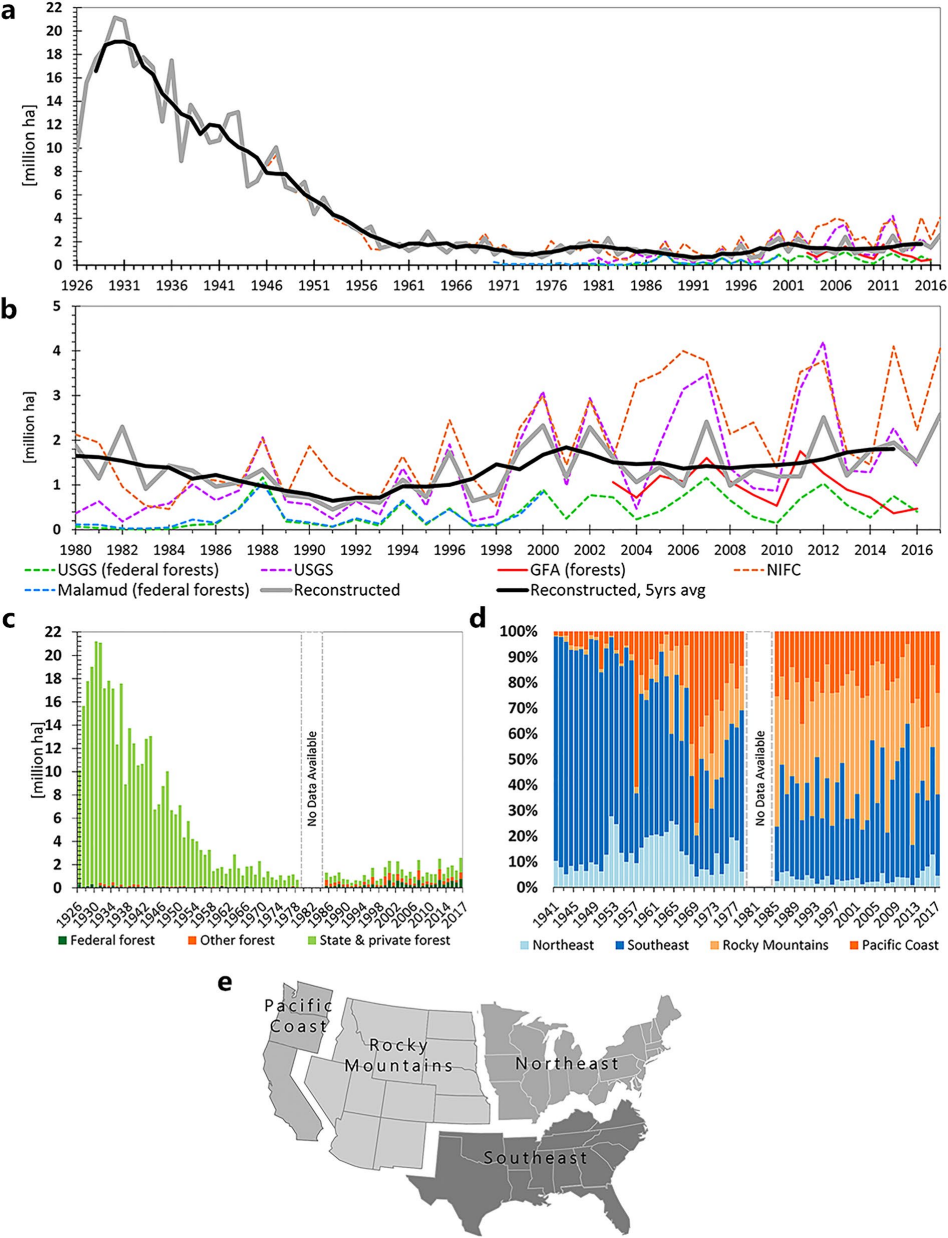
Reconstructed burned area for the contiguous U.S. total (gray line) and 5 years moving average (black line) (Combined data by Bureau of the Census, United States Forest Service reports, Ciesla and Mason, Landsat, see text and Table 1 for details) compared to other burned area products for the U.S. in million ha. Reconstructed U.S. burned forest area, national total (a) 1926–2017, (b) 1980–2017, (c) 1926–2017 by forest category, million ha, (d) 1941–2017 by regions, shares of total burned forest area. (e) Map showing the four study regions, adapted from Oswalt et al. (2018). *Note*. In panels (a) and (b), agency reports or field observations are depicted as dashed lines, remote sensing, and reconstructed series appear as solid lines. In panels (c) and (d) data gap 1980–1984. No data available for “Federal” and “Other forest” on the regional scale in 1943, 44, 52, 53, 57, 59, 67, 68, 70. See text and Table 1 for additional details.

We combined the national aggregated Bureau of the Census and Statistical Abstract data for the years 1926–1984 (in acres, converted to hectares) with the Landsat data (1985–2017, in hectare) to produce a continuous annual burnt area reconstruction in hectares (ha) for the years 1926–2017 on the national scale. Furthermore, we used the state‐level USFS reports 1941–1985 as a starting point for the sub‐national reconstruction since they represent the most detailed historical data in terms of land‐cover types, ownership, use, and spatial disaggregation. From 1960 onwards, burned area reports for protected and unprotected “state and private forests” were discontinued in the USFS reports. Therefore, we used data by Ciesla and Mason (2005) to derive this “missing” portion. This publication provides burned area in “total forest ecosystems” on regional scales (aggregated Northeast, Southeast, Rocky Mountain, and Pacific Coast regions, see Figure 1d) from 1938 to 1979. It contains only aggregated burned forest area and does not provide detailed information about the forest categories burned. Nevertheless, according to William Ciesla (personal communication), the numbers reported in these publication stem from the original USFS reports as well. Hence, we aggregated the USFS state‐level burned area data to the four regions (Figure 1d) and subtracted them from the data by Ciesla and Mason for the period 1938–1979 for the same regions, yielding the missing fraction of state and private forest area burnt. For the period 1985–2017, we aggregated the spatially explicit Landsat data to the same regions: First, to match the forest categories reported in the USFS reports (1941–1985) with the more recent satellite‐based data from 1985 to 2017, we aggregated the respective “forest,” and “shrubland” NLCD categories (see Table S1 in Supporting Information S1) in the Landsat data. Next, we intersected these data with state‐area boundaries and administrative units (“state and private,” “federal”) from the USGS using ArcGIS. This way, we reconstructed regional burnt area for the years 1941–1979 and 1985–2017. The period 1980–1984 was excluded from the regional analysis. Comparison of total aggregated burned area for the overlapping years for USFS reports, Bureau of the Census (1975), Statistical Abstract (1985), and Ciesla and Mason (2005) showed excellent agreement (96%–100%). Hence, we consider the latter data source complete and useful for reconstructing the burned area.

### 2.3 Validation Data

Besides the main data sources used for reconstruction and validation (Table 1), we collected additional burned area products for the U.S.: The *National Interagency Fire Center* (NIFC) (National Interagency Fire Center, 2020), the *USGS Federal Wildland Fire Occurrence database* (USGS) (T. J. Brown et al., 2002), a study on federal forest fires by Malamud et al. (2005), and the *Global Fire Atlas* (GFA) (Andela et al., 2019). The GFA is another remote sensing product based on gridded data with 500 m spatial resolution, whereas all other sources contain field observations from various agency reports. For comparison with the historical sources, we excluded agricultural and built‐up infrastructure from the GFA databases. The NIFC contains information for “wildland fires,” which refers to all land‐cover types except agricultural and built‐up land, according to Houghton et al. (2000). The USGS database (1980–2015) combines fire records from five federal agencies, the *Bureau of Land Management*, the USFS, the *Bureau of Indian affairs*, the *Fish and Wildlife Service*, and the *National Park Service*. They monitor wildfires on various federal owned land‐cover types, such as forests, rangelands, and deserts across the contiguous U.S., excluding all fires on non‐federal lands. The study by Malamud et al. uses processed data by the USGS for federal forests.

### 2.4 Estimation of Burned Biomass

We estimate the biomass burned by wildfires by multiplying the reconstructed burned area with fuel loads, and combustion completeness factors. Fuel loads refer to the amount of total biomass within a land‐cover type susceptible to fire, whereas combustion completeness factors are coefficients used to estimate the share of the respective fuel loads that actually burn in a fire event. Stenzel et al. (2019) argued that default combustion completeness factors are prone to severely overestimate biomass burned, and thus that case‐study specific field measurements of combustion completeness and fuel loads (van Leeuwen et al., 2014) should be preferred.

We obtained average contemporary forest fuel loads in tons dry matter per hectare and year (t dry matter/ha/yr) based on field measurements from 2003 to 2015 from Urbanski et al. (2018), specified by compartments: (a) surface fuels consisting of duff/litter and deadwood, delineated by fuel moisture time‐lag categories (1, 10, 100, 1,000 hr). The time‐lag represents the fuels' response to changing weather conditions and thus their relative dryness (i.e., a 1 hr fuel has dried out 1 hr after a rain event) (b) live fuels, consisting of grasses/herbs, as well as canopy components (leaves, branches, stems). Using a forest type map by Ruefenacht et al. (2008), we allocated fuel loads to the four aggregated regions. To account for the different biomass characteristics of the ecosystems under investigation (low‐productivity wood/scrub/shrublands vs. productive timber and federal forests), we allocated fuel types by forest categories and region as proxies for the available total fuel loadings (see Table S2 in Supporting Information S1). We assumed that all types of fuels for all strata (duff to canopy) would be present in productive forests (“federal,” “state and private”). For “other forests,” we assumed that duff/litter, 1 hr deadwood (small branches from dead shrubs), and grasses/herb fuels would be present.

We applied the fire‐enabled dynamic vegetation model LPJ‐GUESS‐SPITFIRE (Rabin et al., 2017; Thonicke et al., 2010) to estimate burned biomass. Compared to the model described in Rabin et al. (2017), the version used here includes an updated LPJ‐GUESS vegetation model which simulates nitrogen cycling and nitrogen limitation on plant growth (Smith et al., 2014). In addition, the calculation of human ignitions was adjusted to ensure that the model reproduced global remotely sensed burnt area. An additional suppression of fire size due to landscape fragmentation caused by croplands was added to improve the spatial patterns of burnt area. However, the parts of the fire model relevant to this study, the fuel moisture calculation and the combustion completeness calculation, were unchanged from the original formulation of Thonicke et al. (2010). We used the model to calculate annual fuel characteristics by fuel component and region for the period 1941–2017. Next, we applied the yearly change coefficients estimated by LPJ‐GUESS‐SPITFIRE for this period to the contemporary fuel loads from Urbanski et al. (2018) by region to derive a modeled fuel‐characteristics timeline (Figure S1 in Supporting Information S1). This way, instead of assuming static values, we accounted for changing climatic conditions influencing fuel loads. For the regional estimations of burned biomass we used the respective combustion completeness factors, also modeled by LPJ‐GUESS‐SPITFIRE (see Figure S2 in Supporting Information S1). For national level burned biomass from 1926 to 1940, we extrapolated average fuel‐loadings and combustion completeness factors from 1941. Finally, dry matter of burned biomass was converted to tons carbon (C) using an average conversion factor of 0.5 (Schlesinger, 1997).

### 2.5 Forest Biomass Removals and C Stocks

To assess the relative role of forest fires and other factors influencing biomass C‐stocks, such as changes in land use and resource consumption, we collected and integrated data on biomass harvest, forest grazing, and C‐stocks for the contiguous U.S. Due to the lack of consistent long‐term data, in this assessment we did not include information on other extreme events, such as windthrow or bark beetle infestation. Data for biomass removals, that is, wood harvest (timber, fuelwood) and grazing by livestock, were obtained from Magerl et al. (2022).

Total wood harvest includes forest biomass removed for industrial wood products, including lumber, pulpwood, plywood, veneer and other products, as well as fire‐ and fuelwood. The original data from Magerl et al. (2022) stem from a collection of all known harvest estimates for the U.S. and was used to create a consistent time series by calculating means after plausibility checks (see Figure S5 in Supporting Information S1). The raw data used here stem from the USFS timber inventories (approximately every 10 years, 1953–2017), which provide yearly average harvest levels between inventory points. Additional information for the earlier years stems from pre‐inventory assessments by the USFS for 1907, 1920, 1932, and 1945, as well as from the United States Bureau of the Census (1975). To account for biomass losses during harvest (such as bark, branches, leaves, below‐ground biomass), we used biomass expansion factors from Krausmann et al. (2013). See Text S2 in Supporting Information S1 and Magerl et al. (2022) for details. Forest grazing was estimated using statistical data for livestock numbers and available feed supply (feed crops, pasture, hay) from the USDA Agricultural Census, and species‐specific feed demand (Krausmann et al., 2013). See Magerl et al. (2022) for detailed methods and results for grazing.

Total forest area and biomass growing stock by state, ownership, tree species and forest category were obtained from the decadal USFS timber inventories by Magerl et al. (2019) for the period 1907–2012. Forest biomass stocks were expanded for above‐ and belowground biomass, including deadwood and litter stocks, using C‐content by species with ecoregion‐specific IPCC factors (Eggleston et al., 2006). See Magerl et al. (2019) for additional details.

All results were updated to 2017 and converted to tons C using a C‐content factor of dry matter biomass of 50%. To account for the large yearly variation in burned biomass, we calculated the 5‐years moving average for comparison with the decadal average biomass removals and C stock values. For better comparability of pressures on forest ecosystems inflicted by removals at various spatial scales, all biomass fluxes (harvest, grazing, fires) and C stocks were expressed per unit area (tC/ha).

### 2.6 Analysis

To identify periods and regions where fire or the other removals were important for the observed forest C stock development, we investigated the relation of per‐area burned biomass, forest grazing, harvest, and net biomass stock changes by means of sub‐national C balances. A C balance provides information on the contribution of basic gross processes that cause changes in C stocks in forests. These processes include additions to biomass through growth, removals through harvesting and mortality mechanisms such as wildfires, windthrow and age‐related decline. Following an approach by Williams et al. (2016), we quantified, on the national scale, as well as on the four sub‐national regions, the net change in forest biomass stocks as well as biomass removed from forests through harvest, grazing and fire, at 10 year time intervals (except for the period 1926–1930 on the national scale and 1970–1977 on all scales). This makes it possible to directly attribute changes in C stocks to key processes influencing them. Together, the sum of the observed biomass stock changes and the removals of biomass can be used as a proxy for the net ecosystem productivity (NEP), which is defined as Net Primary Production (NPP) of forests minus heterotrophic respiration (Ghimire et al., 2012; Schulze et al., 2021). NEP indirectly reflects the different regional growth and mortality rates in forests due to age structure and recovery from past disturbances (Mantgem et al., 2009; Zhang et al., 2012). Positive stock changes reflect net growth in forests that is, when yearly increase exceeds natural mortality and disturbances. Negative stock changes, on the other hand, indicate that mortality rates were higher than annual growth, implying that factors other than those analysed here were responsible for net C losses, such as insect infestations, windthrow, or adverse environmental conditions, such as droughts.

#### 2.6.1 Sensitivity Analysis

We calculated multiple alternative estimates for biomass burned to assess the sensitivity of our results. For this purpose, we used different combinations of factors for fuel loadings (modeled, field‐measurements, static vs. dynamic over time) and combustion completeness factors (static low, moderate, high severity) from the literature (Yang et al., 2015) versus dynamic fuel loadings modeled by SPITFIRE. We re‐estimated four main variations including three sub‐variants each on the regional scale, resulting in a total of 48 variations of burned biomass on the regional scale for 1941–2017. Additionally, we re‐estimated burned biomass using average fuel loadings for (a) separate forest categories burned and (b) aggregated forest area burned. This way, we tested our results for regional variations, temporal evolution of fuel loads, and scaling effects, and assessed the influence of variables derived from model versus field‐measurements. We determined the sensitivity of our reconstructed wood harvest time series comparing our estimate with other published data series (see Figure S5 in Supporting Information S1). We also modified the expansion factors used to calculate harvest losses (total biomass in live trees lost due to harvest). We used U.S. minimum and maximum factors for the ratios of bark and other compartments (e.g., twigs, leaves) compared to harvested stem volume, as well as the ratio of belowground to aboveground biomass from Kastner et al. (2022), separately for fuelwood and for industrial wood products. The sensitivity range for forest grazing was obtained from Magerl et al. (2022). See Text S8 in Supporting Information S1 for estimation details and full results.

## 3 Results

### 3.1 Burned Area

Figures 1a and 1b present the reconstructed total absolute (solid gray line) and 5 years moving averages (solid black line) burned forest area for the contiguous U.S. in comparison to other available burned area data sources. Trends in burned forest area are similar in all series, with our reconstructed estimates reporting the highest values throughout the period (Figure 1a). Compared to our reconstruction, average burned areas reported in the GFA 2003–2016 were lower by 43%, while those reported by the U.S. Geological Survey (USGS, only forests) 1980–2015 and by Malamud et al. (2005) 1980–2000 were lower by 52%. USGS total burned area was higher because it includes other land‐cover types such as grasslands, rangelands, and croplands under federal administration, as well as area burned by prescribed fires. USGS burned forest area includes only federal forests and was thus lower than our reconstruction. Until the 1970s, the NIFC, Bureau of the Census (1975) and our reconstruction show almost the same numbers (Figure 1a). After the 1970s, NIFC data diverge from our reconstruction, but its original sources for the period 1983–2017 were not referenced in the online source (https://www.nifc.gov/fire-information/statistics/wildfires). However, the differences can be explained by the fact that it also includes burned area for Alaska and Hawaii, and similar to the USGS, other land‐cover types such as grasslands (Houghton et al., 2000), whereas we limited our reconstruction to forests, wood‐, shrub‐, and scrublands in the contiguous U.S. Compared to the MODIS data, on which GFA is based (500 m), our reconstructed burned forest area is higher, due to the higher spatial resolution of the Landsat data (30 m) which captures also small fires (minimum fire size 0.9 ha in Landsat vs. 21 ha in GFA). While the reconstructed historical burned area shows almost perfect agreement with the NIFC (complete overlap between 1926 and 1945, Figure 1a), for 1985–2017 Landsat yielded partly diverging values compared to the USGS (only forests), GFA, and Malamud data, due to different land‐cover and spatial coverage, as explained above. Nevertheless, the national trends of our reconstruction, NIFC, and the USGS, albeit on different levels, follow similar patterns (Figure 1b).

Burned forest area (Figure 1c) on the national scale decreased significantly between 1926 and 2017. In total, burned area shrank from a maximum of 21 million hectares (Mha) in 1931 to 0.4 Mha in 1991 but then increased to 2 Mha in 2017. On average, between 1926 and 1978, 7 Mha of forests burned annually across the contiguous U.S. The burnt area fraction during this timeframe was a minimum of 0.27% in 1975 and a maximum of 8% in 1930 and 1931. For a large part of the observed period, state and private forest was the most fire‐prone forest category (Figure 1c) and accounted on average for 81% of the total burned area (minimum 35%, maximum 99%). The burnt area fraction in state and private forests between 1926 and 1978 was on average 4% (minimum 0.32% in 1975, maximum of 11% in 1930). This fraction decreased to a maximum of only 0.62% from 1985 to 2017. Federal and other forests seem to have burned significantly less in the beginning of the observed timeframe but their relative share in total forest area burned, compared to state and private forests, increased significantly in the last decades of the period (Figure 1c, Figure S5 in Supporting Information S1). This is probably due to more accurate reporting of burned area via remote sensing data (Chuvieco et al., 2019; Short, 2015) compared to early field observations, which did not report some fires in woodlands (Loehle, 2020).

Total burned forest area by region (Figure 1d) varied throughout the period but reveals a general pattern of strongly decreasing fire activity in the eastern regions and moderately decreasing fire activity in the western regions. The Southeast accounted for 16%–90% (average 65%) of the total burnt forest area from 1941 until the 1970s. Its share in total burned forest area decreased significantly, from 80% in the 1940s to 58% between 1955 and 1967, then further to 38% between 1967 and 1985. Between 1985 and 2017, it remained around 34%. The USFS state‐level data reveal that an average of 54% of the total burned forest area in the region, between 1941 and 1960, occurred in just three south‐eastern states: Florida, Georgia, and Mississippi (Figure S3 in Supporting Information S1).

Most burned area in the Southeast and Northeast occurred in state and private forests, with the former representing on average 99% of the total burnt area between 1941 and 1978, and still 81% on average between 1985 and 2017 (Figure S4 in Supporting Information S1). In the Northeast, these shares were similar. However, since the 1980s, the relative share of federal forests in total burnt area in these regions has risen to around 25% in the Southeast in 2017 and 30% in the Northeast, respectively. In contrast, forest area burned was more heterogeneously distributed across forest categories in the western regions throughout the entire time‐period.

### 3.2 Wildfires, Wood Harvest, Forest Grazing, and Carbon Stock Change in Forests

**Figure 2:**
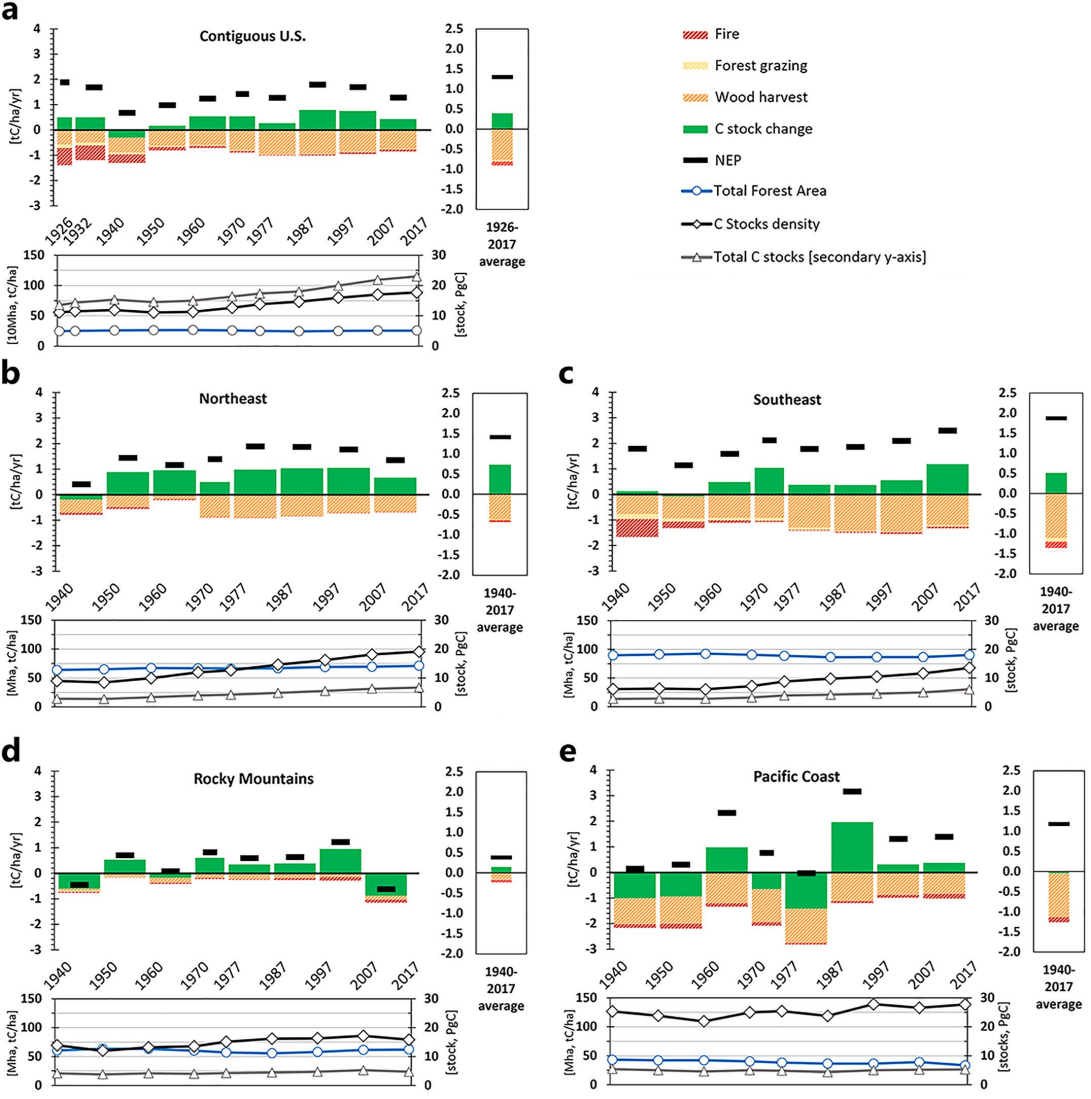
C balance of contiguous U.S. forests consisting of net ecosystem productivity (NEP), forest biomass removals (fire, wood harvest, forest grazing), and C stock change per total forest area in tC/ha/yr for 10 year intervals between 1926 and 2017 on the national scale (a) and between 1940 and 2017 on the regional scale (b–e) (upper panels) and averages per total forest area in tC/ha/yr for the same periods and regions (right panels); Total forest area in 10 Mha (a) and Mha (b–e), C stock densities in tC/ha (primary *y*‐axis), and total biomass C stocks in PgC (secondary *y*‐axis) at points in time (lower panels) *Note*. Data for forest grazing, wood harvest, forest area, and C‐stocks obtained from Magerl et al. (2019, 2022) and updated for 2017 using data from the latest United States Forest Service Forest Resources Assessment Oswalt et al. (2018).

Figure 2 displays the biomass C balance of forests in the contiguous U.S., integrating removals of biomass by fire, harvest and grazing, plus actual biomass C stock changes, representing Net Ecosystem Production (NEP). All values in the upper and right panels are displayed per total forest area in tons C/ha/year, on the national (1926–2017, Figure 2a) and regional scales (1940–2017, Figures 2b–2e), for time periods (upper panels) and as averages over the total time periods (right panels). Lower panels display the dynamics of stock densities (tC/ha), total forest area (Mha) and total forest biomass stocks in petagrams C (PgC, secondary *y*‐axis) for the respective points in time.

C stocks in forests of the contiguous U.S. increased consistently throughout the observed time‐period because NEP (black bars) exceeded the sum of removals. On the national scale (Figure 2a, upper panel) and averaging over 1926 and 2017, NEP increased forest carbon stocks by 1.40 tC/ha/yr, while harvest, fire, and grazing reduced stocks by 0.72, 0.20, and 0.06 tC/ha/yr, respectively. Consequently, the average net C balance of forests (Figure 2a, right panel) was positive with an average C sequestration rate (stock change, green bars) in this period of 0.39 tC/ha/yr. In nearly all timeframes analyzed from 1926 to 2017, national scale biomass growth exceeded removals, resulting in a net increase in C stock density for much of the twentieth century that fluctuated between 0.17 and 0.79 tC/ha/yr. The only exception was 1940–1950, when removals exceeded biomass growth and C stock density decreased by 0.31 tC/ha/yr. Harvest was the largest removal of C during the observed periods in all regions (between 0.09 and 1.45 tC/ha/yr), while fire was the second largest removal, ranging between 0.01 and 0.70 tC/ha/yr. Biomass removals by fire decreased by almost 90% from 1926 to 1970, stayed relatively unchanged for the next 30 years, and slightly increased in recent decades. Harvest increased until the 1970s, then decreased again slightly until the end of the observed timeframe. The largest regional average reductions of forest biomass carbon (Figures 2b–2e, right panels) occurred in the Pacific Coast (PC, Figure 2e) and Southeast (SE, Figure 2c), with harvest offsetting, respectively, 90% and 59% of gross biomass growth. In all regions, harvest declined after the end of the 20th century, while fires increased. In the SE between 1940 and 1950, and in the Rocky Mountains (RM, Figure 2d) between 1997 and 2017, removals by fire exceeded harvest.

The national C stock density increase was driven by C accumulation in eastern forests. Although all regions observed experienced net stock gain over the entire timeframe, the Northeast (NE) and SE contributed the largest average gains, respectively 0.74 and 0.51 tC/ha/yr over the period 1940–2017 (Figures 2b and 2c, right panels). These regions' stock change rates were almost entirely positive over the analyzed timeframes, with small net stock losses of −0.19 tC/ha/yr occurring between 1940 and 1950 in the NE (Figure 2b, upper panel) and −0.08 tC/ha/yr between 1950 and 1960 in the SE (Figure 2c, upper panel). Per area harvest pressure as well as NEP in the NE were substantially lower and stable compared to the SE, where both indicators increased over much of the 20th century.

In the west, decadal C flows fluctuated and their average contribution to the observed increase in national C stock density was substantially lower in contrast to eastern forests because gains and losses almost balanced each other out, resulting in an average net C stock change over 1940–2017 of −0.05 tC/ha/yr in the PC (Figure 2e, right panel) and of 0.15 tC/ha/yr in the RM (Figure 2d, right panel). Considering frequent stock losses caused by factors other than the ones analysed here (bark beetles, drought), the gains and losses of C in the RM almost balanced each other out; in 3 out of the 8 analyzed time‐periods, the RM experienced net stock losses, fluctuating between −0.17 and −0.88 tC/ha/yr (Figure 2d, upper panel). In this region, between 1997 and 2017, harvest and fire removed similar amounts of biomass (on average 0.14 tC/ha/yr and 0.15 tC/ha/yr respectively). In the PC (Figure 2e, upper panel) stock change was negative in half of all the analyzed time‐periods, ranging between −0.65 and −1.42 tC/ha/yr. Notably, while in the PC net stock losses occurred in the first 50 years of the 20th century, in the RM the strongest losses occurred in the last 30 years of the period, especially in the most recent years.

### 3.3 Uncertainty Analysis

**Figure 3:**
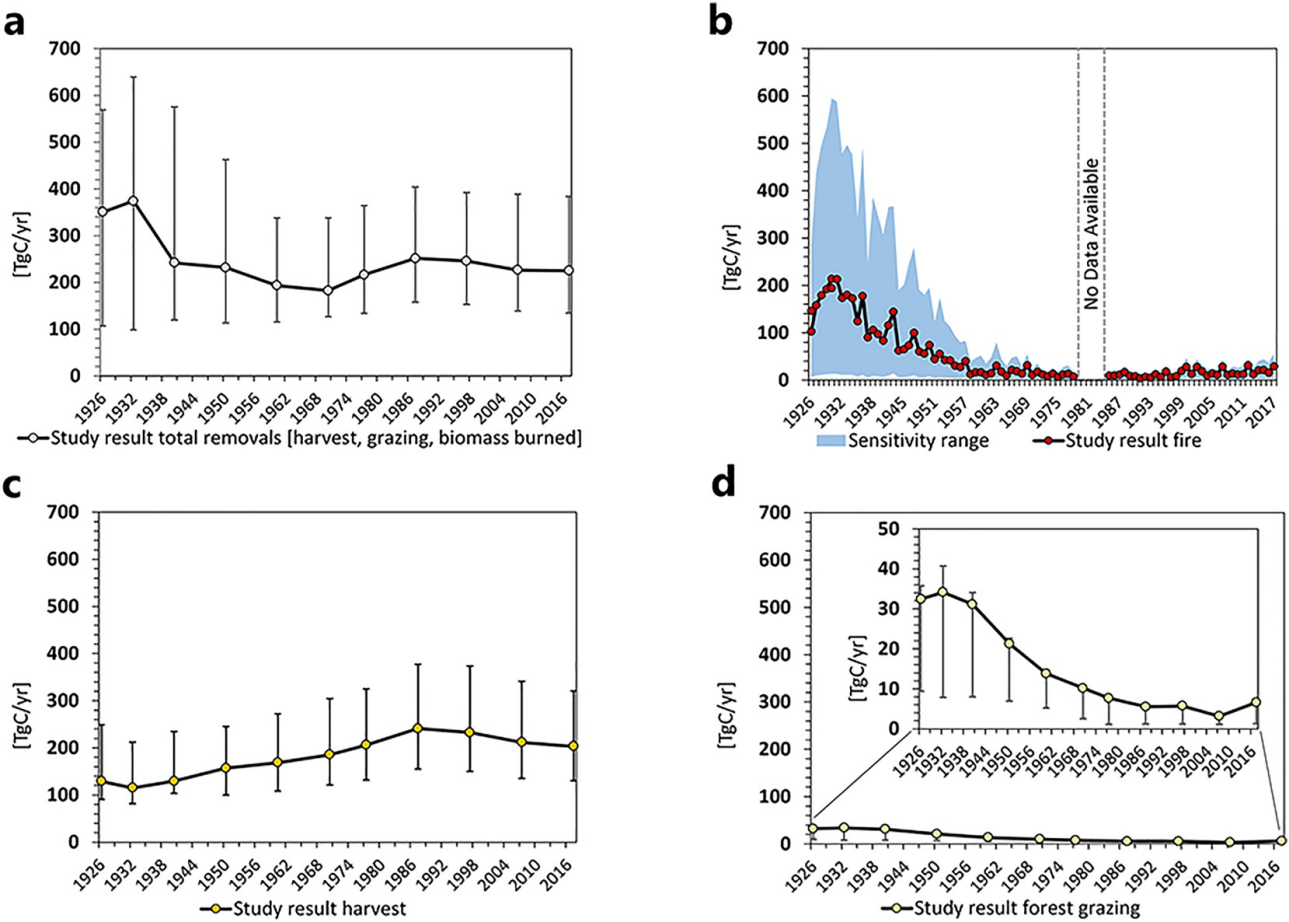
Sensitivity ranges (whiskers, area) for study estimates on the national level for (a) all forest biomass removals combined (fire, harvest, forest grazing), (b) burned biomass, (c) total wood harvest (fuelwood, industrial wood) including harvest losses, (d) forest grazing. *Note*. Data in panels (a, c, d) at 10  year intervals (except for 1926–1930 and 1970–1977) and annual intervals in (b). 1980–1984 data gap in (b).

To test the sensitivity of our results, we calculated alternative minimum and maximum variants for biomass removals caused by fire and harvest using different factors (see Text S8 in Supporting Information S1 for more details). The uncertainty range for forest grazing was adopted from Magerl et al. (2022). The sensitivity analysis (Figure 3) reveals an average uncertainty range for total combined forest biomass removals (Figure 3a) of +160/−103 Teragram Carbon per year (TgC/yr) over the 20th century (maximum +374 TgC/yr in 1932, minimum −144 TgC/yr in 1960). Our burned biomass estimation (Figure 3b) has the highest uncertainty range of all biomass removals. The largest maximum variation was on average 4 times higher (+220 TgC/yr) than the study result (Figure 3b), while the lowest estimation was on average 11 times smaller (−75 TgC/yr) than the study estimate. In general, using low‐to‐high combustion completeness factors from the literature, and excluding duff fuel loads exerted the largest impact on our results. Using average fuel loads instead of specific factors for each forest category and static instead of dynamic fuel loads caused the least variation in overall results.

The lower and upper boundaries of the sensitivity range for biomass burned represent hypothetical constant low and high fire intensities. Maximum intensity would assume that in each fire event, up to 99% of the total forest biomass was destroyed. These estimates can be considered extreme and unrealistic outliers because in reality, in most wildfires very few live trees are killed (Ito, 2004; Stenzel et al., 2019). Our analysis confirms that calculations of burned biomass are associated with high uncertainties. The strong deviation of the minimum and maximum values also confirms that published combustion completeness factors may be prone to overestimating the severity of forest fires (Stenzel et al., 2019).

We also modulated the expansion factors used to infer biomass losses of total wood harvest (Figure 3c). In relative terms, the study estimate has the smallest sensitivity range of all removals. The average maximum deviation was 1.6 times our study result, while the lowest minimum deviation was 1.5 times smaller than our estimate. Although our harvest result is a conservative estimate, in absolute terms, the deviations for wood harvest are large, reflecting the strong influence of the choice of expansion factors used to calculate harvest losses.

The average maximum deviation for forest grazing (Figure 3d) is 0.7 times our estimate, while the minimum deviation averaged 4.2 times lower than the study result. As discussed in Magerl et al. (2022), our grazing result represents a progressive estimate. However, since biomass grazed (even in the highest possible variation) represents only a small portion of the total biomass removals, its relative effect cannot distort the overall results.

Overall, this sensitivity analysis underlines the high level of uncertainty of the various components constituting a mass balance, and in particular of burned biomass calculations, as discussed in the literature (Reid et al., 2005; Robinson, 1989). A nuanced understanding of forest conditions is important in all such analyses. Comparison with other published estimates (Table 2) confirms that our estimations lie within a reasonable range, and thus we consider our results to be robust.

## 4 Discussion

### 4.1 Comparison of Results

Our study demonstrates how historical statistical reports, contemporary satellite data, field observations, and dynamic vegetation models can be used to reconstruct the dynamics of wildfires, harvest, forest grazing, and C stocks in forests of the contiguous U.S. for 91 years on the national and 76 years on the regional level. Table 2 reveals that our results are well in line with other published estimates for biomass burned for the U.S. for varying temporal and spatial scales. Thus, our study shows that the combination of modeled factors and field measurements can provide plausible results in comparison with other published estimates and indicates that calculations based on field studies are able to produce similar results as process‐based modeling approaches (van Leeuwen et al., 2014), confirming the robustness of our method.

**Table 2:** Comparison of Published Estimates of Burned Biomass, Wood Harvest, and C Stock Change With Our Results

Study region	Land‐cover	Time	Unit	Value (min‐max)	Study	Estimation approach/data sources of comparative study
Biomass burned						
WUS^a^	Forest	1984–2010	TgC	146–285	Hicke et al. (2013)	Spatially explicit for total above and belowground biomass based on various data sets and satellite observations (Hicke et al., 2013)
				237 (45–620)	*This Study*	
			TgC/yr	5.4–10.5	Hicke et al. (2013)	
				1.2–15.4	*This Study*	
		1997–2010	TgC	100–197	Hicke et al. (2013)	
				154 (30–399)	*This Study*	
			TgC/yr	7.2–14.1	Hicke et al. (2013)	
				1.5–15.4	*This Study*	
CONUS^b^	Forest	2003–2015	TgC	224	Urbanski et al. (2018)	Spatially explicit for deadwood, litter, and live biomass based on various data sets and satellite observations (Urbanski et al., 2018)
				219 (30–408)	*This Study*	
			TgC/yr	7.5–27.6	Urbanski et al. (2018)	
				10.1–31.3	*This Study*	
CONUS^b^	Forest, Rangeland	2001–2010	TgC	197	French et al. (2014)	Regional for canopy, shrubs, herbs, downed wood, litter–lichen–mosses, ground duels (e.g., duff) based on various data sets and satellite observations (French et al., 2014)
	Forest			160 (22–292)	*This Study*	
	Forest, Rangeland		TgC/yr	7.4–41.4	French et al. (2014)	
	Forest			10.1–28.4	*This Study*	
SWUS^c^	Forest	2005		10	Park Williams et al. (2013)	Regional tree mortality by wildfire based on satellite observations and tree ring data (Park Williams et al., 2013)
WUS^a^				9.1 (1–11)	*This Study*	
CONUS^b^		2001–2020		15.9	Sleeter et al. (2022)	Spatially explicit for live biomass and deadwood pools based on an integrated modeling approach (Sleeter et al., 2022)
		2001–2017		17.9 (2.5–33.5)	*This Study*	
WUS^a^		1984–2008		4	Ghimire et al. (2012)	Spatially explicit for live biomass and deadwood pools based on inventory data‐, satellite observations, and modeling
		1985–2008		4.6 (1.4–12.2)	*This Study*	
PNW^d^		1986–2010		0.1–16.6	Zhou et al. (2021)	Spatially explicit for live biomass and deadwood pools based on inventory data, satellite observations, and ecosystem C cycle model
		1986–2010		0.45–5.7	*This Study*	

					Harvest	
CONUS^b^	Tot. + res	2005	TgC/yr	162.4	Harris et al. (2016)	Inferred from mill surveys
		2007		207.6	*This Study*	
CONUS^b^	Ind. + res	2001–2020		61.6	Sleeter et al. (2022)	Annual time series maps
		2002–2017		105.0	*This Study*	
PNW^d^	Tot. + res	1986–2010		10.6–18.5	Zhou et al. (2021)	Modeled
PC^e^		1987–2012		28.9–52	*This Study*	
US50^f^	Tot. no res	1961–2017		101.5	FAO (2020)	Statistical Series (converted to Carbon)
CONUS^b^		1960–2017		102.8	*This Study*	
CONUS^b^	Tot. + res	1952–2016		156.2	Oswalt et al. (2018)	Forest Inventory (converted to Carbon)
		1950–2017		198.7	*This Study*	
US50^f^	Tot. no res	1926–2005		110.7	Gierlinger and Krausmann (2012)	Material flow analysis based on statistical sources
CONUS^b^		1926–2007		104.2	*This Study*	

					∆C stock	
PNW^d^	Forest	1986–2010	TgC/yr	18.5	Zhou et al. (2021)	Stock change
PC^e^		1987–2012		37.5	*This Study*	
US49		2000–2007		239	Pan et al. (2011)	Stock change
CONUS^b^		2002–2007		255	*This Study*	
US49^g^		1990–1999		26	Pan et al. (2011)	Stock change
CONUS^b^		1987–1997		79	*This Study*	
CONUS^b^		1980s		10	Houghton (1999)	Age‐structure accumulation
				230	Hurtt et al. (2002)	
		1977–1987		65	*This Study*	
		2001–2020		126	Sleeter et al. (2022)	Integrated modeling approach
		2002–2017		159	*This Study*	

^a^ WUS = Western US.

^b^ CONUS = contiguous 48 US.

^c^ SWUS = South‐western US.

^d^ PNW = Pacific north‐west (Oregon + Washington).

^e^ PC = Pacific coast.

^f^ US50 = CONUS + Alaska + Hawaii.

^g^ US49 = CONUS + Hawaii.

Although our results largely agree with published estimates, this study presents only an approximation of long‐term fire impacts on forest ecosystems in the contiguous U.S. Due to the underlying data sources, our approach may even be conservative in its calculation of total fire‐induced C fluxes. While comparing agency reports and satellite data (Figures 1a and 1b) revealed good agreement between data sources for 1985–2017, the historical statistics may have underestimated burned area, in particular in federal and other forests (Figure S4 in Supporting Information S1). Nevertheless, despite possible underreportings and inconsistencies in the historical USFS reports (Short, 2015), the data coverage is sufficiently robust to support conclusions about C stocks in American forests.

The total area monitored and protected by the USFS (i.e., “burnable land”) increased considerably during our study period and reached full coverage only by 1990. However, much of the poorly covered reporting areas before 1990 were non‐forested ecosystems such as western native rangelands, as discussed by Houghton et al. (2000) and Short (2015). The interior U.S., and especially the Great Plains sub‐region, were seriously underreported in the USFS data between 1950 and 1969. However, since there were almost no forests and thus comparatively low biomass C stocks in this sub‐region (0.7%–1% of total contiguous U.S. forest area), they are negligible for our analysis of forest fires (Magerl et al., 2019; Oswalt et al., 2018; Reynolds & Pierson, 1941; USDA Forest Service, 1958). The large majority (around 80%) of the total forests in the contiguous U.S. were already covered in the USFS reports by 1926. By the 1940s, full coverage of the national forest area had been achieved. Especially, states of the Northeast and Southeast with large shares in total forest area were relatively well covered (between 70% and 90%) during this period. Poorly covered eastern states (below <50% coverage) represented only 5% of the total contiguous forest area between 1950 and 1960.

The USFS reports include wildfires as well as prescribed burning and harvest slash burning on unprotected land but do not report these activities separately (Short, 2015). The same applies to the Landsat data (Hawbaker et al., 2020). Although it is not possible to quantitatively assess the share of total biomass burning attributable to human action, Kolden (2019) showed that between 1998 and 2018, on average 1 Mha (or >60% of our reconstructed burned area) per year was associated with prescribed and harvest slash burning, over 90% of which occurred in the Southeast and mostly caused by non‐federal entities. According to Short (2015), the actual number of prescribed fires could have been even larger, at least within the first 20 years of our analysis. If human‐induced fires in the contemporary controlled anthropogenic fire regime contributed such a large share in recent years, it is likely that they contributed an even larger share of the total observed fires during the last decades of small‐holder “woodsburning” in the early 20th century (Hart, 1977; Otto, 1983; Otto & Anderson, 1982).

Our harvest estimate is based primarily on USFS harvest data (without residues). Comparison of the raw data with other sources showed good agreement (Text S5 in Supporting Information S1). Our estimates agree well with those of other studies (Table 2), although our figures are mostly higher. This can be attributed to the use of expansion factors to infer above and belowground harvest losses. While our results account for the total biomass killed during harvest (including roots, bark, and foliage), other estimates excluded unused extraction (FAO, 2020; Gierlinger & Krausmann, 2012), calculated harvest on smaller areas (Zhou et al., 2021), or employed unclear definitions for logging residues (Oswalt et al., 2018). Sleeter et al. (2022) argue that satellite‐based estimates, including their own, tend to significantly underestimate harvest removals compared to inventory derived approaches. All harvest figures with comparable timeframes and study regions lie within our estimated sensitivity range.

Results for C‐stock change rates depend largely on the estimation method, spatiotemporal scale, C pools, and ecosystems considered. While results estimated via an age‐accumulation method (Houghton, 1999; Hurtt et al., 2002) deviate markedly from our results, the stock‐change derived figures (Pan et al., 2011; Sleeter et al., 2022), although covering slightly different timeframes or regions, yield similar results.

### 4.2 Limitations

We did not consider C‐fluxes from wildfires in land‐cover types other than forests. We only include forested areas and shrub/scrublands, which account for 15%–40% (average 27%) of the total burned area in the contiguous U.S. (see Figure S10 in Supporting Information S1). All other land cover types (rangeland, agricultural land, infrastructure, wetlands, unused/barren land) are also subject to wildfires. However, since none of these ecosystems contain considerable quantities of perennial aboveground biomass (trees), they are negligible for our analysis of biomass C stock dynamics. Hence, we did not estimate the respective biomass burned for these land cover types. However, for analyses which assess for example annual or seasonal total GHG emissions from biomass burning or include deep soil organic C, they might be of particular interest (see, e.g., Parton et al., 2015).

Since 2002, there is an increase in average burned area identified by Landsat. This coincides with the first years covered by the Landsat 7 and Landsat 8 missions (which became operational in 2000 and 2014 respectively). However, overall temporal trends in burned area for the whole time‐period covered by Landsat were validated by Hawbaker et al. (2020) by means of linear regression with good fit, hence making the data usable for our purpose. Additionally, the increase in burned area had already started in the 1980s. The agency records assessed in this study show similar increases in total burned area (Figure 1b). Despite possible sources of bias and uncertainty, the data sources used represent, to our knowledge, the most widely utilized comprehensive data products for analyzing long‐term trends in national and sub‐national burned area in the contiguous U.S. (Houghton et al., 2000; Littell et al., 2010; Marlon et al., 2012; Syphard & Keeley, 2016; Westerling et al., 2003). Although not perfect, our estimates are robust approximations of actual wildfire activities in U.S. forests for the period 1926–2017, with even higher confidence for the period after 1985.

Due to data limitation (especially for wildfires), we could not comprehensively and empirically establish a C balance for the period of large‐scale timber harvest, that is prior to 1926 (Magerl et al., 2022). However, to consider the different regional land‐use legacies, we consulted historical qualitative sources and other long‐term land‐use analysis for the U.S. In Section 4.3, we discuss our results in light of the different historical land‐use contexts of the four regions.

Besides the studied removals, biomass losses due to windfall, insects, and similar disturbances, as well as other factors like afforestation, intentional and incidental reforestation on abandoned agricultural land, or nutrient deposition represent important factors influencing forest change. We did not account for these factors explicitly due to limited long‐term data availability. According to existing studies, the interannual and regional variability of wind and insect disturbances is large, and net C losses by windthrow are difficult to estimate and attribute (Williams et al., 2016). Additionally, biomass killed by these disturbances may be transferred to the dead organic matter pool that decays slowly, instead of resulting in immediate C losses to the atmosphere (as with wildfires). Nevertheless, removals of forest biomass by these disturbance agents can be significant and are estimated to range between an average of 1–9 TgC/yr for insects and 1–5 TgC/yr for windthrow during the first two decades of the 21st century (Harris et al., 2016; Hicke et al., 2013; Sleeter et al., 2022; Williams et al., 2016).

Another limitation of our study is the investigation of harvested wood use. Wood harvest immediately removes biomass from forests and thus alters C stocks. Whether harvested biomass contributes to net C loss or gain of a country ultimately depends on its final use within the socioeconomic system, and its long‐term climate mitigation effect depends largely on its lifetime and fate at the end of its lifecycle (e.g., re‐use, recycling, decomposition, or burning) (Geng et al., 2017; Gu et al., 2019). Wood burned for heat or power represents a C emission to the atmosphere. These emissions can, in principle, be removed from the atmosphere again through C sequestration in regrowing forests, thus promoting the substitution of fossil fuel with biofuels. However, if and to what extent biomass represents a carbon‐neutral energy source remains unclear and controversial (Erb et al., 2022; Favero et al., 2020) as it depends on many factors such as forest management, rotation periods and size of harvest, as well as the types of forests harvested (Röder et al., 2019). In contrast, wood used as construction material in buildings might represent an important long‐term C sink, substituting emission‐intensive materials such as concrete and thus improving socioeconomic C sinks (Churkina et al., 2020; Mishra et al., 2022). Although in this study we analyzed the regional and temporal trajectories of total wood harvest (timber, fuelwood, other wood products), we did not explicitly investigate the further use and lifecycles of harvested wood products and waste fluxes within the socioeconomic system.

Altered biomass growth conditions due to climate change (i.e., CO_2_ fertilization effect) represent another possible explanation for the observed gains in C stocks (Zhang et al., 2012). However, attributing the contribution of this factor in the C balance is more complex than the other factors analyzed here and highly uncertain. Model estimates for potential NPP (NPP_pot_) do not indicate strong trends and remain inconclusive for the Western U.S. (Text S7 in Supporting Information S1). However, we want to highlight the importance of all the above discussed factors for future research.

### 4.3 Regional C Balance Dynamics and Their Historical Contexts

National‐level (Figure 2a) trajectories of C stocks and fluxes largely reflect the change in USFS conservation policies (Steen, 2004); wildfires as well as forest grazing and harvest decreased between 1926 and the 1960s. Thereafter, the wood harvest level remained stable or even increased during the rest of the period, right alongside the observed increase in biomass C stock density. The respective C balances of the four regions, however, as well as the trajectories of their C stock densities, showed large spatial and temporal variability (Figures 2b–2e). These regional dynamics varied in the context of the particular land‐use histories in each part of the country.

#### 4.3.1 Northeast and Southeast: Fire Suppression, Rejuvenation, and Stock Recovery

As described in the historical literature, already depleted forests in the Northeast (NE) and Southeast (SE) in the first decades of our analysis were the result of strong pressures on forests during the 19th and early 20th century, including extensive clearcutting of mountain slopes and frequent wildfires (Davis, 2003; MacCleery, 1993). Native‐American fire practices, as a tool for biome conversion, hunting, and pest control, as well as land‐use change and agricultural conversion were adapted by Euro‐American settlers in the colonial era (Fowler & Konopik, 2007; Pyne, 1982). Such “woodsburning” fire practices, in combination with large‐scale timber harvest and other disturbances such as invasive species and pests (e.g., chestnut blight in the early 20th century) had contributed to depleted forests, especially in the SE (Gregg, 2010). The introduction of fire suppression policies in the early 1900s led to a large decline in burned area and thus burned biomass. In the SE, this decline indicates a change from human ignition of many small fires to extended human fire suppression, rather than a decline of large, severe fires (Pyne, 1982). The emergence of commercial timber utilization and the modernization of local subsistence economies were connected to fire suppression efforts and declines in forest grazing (Grelen, 1978; Hansbrough, 1961; Jurgelski, 2008; Steen, 2004). Following the large‐scale deforestation of the late 19th century, afforestation and changing forest management led to an age structure effect in the eastern forests: A legacy of past disturbances led to demographic changes in tree populations, creating a regrowth‐sink. The average stand‐age in such clear‐cut and regrowing forests was lower than in old‐growth forests because there were more young trees. By the early twentieth century these young forests had greater biomass growth rates than old‐growth trees, which store large amounts of C but contribute less to annual increment due to slower growth rates (Harris et al., 2016; Pugh et al., 2019; Song & Woodcock, 2003). Consequently, throughout the 20th century some 90% of total forest area in both the Northeast and Southeast consisted of productive, commercial timberland (Magerl et al., 2019) with significantly younger stand ages (especially in the SE) than in the west (Oswalt et al., 2018; Pan et al., 2011). This effect lasted for decades, but was only temporary. As forests aged, the effect declined and became a less significant contributor to C stock gains by the twenty‐first century (Williams et al., 2016).

The C balances presented here reveal that altered forest use patterns and rejuvenation coincided with recovering stocks in the east, creating persistent C sinks from the 1950s in north‐east forests and since the 1960s in south‐east forests. C stock densities in the east increased for much of the second half of the 20th century, coinciding with slightly enhanced growth conditions, reflected by increases in NPP_pot_ (see Figure S7 in Supporting Information S1), arguably due to human‐induced climate change (Haverd et al., 2020). Together with the recovery from past removals in the SE, this allowed for positive net C stock change despite continued high rates of wood harvest. The C balance of the NE forests was more stable. In that region, for most of the observed time‐period NEP, harvest and fire removals were both lower and less dynamic than in the SE. Wood harvest in this region had peaked and declined earlier (late 1800s) than in the SE (Birdsey et al., 2006; Houghton & Hackler, 2000; U.S. Department of Commerce and Labor, 1908). Although the C stock density increased in the NE throughout the observed time‐period, in the last 20 years of our analysis, stock gains were less pronounced than in the SE. This could already reflect a first sign of the subsiding rejuvenation through the age‐structure effect in the NE. In fact, this is in line with previous research showing that simultaneous growth of harvest and C stock density may be feasible only temporarily for few decades (Gingrich et al., 2022).

#### 4.3.2 Rocky Mountains and Pacific Coast: Fluctuating Dynamics and Increasing Fires

The C balances of the western regions were much more dynamic, but stock density increases were more moderate than in the east. Western forests have less homogenous forest structures compared to the east: The PC hosts both highly productive and protected old‐growth forest, whereas the RM contain less commercial timber forests but more low‐productivity shrub‐ and woodland than any other region (Magerl et al., 2019). There were higher average forest stand‐ages in the western than in the eastern U.S. (Oswalt et al., 2018; Pan et al., 2011). Land‐use change and harvest in both western regions have been less extensive than in their eastern counterparts prior to the 1930s (Houghton & Hackler, 2000), hence the contribution of recovery from past harvest to timber regrowth was less significant in this part of the U.S. Together, lower harvest pressures in the past and a larger share of older, unused forests resulted in higher and more stable C densities, but less pronounced C gains. In the PC, relatively high rates of timber harvest occurred during the observed period, although their per‐area intensity never exceeded 1% of stock density (compared to 2%–3% in the SE), exerting lower relative pressure on forests than in the east (Figures 2c and 2e). Compared to the eastern regions, NEP and removal fluxes were large in the PC, and they almost balanced each other out, resulting in relatively stable C stock densities and a slightly negative average net C stock change rate over the period observed.

Largely attributable to climate change (e.g., Abatzoglou & Williams, 2016; Dennison et al., 2014; Kolden, 2019; Westerling, 2006), wildfires increased in the west throughout the observed period. Extended fire seasons and higher temperatures supported increased fire occurrence, size and severity as well as higher combustion completeness, especially in the RM region, but also in parts of the PC, for example, California (Balch et al., 2017; Barbero et al., 2015; Singleton et al., 2019; Yang et al., 2015). Concomitant effects such as bark beetle infestations exerted additional pressures on forests and could explain the observed C density decline in the RM between 2007 and 2017 (Figure 2d; Anderegg et al., 2022; Hicke et al., 2016; Kolb et al., 2016). Higher tree mortality due to bark beetle outbreaks and drought stress may also be related to increased wildfires because of reduced resilience and more dead wood fuels (Hicke et al., 2013, 2020). Bark beetles and drought may have played a larger role in changes to C‐balances in western forests, compared to the removals that we specifically analysed. They are indirectly represented by negative stock change values in 1940–1950, 1960–1970, and 2007–2017 in the RM, and between 1940, 1960 and 1970–1987 in the PC (Figures 2d and 2e). Given the interrelation of all these processes, the relative effect of wildfires for the C balance might become more important in the future as climate change effects intensify (Anderegg et al., 2022; Barbero et al., 2015).

### 4.4 Implications

The US case study provides valuable insights into the drivers of forest biomass C stock changes and their interconnected dynamics, which are relevant in the context of forest‐centered climate change mitigation strategies. We argue that a better understanding of the temporal and regional mechanisms that influence C dynamics in forests is of vital importance (Gingrich et al., 2019; Loudermilk et al., 2013; Magerl et al., 2022). In a previous study (Magerl et al., 2019), we showed that contemporary biomass C stock densities in forests in the contiguous U.S. only amount to roughly 50% of what their hypothetical potential (without land‐use) would be in the East and around 70% in the West. This difference is the reason for the large “natural climate solutions” potential in the U.S. (Fargione et al., 2018). However, to which extent this potential can be realised is highly uncertain and depends on a range of interconnected drivers.

Currently, U.S. forests represent a net biomass C sink, sequestering the equivalent of around 10% of the nation's C emissions by fossil fuel combustion and cement production (Friedlingstein et al., 2022). This sink can largely be attributed to an age‐structure effect, that is, due to recovery from past disturbances through fire suppression and replanting, especially in the Southeast (Ghimire et al., 2012; Gu et al., 2016). Although we did not address legacy effects empirically in this study, the observed lower C stocks in the early 20th century in the East, as well as the rejuvenation and strong stock regrowth in this region compared to the other regions and later decades, point to the importance of past uses of forests for contemporary observed forest developments. This argumentation is in line with historical literature (e.g., MacCleery, 1993; Maxwell, 1973) and previous studies on past U.S. land use and forest change (e.g., Houghton, 1999; Raiho et al., 2022).

Despite their high productivity and contribution to net growth of forest biomass in the country, south‐eastern forests have low resilience toward wildfires, mainly due to their stand age, and structure, and thus require great fire manamanget efforts (Mitchell et al., 2014; Nowacki & Abrams, 2008; White et al., 1985). Increasing wildfires, other disturbances and harvest offset gross biomass growth in the more mature Western forests (particularly in the PC), resulting in small average net growth in this part of the country over the 20th century. With changing climate, and fuel accumulation due to past fire exclusion (Calkin et al., 2015), increases in occurrence and severity of wildfires are expected throughout the contiguous U.S. (Barbero et al., 2015). Liu et al. (2021) estimated a 50% increase in wildfire emissions until 2059 in the West, while E. K. Brown et al. (2021) simulate a nearly 60 times increase in droughts throughout the south‐eastern U.S. until the end of the 21st century that could lead to surges in burned area and more frequent and severe wildfires. These developments might be counteracting possible increases of the C‐sink function of forests; thus, the future size and persistence of the forest C sink is uncertain (Kolden, 2019; Parks et al., 2015; Wear & Coulston, 2015).

These challenges also need to be considered in the light of the expected growth of demand for forest biomass (Johnston & Radeloff, 2019; United States Environmental Protection Agency (USEPA), 2018), for example, for harvested wood products and biofuels in the context of climate change mitigation. In the past century, harvest was the largest removal of forest biomass in the contiguous U.S., although not all C in harvested wood represents an immediate emission to the atmosphere. Whether harvested wood for construction represents a C sink strongly depends on the lifecycle of products and the ultimate fate of the C they contain after the end of their lifetime (Erb et al., 2022; Gu et al., 2019). Changing demand for wood can be connected to possible problem shifts and displacement effects. Decreasing harvest under growing wood demand can lead to shifting harvest activities from one region to another or outsourcing of wood extraction to other countries via foreign trade, driving deforestation abroad (Gingrich et al., 2019; Popp et al., 2014). Harvest has varying implications for biomass C‐dynamics in forests that interact with fire dynamics, such as changing C stocks, fuel loads, biodiversity, and resilience against disturbances. The magnitudes of these implications depend on a range of factors, including intensity of harvest and treatment of forest fuels, as well as harvest residues and slash (Law et al., 2018; Pan et al., 2011).

Our study showed that the dynamics that drive forest biomass change are interconnected and characterised by heterogenous temporal and spatial dynamics. The anthropogenic fire suppression regime established over the 20th century, the recovery of forests from past harvest and transformation to commercially managed timber forests contributed to the observed recent U.S. forest C sink. Despite the rigorous suppression policies, recently, wildfires and other disturbances have increased, mainly due to changing climate. Climate change introduces a substantial uncertainty in the already complex system of forest biomass dynamics. Whether the current fire suppression regime will continue to contribute to growing biomass C stocks in U.S. forests or not is beyond the scope of our study. However, we argue that to inform future forest management and natural climate solutions, it is essential to understand the dynamics that drive biomass C stock change and their complex interplay. Legacy effects and feedback loops of the drivers of forest biomass change, as well as possible leakages and trade‐offs of protection and management measures might counteract C sink gains or limit the effectiveness of natural climate solutions. Temporal and regional long‐term dynamics need to be considered in studies addressing the potential of natural climate solutions, future forest management and protection measures. Striking a balance between maintaining the C sink function of forests, wildfire management and optimising harvesting will be major challenges to forest management.

## 5 Conclusions

In this study, we quantified the long‐term dynamics of the drivers of the biomass C balance in forests in the contiguous U.S., investigating the impact of wildfires on forest biomass in comparison to wood harvest and forest grazing. We developed a robust reconstruction of burnt biomass by combining historical statistics and satellite observations. The C balance is based on a consistent data set, harmonizing different data sources covering most of the 20th and the early 21st centuries on various spatial and temporal scales.

Net C stock gains over the past century led to the establishment of a consistent C sink in forest biomass in the U.S., although the combined effect of fire and harvest decreased net C gains in forest biomass stocks by almost 60%. The sub‐national disaggregation highlights that different drivers were responsible for observed C stock changes in each of the four study regions. Overall C gains were mostly driven by positive stock change rates in the Southeast, where gross growth outweighed gross removals. Recovery from past harvest and the establishment of a wildfire suppression regime, aimed to eliminate human‐ignited wildfires, contributed to the observed biomass regrowth. In contrast to the eastern forests, the more mature western forests showed higher and more stable C densities throughout the observed period. However, their contribution to net C gains was less significant due to highly dynamic stock change rates (especially in the Pacific Coast) that fluctuated between net gains and losses. Western forests are characterized by a more mixed forest structure, a higher proportion of disturbances such as bark beetles in the C balance, relatively intensive timber harvest in the Pacific Coast region, and less pronounced legacies of extensive harvest and land clearing, compared to the east. Despite large‐scale fire suppression efforts, rates of western wildfires have increased in recent decades, arguably due to climate change.

Wildfire suppression contributed to forest recovery with regional variance but wood harvest was the key driver in the biomass C balance of forests of the U.S. for most of the observed time‐period. Further increases of wildfires and other disturbances such as insect infestations and droughts are expected throughout the contiguous U.S. Our study highlights the importance of understanding the long‐term dynamics of the interconnected drivers of forest biomass change in the context of natural climate solutions.

## Supporting information

Supporting Information S1

## Data Availability

The data used to reconstruct the burned area and biomass as well as industrial wood and fuelwood harvest and the biomass C stock dynamics are available for free as an Excel spreadsheet (Magerl et al., 2023) (DOI: https://doi.org/10.5281/zenodo.7891946) at the following repository: https://zenodo.org/record/7891946.

## References

[gbc21453-bib-0001] Abatzoglou, J. T. , & Williams, A. P. (2016). Impact of anthropogenic climate change on wildfire across western US forests. Proceedings of the National Academy of Sciences, 113(42), 11770–11775. 10.1073/pnas.1607171113 PMC5081637 27791053

[gbc21453-bib-0002] Andela, N. , Morton, D. C. , Giglio, L. , Paugam, R. , Chen, Y. , Hantson, S. , et al. (2019). The Global Fire Atlas of individual fire size, duration, speed and direction. Earth System Science Data, 11(2), 529–552. 10.5194/essd-11-529-2019

[gbc21453-bib-0003] Anderegg, W. R. L. , Chegwidden, O. S. , Badgley, G. , Trugman, A. T. , Cullenward, D. , Abatzoglou, J. T. , et al. (2022). Future climate risks from stress, insects and fire across US forests. Ecology Letters, 25(6), 1510–1520. 10.1111/ele.14018 35546256 PMC9321543

[gbc21453-bib-0004] Archibald, S. , Lehmann, C. E. R. , Gómez‐Dans, J. L. , & Bradstock, R. A. (2013). Defining pyromes and global syndromes of fire regimes. Proceedings of the National Academy of Sciences, 110(16), 6442–6447. 10.1073/pnas.1211466110 PMC3631631 23559374

[gbc21453-bib-0005] Balch, J. K. , Bradley, B. A. , Abatzoglou, J. T. , Nagy, R. C. , Fusco, E. J. , & Mahood, A. L. (2017). Human‐started wildfires expand the fire niche across the United States. Proceedings of the National Academy of Sciences, 114(11), 2946–2951. 10.1073/pnas.1617394114 PMC5358354 28242690

[gbc21453-bib-0006] Barbero, R. , Abatzoglou, J. T. , Larkin, N. K. , Kolden, C. A. , & Stocks, B. (2015). Climate change presents increased potential for very large fires in the contiguous United States. International Journal of Wildland Fire, 24(7), 892. 10.1071/WF15083

[gbc21453-bib-0007] Birdsey, R. , Pregitzer, K. , & Lucier, A. (2006). Forest carbon management in the United States. Journal of Environmental Quality, 35(4), 1461–1469. 10.2134/jeq2005.0162 16825466

[gbc21453-bib-0008] Bowman, D. M. J. S. , Balch, J. , Artaxo, P. , Bond, W. J. , Cochrane, M. A. , D’Antonio, C. M. , et al. (2011). The human dimension of fire regimes on Earth. Journal of Biogeography, 38(12), 2223–2236. 10.1111/j.1365-2699.2011.02595.x 22279247 PMC3263421

[gbc21453-bib-0009] Bowman, D. M. J. S. , Kolden, C. A. , Abatzoglou, J. T. , Johnston, F. H. , van der Werf, G. R. , & Flannigan, M. (2020). Vegetation fires in the Anthropocene. Nature Reviews Earth & Environment, 1(10), 500–515. 10.1038/s43017-020-0085-3

[gbc21453-bib-0010] Brown, E. K. , Wang, J. , & Feng, Y. (2021). US wildfire potential: A historical view and future projection using high‐resolution climate data. Environmental Research Letters, 16(3), 034060. 10.1088/1748-9326/aba868

[gbc21453-bib-0011] Brown, T. J. , Hall, B. L. , Mohrle, C. R. , & Reinbold, H. J. (2002). Coarse assessment of federal wildland fire occurrence data. In Report for the national wildfire coordinating group, CEFA report, 02–04.

[gbc21453-bib-0012] Calkin, D. E. , Thompson, M. P. , & Finney, M. A. (2015). Negative consequences of positive feedbacks in US wildfire management. Forest Ecosystems, 2(1), 9. 10.1186/s40663-015-0033-8

[gbc21453-bib-0013] Churkina, G. , Organschi, A. , Reyer, C. P. O. , Ruff, A. , Vinke, K. , Liu, Z. , et al. (2020). Buildings as a global carbon sink. Nature Sustainability, 3(4), 269–276. 10.1038/s41893-019-0462-4

[gbc21453-bib-0014] Chuvieco, E. , Mouillot, F. , van der Werf, G. R. , San Miguel, J. , Tanase, M. , Koutsias, N. , et al. (2019). Historical background and current developments for mapping burned area from satellite Earth observation. Remote Sensing of Environment, 225, 45–64. 10.1016/j.rse.2019.02.013

[gbc21453-bib-0015] Ciesla, W. M. , & Mason, A. C. (2005). Disturbance events in America’s forests: An analysis of criterion 3, indicator 15 of the Montreal process—Criteria and indicators of sustainable forestry—2003 [analysis] (p. 89). United States Department of Agriculture, Forest Service, Forest Health Technology Enterprise Team. Retrieved from https://citeseerx.ist.psu.edu/document?repid=rep1&type=pdf&doi=104e781b2a17b7967d19d20a8e5c4eed3694354c

[gbc21453-bib-0016] Conedera, M. , Tinner, W. , Neff, C. , Meurer, M. , Dickens, A. F. , & Krebs, P. (2009). Reconstructing past fire regimes: Methods, applications, and relevance to fire management and conservation. Quaternary Science Reviews, 28(5–6), 555–576. 10.1016/j.quascirev.2008.11.005

[gbc21453-bib-0017] Courtwright, J. (2011). Prairie fire: A Great Plains history. University Press of Kansas.

[gbc21453-bib-0018] Davis, D. E. (2003). Where there are mountains: An environmental history of the southern Appalachians. University of Georgia Press.

[gbc21453-bib-0019] Dennison, P. E. , Brewer, S. C. , Arnold, J. D. , & Moritz, M. A. (2014). Large wildfire trends in the western United States, 1984–2011: Dennison et al.; large wildfire trends in the western US. Geophysical Research Letters, 41(8), 2928–2933. 10.1002/2014GL059576

[gbc21453-bib-0020] Eggleston, H. S. , Intergovernmental Panel on Climate Change , National Greenhouse Gas Inventories Programme , & Chikyū Kankyō Senryaku Kenkyū Kikan . (2006). 2006 IPCC guidelines for national greenhouse gas inventories. Retrieved from http://www.ipcc-nggip.iges.or.jp/public/2006gl/index.htm

[gbc21453-bib-0021] Erb, K.‐H. , Haberl, H. , Le Noë, J. , Tappeiner, U. , Tasser, E. , & Gingrich, S. (2022). Changes in perspective needed to forge ‘no‐regret’ forest‐based climate change mitigation strategies. GCB Bioenergy, 14(3), 246–257. 10.1111/gcbb.12921 35909989 PMC9306738

[gbc21453-bib-0022] Erb, K.‐H. , Luyssaert, S. , Meyfroidt, P. , Pongratz, J. , Don, A. , Kloster, S. , et al. (2017). Land management: Data availability and process understanding for global change studies. Global Change Biology, 23(2), 512–533. 10.1111/gcb.13443 27447350

[gbc21453-bib-0023] Escuin, S. , Navarro, R. , & Fernández, P. (2008). Fire severity assessment by using NBR (normalized burn ratio) and NDVI (normalized difference vegetation index) derived from LANDSAT TM/ETM images. International Journal of Remote Sensing, 29(4), 1053–1073. 10.1080/01431160701281072

[gbc21453-bib-0024] FAO . (2020). FAOSTAT statistical database. Retrieved from http://www.fao.org/faostat/en/#home

[gbc21453-bib-0025] Fargione, J. E. , Bassett, S. , Boucher, T. , Bridgham, S. D. , Conant, R. T. , Cook‐Patton, S. C. , et al. (2018). Natural climate solutions for the United States. Science Advances, 4(11), eaat1869. 10.1126/sciadv.aat1869 30443593 PMC6235523

[gbc21453-bib-0026] Favero, A. , Daigneault, A. , & Sohngen, B. (2020). Forests: Carbon sequestration, biomass energy, or both? Science Advances, 6(13), eaay6792. 10.1126/sciadv.aay6792 32232153 PMC7096156

[gbc21453-bib-0027] Fedkiw, J. (1989). The evolving use and management of the Nation’s forests, grasslands, croplands, and related resources. A technical document supporting the 1989 USDA forest service RPA assessment (Vol. 175). US Department of Agriculture, Forest Service, Rocky Mountain Forest and Range Experiment Station.

[gbc21453-bib-0028] Flannigan, M. D. , Krawchuk, M. A. , de Groot, W. J. , Wotton, B. M. , & Gowman, L. M. (2009). Implications of changing climate for global wildland fire. International Journal of Wildland Fire, 18(5), 483. 10.1071/WF08187

[gbc21453-bib-0029] Ford, A. E. S. , Harrison, S. P. , Kountouris, Y. , Millington, J. D. A. , Mistry, J. , Perkins, O. , et al. (2021). Modelling human‐fire interactions: Combining alternative perspectives and approaches. Frontiers in Environmental Science, 9, 649835. 10.3389/fenvs.2021.649835

[gbc21453-bib-0030] Fowler, C. , & Konopik, E. (2007). The history of fire in the southern United States. Human Ecology Review, 14(2), 165–176. JSTOR.

[gbc21453-bib-0031] French, N. H. F. , McKenzie, D. , Erickson, T. , Koziol, B. , Billmire, M. , Endsley, K. A. , et al. (2014). Modeling regional‐scale wildland fire emissions with the wildland fire emissions information system. Earth Interactions, 18(16), 1–26. 10.1175/EI-D-14-0002.1

[gbc21453-bib-0032] Friedlingstein, P. , O’Sullivan, M. , Jones, M. W. , Andrew, R. M. , Gregor, L. , Hauck, J. , et al. (2022). Global carbon budget 2022. Earth System Science Data, 14(11), 4811–4900. 10.5194/essd-14-4811-2022

[gbc21453-bib-0033] Frost, E. J. , & Sweeney, R. (2000). Fire regimes, fire history and forest conditions in the Klamath‐Siskiyou Region: An overview and synthesis of knowledge. In World Wildlife Fund, Klamath‐Siskiyou ecoregion program, Ashland, OR.

[gbc21453-bib-0034] Gedalof, Z. , Peterson, D. L. , & Mantua, N. J. (2005). Atmospheric, climatic, and ecological controls on extreme wildfire years in the northwestern United States. Ecological Applications, 15(1), 154–174. 10.1890/03-5116

[gbc21453-bib-0035] Geng, A. , Yang, H. , Chen, J. , & Hong, Y. (2017). Review of carbon storage function of harvested wood products and the potential of wood substitution in greenhouse gas mitigation. Forest Policy and Economics, 85, 192–200. 10.1016/j.forpol.2017.08.007

[gbc21453-bib-0036] Ghimire, B. , Williams, C. A. , Collatz, G. J. , & Vanderhoof, M. (2012). Fire‐induced carbon emissions and regrowth uptake in western U.S. forests: Documenting variation across forest types, fire severity, and climate regions: FIRE‐INDUCED carbon emissions and regrowth uptake. Journal of Geophysical Research, 117(G3), G03036. 10.1029/2011JG001935

[gbc21453-bib-0037] Gierlinger, S. , & Krausmann, F. (2012). The physical economy of the United States of America: Extraction, trade, and consumption of materials from 1870 to 2005. Journal of Industrial Ecology, 16(3), 365–377. 10.1111/j.1530-9290.2011.00404.x 24436632 PMC3886303

[gbc21453-bib-0038] Gingrich, S. , Lauk, C. , Niedertscheider, M. , Pichler, M. , Schaffartzik, A. , Schmid, M. , et al. (2019). Hidden emissions of forest transitions: A socio‐ecological reading of forest change. Current Opinion in Environmental Sustainability, 38, 14–21. 10.1016/j.cosust.2019.04.005

[gbc21453-bib-0039] Gingrich, S. , Magerl, A. , Matej, S. , & Le Noë, J. (2022). Forest transitions in the United States, France and Austria: Dynamics of forest change and their socio‐ metabolic drivers. Journal of Land Use Science, 17, 1–21. 10.1080/1747423X.2021.2018514 PMC9038175 35492807

[gbc21453-bib-0040] Grala, K. , & Cooke, W. H. (2010). Spatial and temporal characteristics of wildfires in Mississippi, USA. International Journal of Wildland Fire, 19(1), 14. 10.1071/WF08104

[gbc21453-bib-0041] Gregg, S. M. (2010). Managing the mountains: Land use planning, the new deal, and the creation of a federal landscape in Appalachia. Yale University Press; JSTOR. Retrieved from https://www.jstor.org/stable/j.ctt1np994

[gbc21453-bib-0042] Grelen, H. E. (1978). Forest grazing in the south. Rangeland Ecology & Management/Journal of Range Management Archives, 31(4), 244–250. 10.2307/3897592

[gbc21453-bib-0043] Gu, H. , Williams, C. A. , Ghimire, B. , Zhao, F. , & Huang, C. (2016). High‐resolution mapping of time since disturbance and forest carbon flux from remote sensing and inventory data to assess harvest, fire, and beetle disturbance legacies in the Pacific Northwest. Biogeosciences, 13(22), 6321–6337. 10.5194/bg-13-6321-2016

[gbc21453-bib-0044] Gu, H. , Williams, C. A. , Hasler, N. , & Zhou, Y. (2019). The carbon balance of the southeastern U.S. Forest sector as driven by recent disturbance trends. Journal of Geophysical Research: Biogeosciences, 124(9), 2786–2803. 10.1029/2018JG004841

[gbc21453-bib-0045] Guyette, R. P. , Dey, D. C. , & Stambaugh, M. C. (2003). Fire and human history of a barren‐forest mosaic in southern Indiana. The American Midland Naturalist, 149(1), 21–34. 10.1674/0003-0031(2003)149[0021:fahhoa]2.0.co;2

[gbc21453-bib-0046] Hansbrough, T. (1961). A sociological analysis of man‐caused forest fires in Louisiana. Louisiana State University and Agricultural & Mechanical College.

[gbc21453-bib-0047] Harris, N. L. , Hagen, S. C. , Saatchi, S. S. , Pearson, T. R. H. , Woodall, C. W. , Domke, G. M. , et al. (2016). Attribution of net carbon change by disturbance type across forest lands of the conterminous United States. Carbon Balance and Management, 11(1), 24. 10.1186/s13021-016-0066-5 27909460 PMC5108824

[gbc21453-bib-0048] Hart, J. F. (1977). Land rotation in Appalachia. Geographical Review, 67(2), 148–166. 10.2307/214017

[gbc21453-bib-0049] Haugo, R. D. , Hall, S. A. , Gray, E. M. , Gonzalez, P. , & Bakker, J. D. (2010). Influences of climate, fire, grazing, and logging on woody species composition along an elevation gradient in the eastern Cascades, Washington. Forest Ecology and Management, 260(12), 2204–2213. 10.1016/j.foreco.2010.09.021

[gbc21453-bib-0050] Haverd, V. , Smith, B. , Canadell, J. G. , Cuntz, M. , Mikaloff‐Fletcher, S. , Farquhar, G. , et al. (2020). Higher than expected CO_2_ fertilization inferred from leaf to global observations. Global Change Biology, 26(4), 2390–2402. 10.1111/gcb.14950 32017317 PMC7154678

[gbc21453-bib-0051] Hawbaker, T. J. , Radeloff, V. C. , Stewart, S. I. , Hammer, R. B. , Keuler, N. S. , & Clayton, M. K. (2013). Human and biophysical influences on fire occurrence in the United States. Ecological Applications, 23(3), 565–582. 10.1890/12-1816.1 23734486

[gbc21453-bib-0052] Hawbaker, T. J. , Vanderhoof, M. K. , Beal, Y.‐J. , Takacs, J. D. , Schmidt, G. L. , Falgout, J. T. , et al. (2017). Mapping burned areas using dense time‐series of Landsat data. Remote Sensing of Environment, 198, 504–522. 10.1016/j.rse.2017.06.027

[gbc21453-bib-0053] Hawbaker, T. J. , Vanderhoof, M. K. , Schmidt, G. L. , Beal, Y.‐J. , Picotte, J. J. , Takacs, J. D. , et al. (2020). The Landsat Burned Area algorithm and products for the conterminous United States. Remote Sensing of Environment, 244, 111801. 10.1016/j.rse.2020.111801

[gbc21453-bib-0054] Hessburg, P. F. , & Agee, J. K. (2003). An environmental narrative of Inland Northwest United States forests, 1800–2000. Forest Ecology and Management, 178(1–2), 23–59. 10.1016/S0378-1127(03)00052-5

[gbc21453-bib-0055] Hicke, J. A. , Meddens, A. J. H. , Allen, C. D. , & Kolden, C. A. (2013). Carbon stocks of trees killed by bark beetles and wildfire in the western United States. Environmental Research Letters, 8(3), 035032. 10.1088/1748-9326/8/3/035032

[gbc21453-bib-0056] Hicke, J. A. , Meddens, A. J. H. , & Kolden, C. A. (2016). Recent tree mortality in the western United States from bark beetles and forest fires. Forest Science, 62(2), 141–153. 10.5849/forsci.15-086

[gbc21453-bib-0057] Hicke, J. A. , Xu, B. , Meddens, A. J. H. , & Egan, J. M. (2020). Characterizing recent bark beetle‐caused tree mortality in the western United States from aerial surveys. Forest Ecology and Management, 475, 118402. 10.1016/j.foreco.2020.118402

[gbc21453-bib-0058] Higuera, P. E. , Abatzoglou, J. T. , Littell, J. S. , & Morgan, P. (2015). The changing strength and nature of fire‐climate relationships in the northern Rocky Mountains, U.S.A., 1902–2008. PLoS One, 10(6), e0127563. 10.1371/journal.pone.0127563 26114580 PMC4482589

[gbc21453-bib-0059] Homer, C. H. , Fry, J. A. , & Barnes, C. A. (2012). The national land cover database. US Geological Survey Fact Sheet, 3020(4), 1–4.

[gbc21453-bib-0060] Houghton, R. A. (1999). The annual net flux of carbon to the atmosphere from changes in land use 1850–1990. Tellus B: Chemical and Physical Meteorology, 51(2), 298–313. 10.1034/j.1600-0889.1999.00013.x

[gbc21453-bib-0061] Houghton, R. A. , & Hackler, J. L. (2000). Changes in terrestrial carbon storage in the United States. 1: The roles of agriculture and forestry. Global Ecology and Biogeography, 9(2), 125–144. 10.1046/j.1365-2699.2000.00166.x

[gbc21453-bib-0062] Houghton, R. A. , Hackler, J. L. , & Lawrence, K. T. (2000). Changes in terrestrial carbon storage in the United States. 2: The role of fire and fire management. Global Ecology and Biogeography, 9(2), 145–170. 10.1046/j.1365-2699.2000.00164.x

[gbc21453-bib-0063] Hurteau, M. D. , & Brooks, M. L. (2011). Short‐and long‐term effects of fire on carbon in US dry temperate forest systems. BioScience, 61(2), 139–146. 10.1525/bio.2011.61.2.9

[gbc21453-bib-0064] Hurtt, G. C. , Pacala, S. W. , Moorcroft, P. R. , Caspersen, J. , Shevliakova, E. , Houghton, R. A. , & Moore, B. (2002). Projecting the future of the U.S. carbon sink. Proceedings of the National Academy of Sciences, 99(3), 1389–1394. 10.1073/pnas.012249999 PMC122200 11830663

[gbc21453-bib-0065] Iriarte‐Goñi, I. , & Ayuda, M.‐I. (2018). Should Forest Transition Theory include effects on forest fires? The case of Spain in the second half of the twentieth century. Land Use Policy, 76, 789–797. 10.1016/j.landusepol.2018.03.009

[gbc21453-bib-0066] Ito, A. (2004). Global estimates of biomass burning emissions based on satellite imagery for the year 2000. Journal of Geophysical Research, 109(D14), D14S05. 10.1029/2003JD004423

[gbc21453-bib-0067] Johnston, C. M. T. , & Radeloff, V. C. (2019). Global mitigation potential of carbon stored in harvested wood products. Proceedings of the National Academy of Sciences, 116(29), 14526–14531. 10.1073/pnas.1904231116 PMC6642351 31262824

[gbc21453-bib-0068] Jurgelski, W. M. (2008). Burning seasons, burning bans: Fire in the southern Appalachian mountains, 1750–2000. Appalachian Journal, 35(3), 170–217.

[gbc21453-bib-0069] Kastner, T. , Matej, S. , Forrest, M. , Gingrich, S. , Haberl, H. , Hickler, T. , et al. (2022). Land use intensification increasingly drives the spatiotemporal patterns of the global human appropriation of net primary production in the last century. Global Change Biology, 28(1), 307–322. 10.1111/gcb.15932 34651392

[gbc21453-bib-0070] Kelly, L. T. , Giljohann, K. M. , Duane, A. , Aquilué, N. , Archibald, S. , Batllori, E. , et al. (2020). Fire and biodiversity in the Anthropocene. Science, 370(6519), eabb0355. 10.1126/science.abb0355 33214246

[gbc21453-bib-0071] Keywood, M. , Kanakidou, M. , Stohl, A. , Dentener, F. , Grassi, G. , Meyer, C. P. , et al. (2013). Fire in the air: Biomass burning impacts in a changing climate. Critical Reviews in Environmental Science and Technology, 43(1), 40–83. 10.1080/10643389.2011.604248

[gbc21453-bib-0072] Kolb, T. E. , Fettig, C. J. , Ayres, M. P. , Bentz, B. J. , Hicke, J. A. , Mathiasen, R. , et al. (2016). Observed and anticipated impacts of drought on forest insects and diseases in the United States. Forest Ecology and Management, 380, 321–334. 10.1016/j.foreco.2016.04.051

[gbc21453-bib-0073] Kolden, C. (2019). We’re not doing enough prescribed fire in the western United States to mitigate wildfire risk. Fire, 2(2), 30. 10.3390/fire2020030

[gbc21453-bib-0074] Krausmann, F. , Erb, K.‐H. , Gingrich, S. , Haberl, H. , Bondeau, A. , Gaube, V. , et al. (2013). Global human appropriation of net primary production doubled in the 20th century. Proceedings of the National Academy of Sciences, 110(25), 10324–10329. 10.1073/pnas.1211349110 PMC3690849 23733940

[gbc21453-bib-0075] Krebs, P. , Pezzatti, G. B. , Mazzoleni, S. , Talbot, L. M. , & Conedera, M. (2010). Fire regime: History and definition of a key concept in disturbance ecology. Theory in Biosciences, 129(1), 53–69. 10.1007/s12064-010-0082-z 20502984

[gbc21453-bib-0076] Lake, F. K. , Wright, V. , Morgan, P. , McFadzen, M. , McWethy, D. , & Stevens‐Rumann, C. (2017). Returning fire to the land: Celebrating traditional knowledge and fire. Journal of Forestry, 115(5), 343–353. 10.5849/jof.2016-043R2

[gbc21453-bib-0077] Lauk, C. , & Erb, K. H. (2016). A burning issue: Anthropogenic vegetation fires. In H. Haberl , M. Fischer‐Kowalski , F. Krausmann , & V. Winiwarter (Eds.), Social ecology. Society‐nature relations across time and Space (Vol. 5, pp. 335–348). Springer International Publishing. 10.1007/978-3-319-33326-7_15

[gbc21453-bib-0078] Law, B. E. , Hudiburg, T. W. , Berner, L. T. , Kent, J. J. , Buotte, P. C. , & Harmon, M. E. (2018). Land use strategies to mitigate climate change in carbon dense temperate forests. Proceedings of the National Academy of Sciences, 115(14), 3663–3668. 10.1073/pnas.1720064115 PMC5889652 29555758

[gbc21453-bib-0079] Leenhouts, B. (1998). Assessment of biomass burning in the conterminous United States. Conservation Ecology, 2(1), art1. JSTOR. 10.5751/es-00035-020101

[gbc21453-bib-0080] Liebmann, M. J. , Farella, J. , Roos, C. I. , Stack, A. , Martini, S. , & Swetnam, T. W. (2016). Native American depopulation, reforestation, and fire regimes in the Southwest United States, 1492–1900 CE. Proceedings of the National Academy of Sciences, 113(6), E696–E704. 10.1073/pnas.1521744113 PMC4760783 26811459

[gbc21453-bib-0081] Littell, J. S. , McKenzie, D. , Peterson, D. L. , & Westerling, A. L. (2009). Climate and wildfire area burned in western U.S. ecoprovinces, 1916–2003. Ecological Applications, 19(4), 1003–1021. 10.1890/07-1183.1 19544740

[gbc21453-bib-0082] Littell, J. S. , Oneil, E. E. , McKenzie, D. , Hicke, J. A. , Lutz, J. A. , Norheim, R. A. , & Elsner, M. M. (2010). Forest ecosystems, disturbance, and climatic change in Washington State, USA. Climatic Change, 102(1–2), 129–158. 10.1007/s10584-010-9858-x

[gbc21453-bib-0083] Liu, Y. , Liu, Y. , Fu, J. , Yang, C.‐E. , Dong, X. , Tian, H. , et al. (2021). Projection of future wildfire emissions in western USA under climate change: Contributions from changes in wildfire, fuel loading and fuel moisture. International Journal of Wildland Fire, 31(1), 1–13. 10.1071/WF20190

[gbc21453-bib-0084] Loehle, C. (2020). Historical forest changes in the western United States. The Forestry Chronicle, 96(1), 36–49. 10.5558/tfc2020-006

[gbc21453-bib-0085] Loudermilk, E. L. , Scheller, R. M. , Weisberg, P. J. , Yang, J. , Dilts, T. E. , Karam, S. L. , & Skinner, C. (2013). Carbon dynamics in the future forest: The importance of long‐term successional legacy and climate‐fire interactions. Global Change Biology, 19(11), 3502–3515. 10.1111/gcb.12310 23821586

[gbc21453-bib-0086] MacCleery, D. W. (1993). American forests: A history of a resiliency and recovery (slightly rev). U.S. Department of Agriculture, Forest Service in cooperation with Forest History Society.

[gbc21453-bib-0087] Magerl, A. , Gingrich, S. , Matej, S. , Cunfer, G. , Forrest, M. , Lauk, C. , et al. (2023). The role of wildfires in the interplay of forest carbon stocks and wood harvest in the contiguous United States during the 20th century (Version 1) [Dataset]. Zenodo. 10.5281/ZENODO.7891946 PMC10909529 38439941

[gbc21453-bib-0088] Magerl, A. , Le Noë, J. , Erb, K.‐H. , Bhan, M. , & Gingrich, S. (2019). A comprehensive data‐based assessment of forest ecosystem carbon stocks in the US 1907–2012. Environmental Research Letters, 14(12), 125015. 10.1088/1748-9326/ab5cb6

[gbc21453-bib-0089] Magerl, A. , Matej, S. , Kaufmann, L. , Noë, J. L. , Erb, K. , & Gingrich, S. (2022). Forest carbon sink in the U.S. (1870–2012) driven by substitution of forest ecosystem service flows. Resources, Conservation and Recycling, 176, 105927. 10.1016/j.resconrec.2021.105927

[gbc21453-bib-0090] Malamud, B. D. , Millington, J. D. A. , & Perry, G. L. W. (2005). Characterizing wildfire regimes in the United States. Proceedings of the National Academy of Sciences, 102(13), 4694–4699. 10.1073/pnas.0500880102 PMC555719 15781868

[gbc21453-bib-0091] Mantgem, P. J. V. , Stephenson, N. L. , Byrne, J. C. , Daniels, L. D. , Franklin, J. F. , Fulé, P. Z. , et al. (2009). Widespread increase of tree mortality rates in the western United States. Science, 323(5913), 521–524. 10.1126/science.1165000 19164752

[gbc21453-bib-0092] Marlon, J. R. , Bartlein, P. J. , Daniau, A.‐L. , Harrison, S. P. , Maezumi, S. Y. , Power, M. J. , et al. (2013). Global biomass burning: A synthesis and review of Holocene paleofire records and their controls. Quaternary Science Reviews, 65, 5–25. 10.1016/j.quascirev.2012.11.029

[gbc21453-bib-0093] Marlon, J. R. , Bartlein, P. J. , Gavin, D. G. , Long, C. J. , Anderson, R. S. , Briles, C. E. , et al. (2012). Long‐term perspective on wildfires in the western USA. Proceedings of the National Academy of Sciences, 109(9), E535–E543. 10.1073/pnas.1112839109 PMC3295264 22334650

[gbc21453-bib-0094] Mather, A. S. (1992). The forest transition. Area, 24(4), 367–379.

[gbc21453-bib-0095] Maxwell, R. S. (1973). The impact of forestry on the Gulf South. Forest History Newsletter, 17(1), 30–35. 10.2307/4004192

[gbc21453-bib-0096] Mishra, A. , Humpenöder, F. , Churkina, G. , Reyer, C. P. O. , Beier, F. , Bodirsky, B. L. , et al. (2022). Land use change and carbon emissions of a transformation to timber cities. Nature Communications, 13(1), 4889. 10.1038/s41467-022-32244-w PMC9427734 36042197

[gbc21453-bib-0097] Mitchell, R. J. , Liu, Y. , O’Brien, J. J. , Elliott, K. J. , Starr, G. , Miniat, C. F. , & Hiers, J. K. (2014). Future climate and fire interactions in the southeastern region of the United States. Forest Ecology and Management, 327, 316–326. 10.1016/j.foreco.2013.12.003

[gbc21453-bib-0098] Moritz, M. A. , Parisien, M.‐A. , Batllori, E. , Krawchuk, M. A. , Van Dorn, J. , Ganz, D. J. , & Hayhoe, K. (2012). Climate change and disruptions to global fire activity. Ecosphere, 3(6), art49. 10.1890/ES11-00345.1

[gbc21453-bib-0099] National Interagency Fire Center . (2020). Total wildland fires and acres (1926–2019) [Dataset]. National Interagency Coordination Center. Retrieved from https://www.nifc.gov/fireInfo/fireInfo_stats_totalFires.html

[gbc21453-bib-0100] Nowacki, G. J. , & Abrams, M. D. (2008). The demise of fire and “mesophication” of forests in the eastern United States. BioScience, 58(2), 123–138. 10.1641/B580207

[gbc21453-bib-0101] O’Connor, C. D. , Garfin, G. M. , Falk, D. A. , & Swetnam, T. W. (2011). Human pyrogeography: A new synergy of fire, climate and people is reshaping ecosystems across the globe: Human pyrogeography. Geography Compass, 5(6), 329–350. 10.1111/j.1749-8198.2011.00428.x

[gbc21453-bib-0102] Oswalt, S. N. , Miles, P. D. , Pugh, S. A. , & Smith, W. B. (2018). Forest resources of the United States, 2017: A technical document supporting the forest service 2020 update of the RPA assessment (resources planning act (RPA) assessment) [forest resource statistics] (p. 146). U.S. Department of Agriculture, Forest Service.

[gbc21453-bib-0103] Oswalt, S. N. , Smith, W. B. , Miles, P. D. , & Pugh, S. A. (2014). Forest resources of the United States, 2012: A technical document supporting the forest service 2010 update of the RPA assessment (WO‐GTR‐91). U.S. Department of Agriculture, Forest Service. 10.2737/WO-GTR-91

[gbc21453-bib-0104] Otto, J. S. (1983). The decline of forest farming in southern Appalachia. Journal of Forest History, 27(1), 18–27. 10.2307/4004858

[gbc21453-bib-0105] Otto, J. S. , & Anderson, N. (1982). Slash‐and‐burn cultivation in the Highlands South: A problem in comparative agricultural history. Comparative Studies in Society and History, 24(1), 131–147. 10.1017/s0010417500009816

[gbc21453-bib-0106] Pan, Y. , Chen, J. M. , Birdsey, R. , McCullough, K. , He, L. , & Deng, F. (2011). Age structure and disturbance legacy of North American forests. Biogeosciences, 8(3), 715–732. 10.5194/bg-8-715-2011

[gbc21453-bib-0107] Parisien, M.‐A. , Miller, C. , Parks, S. A. , DeLancey, E. R. , Robinne, F.‐N. , & Flannigan, M. D. (2016). The spatially varying influence of humans on fire probability in North America. Environmental Research Letters, 11(7), 075005. 10.1088/1748-9326/11/7/075005

[gbc21453-bib-0108] Parks, S. A. , Miller, C. , Parisien, M.‐A. , Holsinger, L. M. , Dobrowski, S. Z. , & Abatzoglou, J. (2015). Wildland fire deficit and surplus in the western United States, 1984–2012. Ecosphere, 6(12), art275. 10.1890/ES15-00294.1

[gbc21453-bib-0109] Park Williams, A. , Allen, C. D. , Macalady, A. K. , Griffin, D. , Woodhouse, C. A. , Meko, D. M. , et al. (2013). Temperature as a potent driver of regional forest drought stress and tree mortality. Nature Climate Change, 3(3), 292–297. 10.1038/nclimate1693

[gbc21453-bib-0110] Parton, W. J. , Gutmann, M. P. , Merchant, E. R. , Hartman, M. D. , Adler, P. R. , McNeal, F. M. , & Lutz, S. M. (2015). Measuring and mitigating agricultural greenhouse gas production in the US Great Plains, 1870–2000. Proceedings of the National Academy of Sciences, 112(34), E4681–E4688. 10.1073/pnas.1416499112 PMC4553787 26240366

[gbc21453-bib-0111] Pausas, J. G. , & Keeley, J. E. (2009). A burning story: The role of fire in the history of life. BioScience, 59(7), 593–601. 10.1525/bio.2009.59.7.10

[gbc21453-bib-0112] Pausas, J. G. , & Ribeiro, E. (2017). Fire and plant diversity at the global scale: PAUSAS and RIBEIRO. Global Ecology and Biogeography, 26(8), 889–897. 10.1111/geb.12596

[gbc21453-bib-0113] Pechony, O. , & Shindell, D. T. (2010). Driving forces of global wildfires over the past millennium and the forthcoming century. Proceedings of the National Academy of Sciences, 107(45), 19167–19170. 10.1073/pnas.1003669107 PMC2984177 20974914

[gbc21453-bib-0114] Popp, A. , Humpenöder, F. , Weindl, I. , Bodirsky, B. L. , Bonsch, M. , Lotze‐Campen, H. , et al. (2014). Land‐use protection for climate change mitigation. Nature Climate Change, 4(12), 1095–1098. 10.1038/nclimate2444

[gbc21453-bib-0115] Pugh, T. A. M. , Lindeskog, M. , Smith, B. , Poulter, B. , Arneth, A. , Haverd, V. , & Calle, L. (2019). Role of forest regrowth in global carbon sink dynamics. Proceedings of the National Academy of Sciences, 116(10), 4382–4387. 10.1073/pnas.1810512116 PMC6410874 30782807

[gbc21453-bib-0116] Pyne, S. J. (1982). Fire in America: A cultural history of wildland and rural fire (reprint edition). University of Washington Press.

[gbc21453-bib-0117] Pyne, S. J. (1991). Burning bush: A fire history of Australia. Holt.

[gbc21453-bib-0118] Pyne, S. J. (1995). World fire: The culture of fire on Earth. Holt.

[gbc21453-bib-0119] Pyne, S. J. (1997). Vestal fire: An environmental history, told through fire, of Europe and Europe's encounter with the world. University of Washington Press.

[gbc21453-bib-0120] Pyne, S. J. (2019). Fire: A brief history. University of Washington Press.

[gbc21453-bib-0121] Rabin, S. S. , Melton, J. R. , Lasslop, G. , Bachelet, D. , Forrest, M. , Hantson, S. , et al. (2017). The Fire Modeling Intercomparison Project (FireMIP), phase 1: Experimental and analytical protocols with detailed model descriptions. Geoscientific Model Development, 10(3), 1175–1197. 10.5194/gmd-10-1175-2017

[gbc21453-bib-0122] Raiho, A. M. , Paciorek, C. J. , Dawson, A. , Jackson, S. T. , Mladenoff, D. J. , Williams, J. W. , & McLachlan, J. S. (2022). 8000‐year doubling of Midwestern forest biomass driven by population‐ and biome‐scale processes. Science, 376(6600), 1491–1495. 10.1126/science.abk3126

[gbc21453-bib-0123] Reid, J. S. , Koppmann, R. , Eck, T. F. , & Eleuterio, D. P. (2005). A review of biomass burning emissions part II: Intensive physical properties of biomass burning particles. Atmospheric Chemistry and Physics, 5(3), 799–825. 10.5194/acp-5-799-2005

[gbc21453-bib-0124] Reynolds, R. V. , & Pierson, A. H. (1941). The saw‐timber resource of the United States, 1630–1930 (p. 31). United States Department of Agriculture.

[gbc21453-bib-0125] Robinson, J. M. (1989). On uncertainty in the computation of global emissions from biomass burning. Climatic Change, 14(3), 243–261. 10.1007/BF00134965

[gbc21453-bib-0126] Röder, M. , Thiffault, E. , Martínez‐Alonso, C. , Senez‐Gagnon, F. , Paradis, L. , & Thornley, P. (2019). Understanding the timing and variation of greenhouse gas emissions of forest bioenergy systems. Biomass and Bioenergy, 121, 99–114. 10.1016/j.biombioe.2018.12.019

[gbc21453-bib-0127] Rogers, B. M. , Balch, J. K. , Goetz, S. J. , Lehmann, C. E. R. , & Turetsky, M. (2020). Focus on changing fire regimes: Interactions with climate, ecosystems, and society. Environmental Research Letters, 15(3), 030201. 10.1088/1748-9326/ab6d3a

[gbc21453-bib-0128] Ruefenacht, B. , Finco, M. V. , Nelson, M. D. , Czaplewski, R. , Helmer, E. H. , Blackard, J. A. , et al. (2008). Conterminous U.S. and Alaska forest type mapping using forest inventory and analysis data. Photogrammetric Engineering & Remote Sensing, 74(11), 1379–1388. 10.14358/PERS.74.11.1379

[gbc21453-bib-0129] Schlesinger, W. H. (1997). Biogeochemistry: An analysis of global changes (p. 588).

[gbc21453-bib-0130] Schulze, E. D. , Valentini, R. , & Bouriaud, O. (2021). The role of net ecosystem productivity and of inventories in climate change research: The need for “net ecosystem productivity with harvest”. NEPH. Forest Ecosystems, 8(1), 15. 10.1186/s40663-021-00294-z

[gbc21453-bib-0131] Short, K. C. (2015). Sources and implications of bias and uncertainty in a century of US wildfire activity data. International Journal of Wildland Fire, 24(7), 883. 10.1071/WF14190

[gbc21453-bib-0132] Singleton, M. P. , Thode, A. E. , Sánchez Meador, A. J. , & Iniguez, J. M. (2019). Increasing trends in high‐severity fire in the southwestern USA from 1984 to 2015. Forest Ecology and Management, 433, 709–719. 10.1016/j.foreco.2018.11.039

[gbc21453-bib-0133] Sleeter, B. M. , Frid, L. , Rayfield, B. , Daniel, C. , Zhu, Z. , & Marvin, D. C. (2022). Operational assessment tool for forest carbon dynamics for the United States: A new spatially explicit approach linking the LUCAS and CBM‐CFS3 models. Carbon Balance and Management, 17(1), 1. 10.1186/s13021-022-00201-1 35107646 PMC8811977

[gbc21453-bib-0134] Smith, B. , Wårlind, D. , Arneth, A. , Hickler, T. , Leadley, P. , Siltberg, J. , & Zaehle, S. (2014). Implications of incorporating N cycling and N limitations on primary production in an individual‐based dynamic vegetation model. Biogeosciences, 11(7), 2027–2054. 10.5194/bg-11-2027-2014

[gbc21453-bib-0135] Song, C. , & Woodcock, C. E. (2003). A regional forest ecosystem carbon budget model: Impacts of forest age structure and landuse history. Ecological Modelling, 164(1), 33–47. 10.1016/S0304-3800(03)00013-9

[gbc21453-bib-0136] Steen, H. K. (2004). The U.S. forest service: A history. Forest History Society in Association with University of Washington Press.

[gbc21453-bib-0137] Stenzel, J. E. , Bartowitz, K. J. , Hartman, M. D. , Lutz, J. A. , Kolden, C. A. , Smith, A. M. S. , et al. (2019). Fixing a snag in carbon emissions estimates from wildfires. Global Change Biology, 25(11), 3985–3994. 10.1111/gcb.14716 31148284

[gbc21453-bib-0138] Syphard, A. D. , & Keeley, J. E. (2016). Historical reconstructions of California wildfires vary by data source. International Journal of Wildland Fire, 25(12), 1221. 10.1071/WF16050

[gbc21453-bib-0139] Teckentrup, L. , Harrison, S. P. , Hantson, S. , Heil, A. , Melton, J. R. , Forrest, M. , et al. (2019). Response of simulated burned area to historical changes in environmental and anthropogenic factors: A comparison of seven fire models. Biogeosciences, 16(19), 3883–3910. 10.5194/bg-16-3883-2019

[gbc21453-bib-0140] Thonicke, K. , Spessa, A. , Prentice, I. C. , Harrison, S. P. , Dong, L. , & Carmona‐Moreno, C. (2010). The influence of vegetation, fire spread and fire behaviour on biomass burning and trace gas emissions: Results from a process‐based model. Biogeosciences, 7(6), 1991–2011. 10.5194/bg-7-1991-2010

[gbc21453-bib-0141] Tom, E. , Adams, M. M. , & Goode, R. W. (2023). Solastalgia to Soliphilia: Cultural fire, climate change, and indigenous healing. Ecopsychology. 10.1089/eco.2022.0085

[gbc21453-bib-0142] United States Bureau of the Census . (1975). Historical statistics of the United States, colonial times to 1970. US Department of Commerce, Bureau of the Census.

[gbc21453-bib-0143] United States Environmental Protection Agency (USEPA) . (2018). EPA’s treatment of biogenic carbon dioxide (CO_2_) emissions from stationary sources that use forest biomass for energy production. Retrieved from https://epa.gov/sites/production/files/2018-04/documents/biomass_policy_statement_2018_04_23.pdf

[gbc21453-bib-0144] United States Forest Service . (1941). Forest fire statistics 1941 (Statistical report T‐62; forest fire statistics) (p. 12). United States Department of Agriculture; University of Florida.

[gbc21453-bib-0145] United States Forest Service . (1942). Forest fire statistics 1942 (Statistical report T‐65; forest fire statistics) (p. 12). United States Department of Agriculture; University of Florida.

[gbc21453-bib-0146] United States Forest Service . (1945). Forest fire statistics 1945 (forest fire statistics) [Statistical report] (p. 12). United States Department of Agriculture; University of Florida.

[gbc21453-bib-0147] United States Forest Service . (1946). Forest fire statistics 1946 (forest fire statistics) [Statistical report] (p. 10). United States Department of Agriculture; University of Florida.

[gbc21453-bib-0148] United States Forest Service . (1947). Forest fire statistics 1947 (forest fire statistics) [Statistical report] (p. 12). United States Department of Agriculture; University of Florida.

[gbc21453-bib-0149] United States Forest Service . (1948). Forest fire statistics 1948 (forest fire statistics) [Statistical report] (p. 15). United States Department of Agriculture; University of Florida.

[gbc21453-bib-0150] United States Forest Service . (1949). Forest fire statistics 1949 (forest fire statistics) [Statistical report] (p. 16). United States Department of Agriculture; University of Florida.

[gbc21453-bib-0151] United States Forest Service . (1950). Forest fire statistics 1950 (forest fire statistics) [Statistical report] (p. 15). United States Department of Agriculture; University of Florida.

[gbc21453-bib-0152] United States Forest Service . (1951). Forest fire statistics 1951 (forest fire statistics) [Statistical report] (p. 15). United States Department of Agriculture; University of Florida.

[gbc21453-bib-0153] United States Forest Service . (1954). Forest fire statistics 1954 (forest fire statistics) [Statistical report] (p. 14). United States Department of Agriculture; University of Florida.

[gbc21453-bib-0154] United States Forest Service . (1955). Forest fire statistics 1955 (forest fire statistics) [Statistical report] (p. 13). United States Department of Agriculture; University of Florida.

[gbc21453-bib-0155] United States Forest Service . (1956). Forest fire statistics 1956 (forest fire statistics) [Statistical report] (p. 12). United States Department of Agriculture; University of Florida.

[gbc21453-bib-0156] United States Forest Service . (1958). Forest fire statistics 1958 (forest fire statistics) [Statistical report] (p. 14). United States Department of Agriculture; University of Florida.

[gbc21453-bib-0157] United States Forest Service . (1960). Annual fire report for the National forest (p. 22). United States Department of Agriculture; National Agricultural Library.

[gbc21453-bib-0158] United States Forest Service . (1961). Annual fire report for the National forest (p. 22). United States Department of Agriculture; National Agricultural Library.

[gbc21453-bib-0159] United States Forest Service . (1962). Annual fire report for the National forest (p. 22). United States Department of Agriculture; National Agricultural Library.

[gbc21453-bib-0160] United States Forest Service . (1963). Annual fire report for the National forest (p. 22). United States Department of Agriculture; National Agricultural Library.

[gbc21453-bib-0161] United States Forest Service . (1964a). Annual fire report for the National forest (p. 22). United States Department of Agriculture; National Agricultural Library.

[gbc21453-bib-0162] United States Forest Service . (1964b). Forest fire statistics 1964 (forest fire statistics) [Statistical report] (p. 29). United States Department of Agriculture; University of Florida.

[gbc21453-bib-0163] United States Forest Service . (1965). Annual fire report for the National forest (p. 22). United States Department of Agriculture; National Agricultural Library.

[gbc21453-bib-0164] United States Forest Service . (1966). Annual fire report for the National forest (p. 22). United States Department of Agriculture; National Agricultural Library.

[gbc21453-bib-0165] United States Forest Service . (1969). Annual fire report for the National forest (22). United States Department of Agriculture; National Agricultural Library.

[gbc21453-bib-0166] United States Forest Service . (1971). National forest fire report 1971 (p. 53). United States Department of Agriculture; National Agricultural Library.

[gbc21453-bib-0167] United States Forest Service . (1972). National forest fire report 1972 (p. 50). United States Department of Agriculture; National Agricultural Library.

[gbc21453-bib-0168] United States Forest Service . (1973). National forest fire report 1973 (p. 50). United States Department of Agriculture; National Agricultural Library.

[gbc21453-bib-0169] United States Forest Service . (1974). National forest fire report 1974 (p. 52). United States Department of Agriculture; National Agricultural Library.

[gbc21453-bib-0170] United States Forest Service . (1975). National forest fire report 1975 (p. 51). United States Department of Agriculture; National Agricultural Library.

[gbc21453-bib-0171] United States Forest Service . (1976). National forest fire report 1976 (p. 51). United States Department of Agriculture; National Agricultural Library.

[gbc21453-bib-0172] United States Forest Service . (1977). National forest fire report 1977 (p. 54). United States Department of Agriculture; National Agricultural Library.

[gbc21453-bib-0173] United States Forest Service . (1978). National forest fire report 1978 (p. 53). United States Department of Agriculture; National Agricultural Library.

[gbc21453-bib-0174] United States Forest Service . (1979). National forest fire report 1979 (p. 56). United States Department of Agriculture; National Agricultural Library.

[gbc21453-bib-0175] United States Forest Service . (1980). National forest fire report 1980 (p. 56). United States Department of Agriculture; National Agricultural Library.

[gbc21453-bib-0176] United States Forest Service . (1981). National forest fire report 1981 (p. 50). United States Department of Agriculture; National Agricultural Library.

[gbc21453-bib-0177] United States Forest Service . (1982). National forest fire report 1982 (p. 49). United States Department of Agriculture; National Agricultural Library.

[gbc21453-bib-0178] United States Forest Service . (1983). National forest fire report 1983 (p. 49). United States Department of Agriculture; National Agricultural Library.

[gbc21453-bib-0179] United States Forest Service . (1984). National forest fire report 1984 (p. 49). United States Department of Agriculture; National Agricultural Library.

[gbc21453-bib-0180] United States Forest Service . (1985). National forest fire report 1985 (p. 44). United States Department of Agriculture; National Agricultural Library.

[gbc21453-bib-0181] Urbanski, S. P. , Reeves, M. C. , Corley, R. E. , Silverstein, R. P. , & Hao, W. M. (2018). Contiguous United States wildland fire emission estimates during 2003–2015. Earth System Science Data, 10(4), 2241–2274. 10.5194/essd-10-2241-2018

[gbc21453-bib-0182] U.S. Bureau of the Census . (1984). Statistical Abstract of the United States: 1985 (no. 105). Government Printing Office. Retrieved from https://www2.census.gov/library/publications/1984/compendia/statab/105ed/1985-01.pdf

[gbc21453-bib-0183] USDA Forest Service . (1958). Timber resources for America’s future (report no. 14; forest resource report) (p. 719). U.S. Department of Agriculture, Forest Service.

[gbc21453-bib-0184] U.S. Department of Commerce and Labor . (1908). Statistical Abstract of the United States: 1907. Government Printing Office. Retrieved from https://www.census.gov/library/publications/1908/compendia/statab/30ed.html

[gbc21453-bib-0185] U.S. Department of Commerce and Labor . (1921). Statistical Abstract of the United States: 1920 (no. 43). Government Printing Office. Retrieved from https://www.census.gov/library/publications.html

[gbc21453-bib-0186] U.S. Department of Commerce and Labor . (1930). Statistical Abstract of the United States: 1930 (no. 52). Government Printing Office. Retrieved from https://www2.census.gov/library/publications/1930/compendia/statab/52ed/1930-01.pdf

[gbc21453-bib-0187] van Leeuwen, T. T. , van der Werf, G. R. , Hoffmann, A. A. , Detmers, R. G. , Rücker, G. , French, N. H. F. , et al. (2014). Biomass burning fuel consumption rates: A field measurement database. Biogeosciences, 11(24), 7305–7329. 10.5194/bg-11-7305-2014

[gbc21453-bib-0188] van Marle, M. J. E. , Kloster, S. , Magi, B. I. , Marlon, J. R. , Daniau, A.‐L. , Field, R. D. , et al. (2017). Historic global biomass burning emissions for CMIP6 (BB4CMIP) based on merging satellite observations with proxies and fire models (1750–2015). Geoscientific Model Development, 10(9), 3329–3357. 10.5194/gmd-10-3329-2017

[gbc21453-bib-0189] Wear, D. N. , & Coulston, J. W. (2015). From sink to source: Regional variation in U.S. forest carbon futures. Scientific Reports, 5(1), 16518. 10.1038/srep16518 26558439 PMC4642345

[gbc21453-bib-0190] Westerling, A. L. , Gershunov, A. , Brown, T. J. , Cayan, D. R. , & Dettinger, M. D. (2003). Climate and wildfire in the Western United States. Bulletin of the American Meteorological Society, 84(5), 595–604. 10.1175/BAMS-84-5-595

[gbc21453-bib-0191] Westerling, A. L. , Hidalgo, H. G. , Cayan, D. R. , & Swetnam, T. W. (2006). Warming and earlier spring increase western U.S. forest wildfire activity. Science, 313(5789), 940–943. 10.1126/science.1128834 16825536

[gbc21453-bib-0192] White, P. S. , MacKenzie, M. D. , & Busing, R. T. (1985). Natural disturbance and gap phase dynamics in southern Appalachian spruce–fir forests. Canadian Journal of Forest Research, 15(1), 233–240. 10.1139/x85-041

[gbc21453-bib-0193] Williams, C. A. , Gu, H. , MacLean, R. , Masek, J. G. , & Collatz, G. J. (2016). Disturbance and the carbon balance of US forests: A quantitative review of impacts from harvests, fires, insects, and droughts. Global and Planetary Change, 143, 66–80. 10.1016/j.gloplacha.2016.06.002

[gbc21453-bib-0194] Wilson, N. , Bradstock, R. , & Bedward, M. (2021). Comparing forest carbon stock losses between logging and wildfire in forests with contrasting responses to fire. Forest Ecology and Management, 481, 118701. 10.1016/j.foreco.2020.118701

[gbc21453-bib-0195] Wuerthner, G. (2006). Wildfire: A century of failed forest policy. Island.

[gbc21453-bib-0196] Xu, R. , Yu, P. , Abramson, M. J. , Johnston, F. H. , Samet, J. M. , Bell, M. L. , et al. (2020). Wildfires, Global Climate Change, and Human Health. New England Journal of Medicine, 383(22), 2173–2181. 10.1056/NEJMsr2028985 33034960

[gbc21453-bib-0197] Yang, J. , Tian, H. , Tao, B. , Ren, W. , Pan, S. , Liu, Y. , & Wang, Y. (2015). A growing importance of large fires in conterminous United States during 1984–2012. Journal of Geophysical Research: Biogeosciences, 120(12), 2625–2640. 10.1002/2015JG002965

[gbc21453-bib-0198] Zhang, F. , Chen, J. M. , Pan, Y. , Birdsey, R. A. , Shen, S. , Ju, W. , & He, L. (2012). Attributing carbon changes in conterminous U.S. forests to disturbance and non‐disturbance factors from 1901 to 2010: Attributing carbon changes of U.S. Forests. Journal of Geophysical Research, 117(G2), G02021. 10.1029/2011JG001930

[gbc21453-bib-0199] Zhou, Y. , Williams, C. A. , Hasler, N. , Gu, H. , & Kennedy, R. (2021). Beyond biomass to carbon fluxes: Application and evaluation of a comprehensive forest carbon monitoring system. Environmental Research Letters, 16(5), 055026. 10.1088/1748-9326/abf06d

[gbc21453-bib-0200] Haberl, H. , Erb, K.‐H. , & Krausmann, F. (2014). Human Appropriation of net primary production: Patterns, trends, and planetary boundaries. Annual Review of Environment and Resources, 39(1), 363–391. 10.1146/annurev-environ-121912-094620

[gbc21453-bib-0201] Howard, J. L. , & Liang, S. (2019). US timber production, trade, consumption, and price statistics, 1965–2017. Research paper FPL‐RP‐701 (Vol. 701, pp. 1–96). US Department of Agriculture, Forest Service, Forest Products Laboratory.

[gbc21453-bib-0202] IEA . (2015). Extended world energy balances. OECD Publishing. Retrieved from http://www.oecd-ilibrary.org/energy/data/iea-world-energy-statistics-and-balances/extended-world-energy-balances_data-00513-en

[gbc21453-bib-0203] Ince, P. J. (2000). Industrial wood productivity in the United States, 1900–1998 (Vol. 272). US Department of Agriculture, Forest Service, Forest Products Laboratory.

[gbc21453-bib-0204] Kastner, T. , Chaudhary, A. , Gingrich, S. , Marques, A. , Persson, U. M. , Bidoglio, G. , et al. (2021). Global agricultural trade and land system sustainability: Implications for ecosystem carbon storage, biodiversity, and human nutrition. One Earth, 4(10), 1425–1443. 10.1016/j.oneear.2021.09.006

[gbc21453-bib-0205] Manthy, R. S. , & Potter, N. (1978). Natural resource commodities: A century of statistics: Prices, output, consumption, foreign trade, and employment in the United States, 1870–1973. Published for Resources for the Future by the Johns Hopkins University Press.

[gbc21453-bib-0206] Reynolds, R. V. R. , & Pierson, A. H. (1942). Fuel wood used in the United States, 1630–1930 (Issue 641). US Department of Agriculture.

[gbc21453-bib-0207] Schurr, S. H. , & Netschert, B. C. (1960). Energy in the American economy, 1850–1975, an economic study of its history and prospects. Johns Hopkins Press.

[gbc21453-bib-0208] United Nations Statistics Division . (2020). UN fuelwood database. Retrieved from https://data.un.org/Data.aspx?d=EDATA&f=cmID%3AFW%3BtrID%3A1231

[gbc21453-bib-0209] U.S. Energy Information Administration . (2020). Total end‐use energy consumption 1960–2017 United States.

[gbc21453-bib-0210] U.S. Geological Survey . (2018). USGS commodity statistics and information. U.S. Department of the Interior. Retrieved from https://minerals.usgs.gov/minerals/pubs/commodity/

[gbc21453-bib-0211] USDA Forest Service . (1932). Forest situation in the United States [Special report] (p. 101). United States Department of Agriculture.

[gbc21453-bib-0212] USDA Forest Service . (1974). The outlook for timber in the United States (general technical report no. 20; forest resource report). U.S. Department of Agriculture, Forest Service (p. 390). Retrieved from https://www.fs.fed.us/research/docs/rpa/pre-1989/1974%20US%20Timber%20Outlook.pdf

[gbc21453-bib-0213] Walker, A. P. , De Kauwe, M. G. , Bastos, A. , Belmecheri, S. , Georgiou, K. , Keeling, R. F. , et al. (2021). Integrating the evidence for a terrestrial carbon sink caused by increasing atmospheric CO_2_ . New Phytologist, 229(5), 2413–2445. 10.1111/nph.16866 32789857

[gbc21453-bib-0214] Wang, S. , Zhang, Y. , Ju, W. , Chen, J. M. , Ciais, P. , Cescatti, A. , et al. (2020). Recent global decline of CO_2_ fertilization effects on vegetation photosynthesis. Science, 370(6522), 1295–1300. 10.1126/science.abb7772 33303610

[gbc21453-bib-0215] Warde, P. (2019). Firewood consumption and energy transition: A survey of sources, methods and explanations in Europe and North America. Historia Agraria: Revista de Agricultura e Historia Rural, 77, 7–32. 10.26882/histagrar.077e02w

