# Supplementary material for: The Role of Wildfires in the Interplay of Forest Carbon Stocks and Wood Harvest in the Contiguous United States During the 20th Century
Source: Global Biogeochem Cycles. 2023 Aug 17;37(8):e2023GB007813. doi: 10.1029/2023GB007813 (PMC10909529; doi:10.1029/2023GB007813)
Supplement: Supplementary file 1 — Supporting Information S1 [file GBC-37-e2023GB007813-s001.docx]

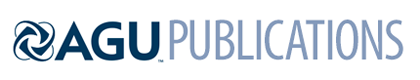


*[Global Biogeochemical Cycles]*

Supporting Information for

**The role of wildfires in the interplay of forest carbon stocks and wood harvest in the contiguous United States during the 20th century**

Andreas Magerl^1^, Simone Gingrich^1^, Sarah Matej^1^, Geoff Cunfer^2^, Matthew Forrest^3^, Christian Lauk^1^, Stefan Schlaffer^4^, Florian Weidinger^1^, Cody Yuskiw^5^, Karl-Heinz Erb^1^

1. University of Natural Resources and Life Sciences, Vienna, Institute of Social Ecology, Austria
2. University of Saskatchewan, Saskatoon, Department of History, Canada
3. Senckenberg Gesellschaft für Naturforschung, Frankfurt am Main, Germany
4. Vienna University of Technology, Vienna, Austria
5. University of Saskatchewan, Saskatoon, College of Law, Canada

## Abbreviations

C: carbon
DM: dry matter
ha: hectare
hr: hour
NLCD: National Land Cover Database
NPP_pot_: Potential net primary production
t: tonne
USFS: United States Forest Service
yr: year

# Contents of this file

[1. Forest categories used for reconstruction of burned area 2](#_Toc132632062)

[2. Landsat Burned area data preparation 2](#_Toc132632063)

[Text S1. 2](#_Toc132632064)

[3. Fuel loadings allocation, combustion completeness factors 3](#_Toc132632065)

[4. Total burned forest area by states, 1941-1960, regions and categories 1941-2017 6](#_Toc132632066)

[5. Wood harvest estimation 8](#_Toc132632746)

[Text S2 9](#_Toc132632747)

[6. Scatterplots of state-level removals and stock densities 11](#_Toc132632748)

[7. NPP_pot_ calculation 12](#_Toc132632749)

[Text S3 12](#_Toc132632750)

[8. Sensitivity analysis 13](#_Toc132632752)

[Text S4 13](#_Toc132632753)

[Text S5 16](#_Toc132632754)

[9. Other Landsat burned area 19](#_Toc132632755)

[10. References 19](#_Toc132632760)

## Forest categories used for reconstruction of burned area

| **Aggregated category used in this study** | **USFS forest category**  **1941-1985** | **Administrative unit** | **NLCD classification used for Landsat Burned Area Algorithm (Hawbaker et al., 2020) 1985-2017** | | **Administrative unit** |
| --- | --- | --- | --- | --- | --- |
| *State & private forest* | Forest (protected, unprotected) | State & private | Forest | Deciduous Forest | State & private |
|  |  |  |  | Evergreen Forest | State & private |
|  |  |  |  | Mixed Forest | State & private |
|  |  |  |  |  |  |
| *Federal forest* | Forest (protected, unprotected) | Federal | Forest | Deciduous Forest | Federal |
|  |  |  |  | Evergreen Forest | Federal |
|  |  |  |  | Mixed Forest | Federal |
| *Other forest* | other protected, non-forest | Federal, state & private, or not specified | Shrubland | Shrub/scrub | Federal  State & private |

**Table S1.** Aggregated forest and shrub/woodland categories used in this study based on USFS forest fire statistics and allocated NLCD classifications used in Hawbaker et al., (2020)

## Landsat Burned area data preparation

## Text S1.

*Data preparation*

The Landsat burned area product (Hawbaker et al., 2020) was aggregated by taking into account state boundaries, NLCD land cover (LC) class and information about property (federal land or not). The aggregation was done using Zonal Statistics in ArcGIS. Zones were created by intersecting states, and federal land as a binary raster (1: federal, 0: other) and the NLCD LC class. The zonal statistics computed for each zone the number of pixels (COUNT) and total burned area (AREA) in each zone (VALUE) per year.

*Aggregation*

In order to make NLCD classes comparable between the 1992 version and later versions (2001 onward), the classes were reclassified according to Table S4 in Hawbaker et al. (2020). The following changing classes were taken into account: "Developed", "Cultivated Crops", "Grassland/Herbaceous".

## Fuel loadings allocation, combustion completeness factors

|  |  | Fuel loads from Urbanski et al. (2018) Table 4 | | | | | | |
| --- | --- | --- | --- | --- | --- | --- | --- | --- |
|  |  | Litter/Duff | Down deadwood | | | | Live fuels | |
|  |  |  | 1 hr (<1cm diameter) | 10 hr (1-2.5 cm) | 100 hr (2.5-7.6) | Sound logs (s3to9, s9to20, sgt20)/Rotten logs (r3to9, r9to20, rgt20) (7.6->50.8 cm) | Herb/Shrub | Available canopy fuel |
|  |  | As used in this study for base year 2017 | | | | | | |
|  |  | Duff & litter (average per region) | 1hr  (average per region) | 10hr  (average per region) | 100hr  (average per region) | 1000hr  (average of sub-categories per region) | Grass, herb, Shrub  (average of subcategories per region) | Canopy |
| **Region** | **Forest category** | **[t DM/ha]** | | | | | | |
| Northeast | Federal Forest | 52.30 | 9.45 | 1.08 | 3.60 | 7.78 | 0.30 | 5.20 |
|  | Other forest | 52.30 | 9.45 | - | - | - | 0.30 | - |
|  | State & Private forest | 52.30 | 9.45 | 1.08 | 3.60 | - | 0.30 | 5.20 |
| Southeast | Federal Forest | 24.25 | 26.75 | 1.00 | 3.00 | 3.33 | 0.33 | 1.60 |
|  | Other forest | 24.25 | 26.75 | - | - | - | 0.33 | - |
|  | State & Private forest | 24.25 | 26.75 | 1.00 | 3.00 | - | 0.33 | 1.60 |
| Rocky Mountains | Federal Forest | 16.45 | 10.35 | 1.10 | 3.70 | 26.79 | 1.39 | 9.59 |
|  | Other forest | 16.45 | 10.35 | - | - | - | 1.39 |  |
|  | State & Private forest | 16.45 | 10.35 | 1.10 | 3.70 | - | 1.39 | 9.59 |
| Pacific Coast | Federal Forest | 21.55 | 16.78 | 1.23 | 3.85 | 15.05 | 1.58 | 4.93 |
|  | Other forest | 21.55 | 16.78 | - | - | - | 1.58 | - |
|  | State & Private forest | 21.55 | 16.78 | 1.23 | 3.85 | - | 1.58 | 4.93 |

**Table S2.** Average fuel-loads per area, by forest category and region as used in this study. Original data obtained from Urbanski et al. 2018.

States aggregated to regions: **Northeast**: Connecticut, Delaware, Maine, Maryland, Massachusetts, New Hampshire, New Jersey, New York, Pennsylvania, Rhode Island, Vermont, West Virginia, Illinois, Indiana, Iowa, Michigan, Minnesota, Missouri, Ohio, Wisconsin **Southeast**: Florida, Georgia, North Carolina, South Carolina, Virginia, Alabama, Arkansas, Kentucky, Louisiana, Mississippi, Oklahoma, Tennessee, Texas **Rocky Mountains**: Kansas, Nebraska, North Dakota, South Dakota, Arizona, Colorado, Idaho, Montana, Nevada, New Mexico, Utah, Wyoming **Pacific Coast**: Oregon, Washington, California

Fuel loads allocated to regions using the National Forest Type dataset by Ruefenacht et al. (2008) available at: <https://data.fs.usda.gov/geodata/rastergateway/forest_type/>

| **a**  **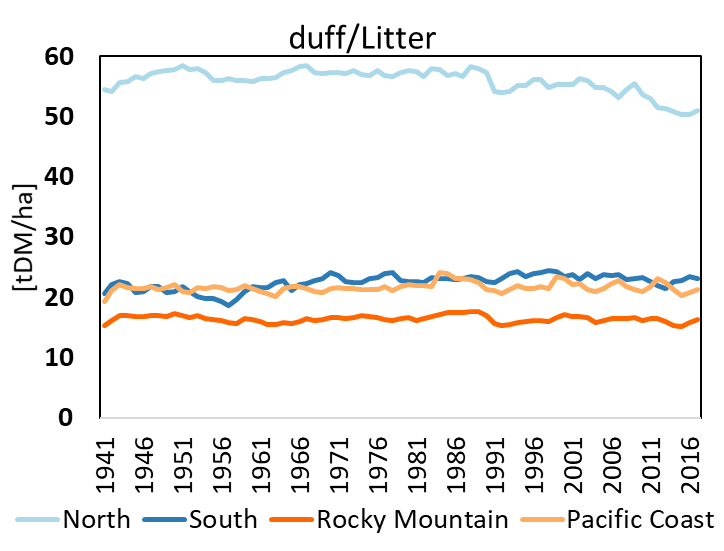** | **b**  **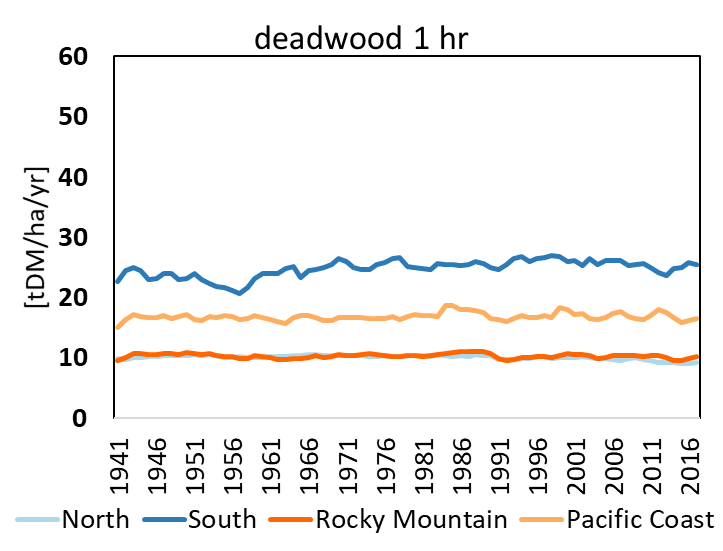** |
| --- | --- |
| **c**  **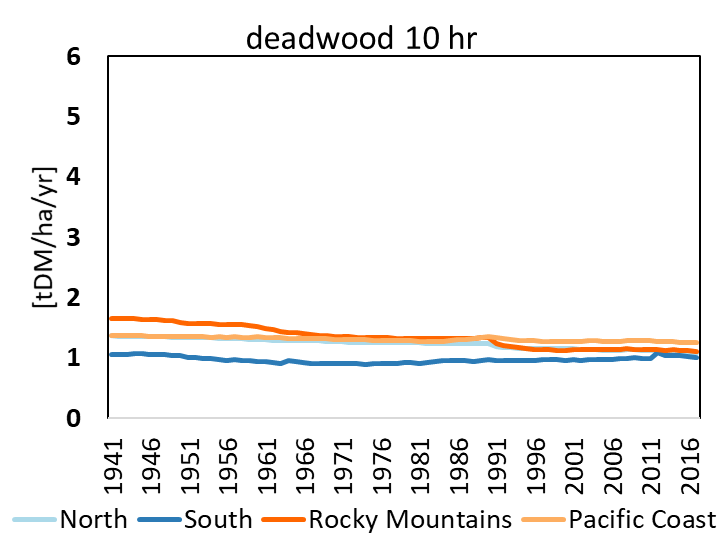** | **d**  **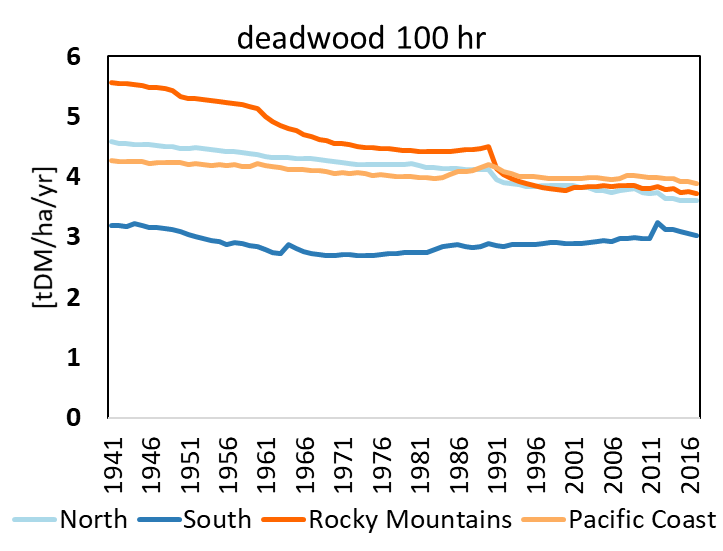** |
| **e**  **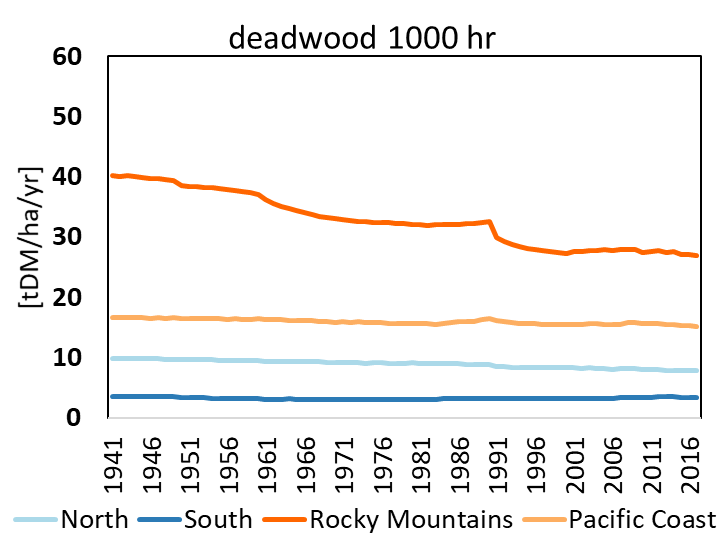** | **f**  **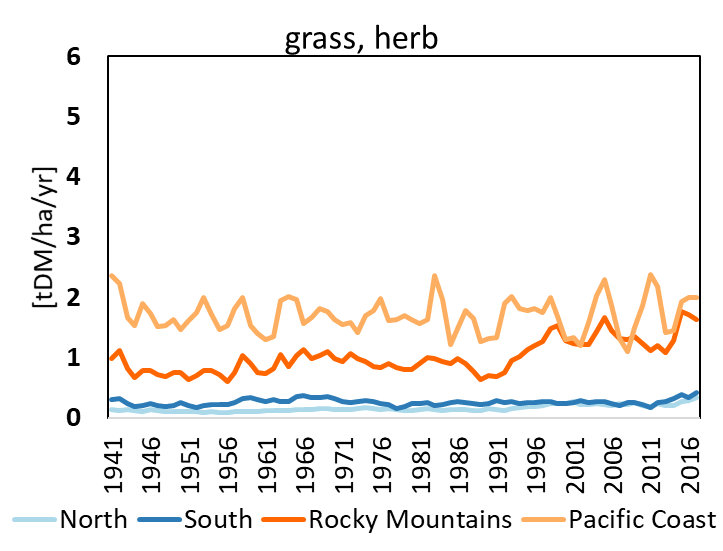** |
| **g**  **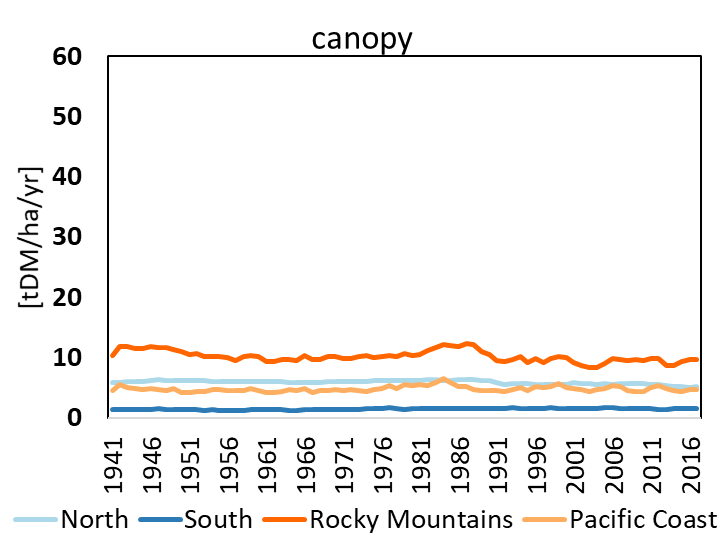** | **Figure S1**. Yearly average fuel loadings per area for 2017 based on field-measurements (Urbanski et al. 2018), modelled for 76 years using LPJ-GUESS SPITFIRE, by region and component **a**) duff/litter **b**) 1hr deadwood **c**) 10hrs deadwood **d**) 100hrs deadwood **e**) 1000hrs deadwood **f**) grass/herb **g**) canopy |

| **a**  **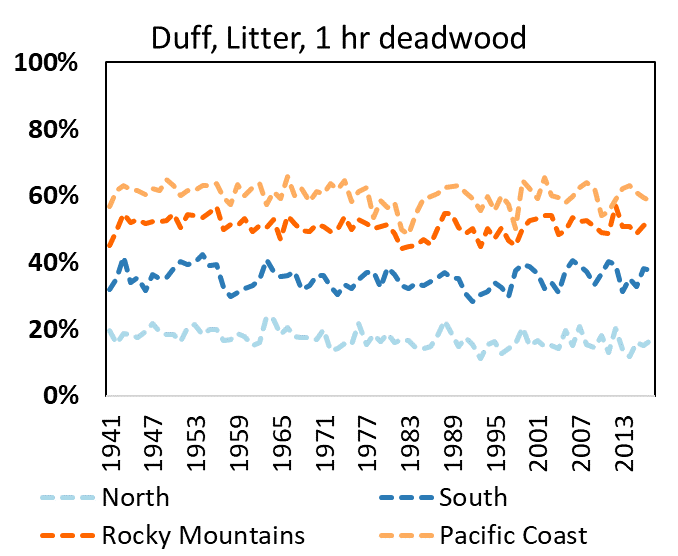** | **b**  **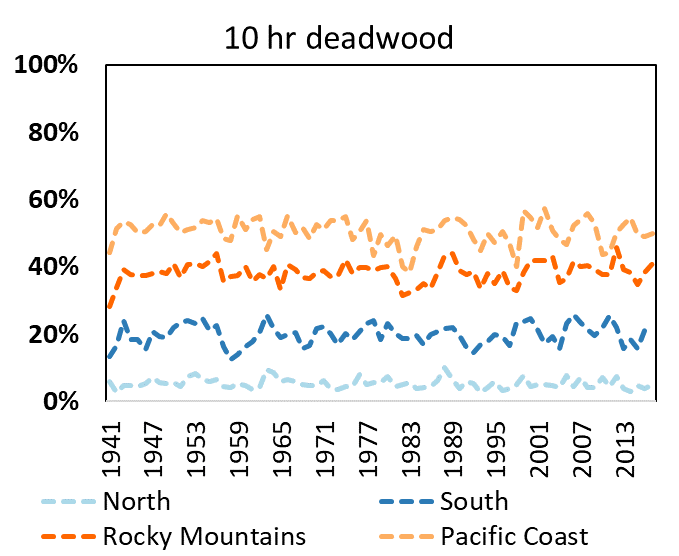** |
| --- | --- |
| **c**  **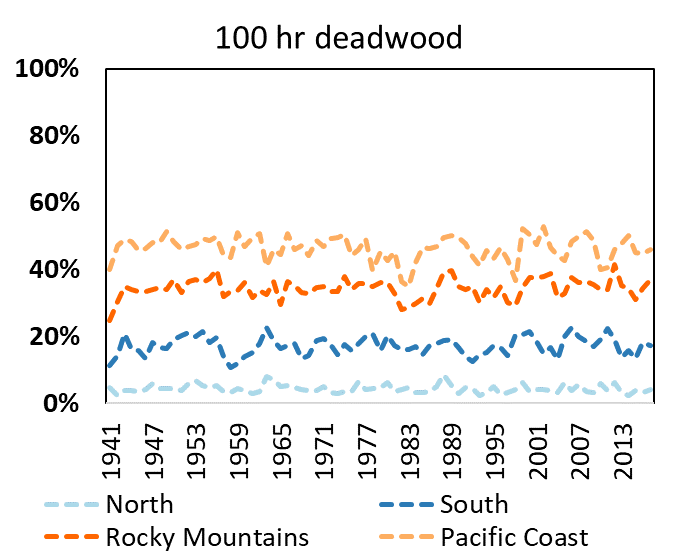** | **d**  **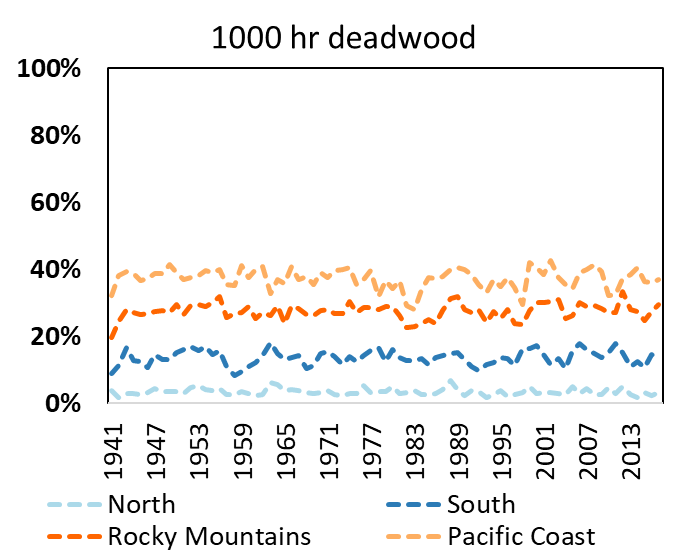** |
| **e**  **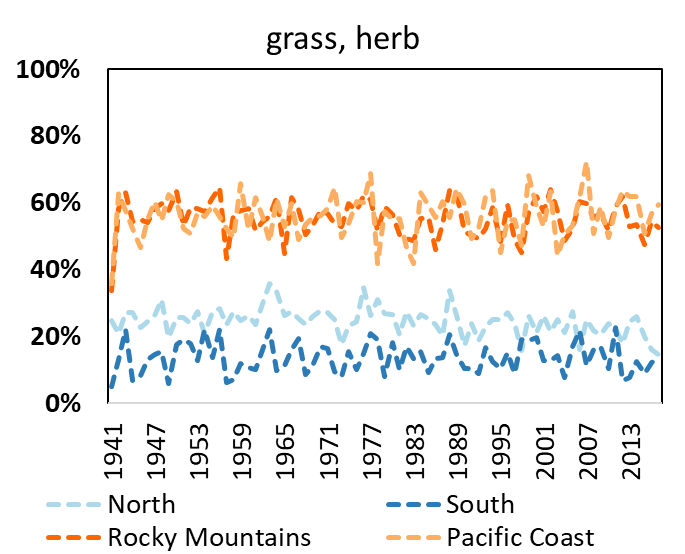** | **Figure S2**. Combustion completeness factors (% of total fuel loads burned) by fuel categories and regions modelled by LPJ-GUESS SPITFIRE for **a**) Duff, litter, and 1hr deadwood **b**) 10hrs deadwood **c**) 100hrs deadwood **d**) 1000hrs deadwood **e**) grass and herbs. For Canopy fuels we assumed constant moderate severity combustion completeness (49%) for all regions, as published by Yang et al., (2015) |

## Total burned forest area by states, 1941-1960, regions and categories 1941-2017


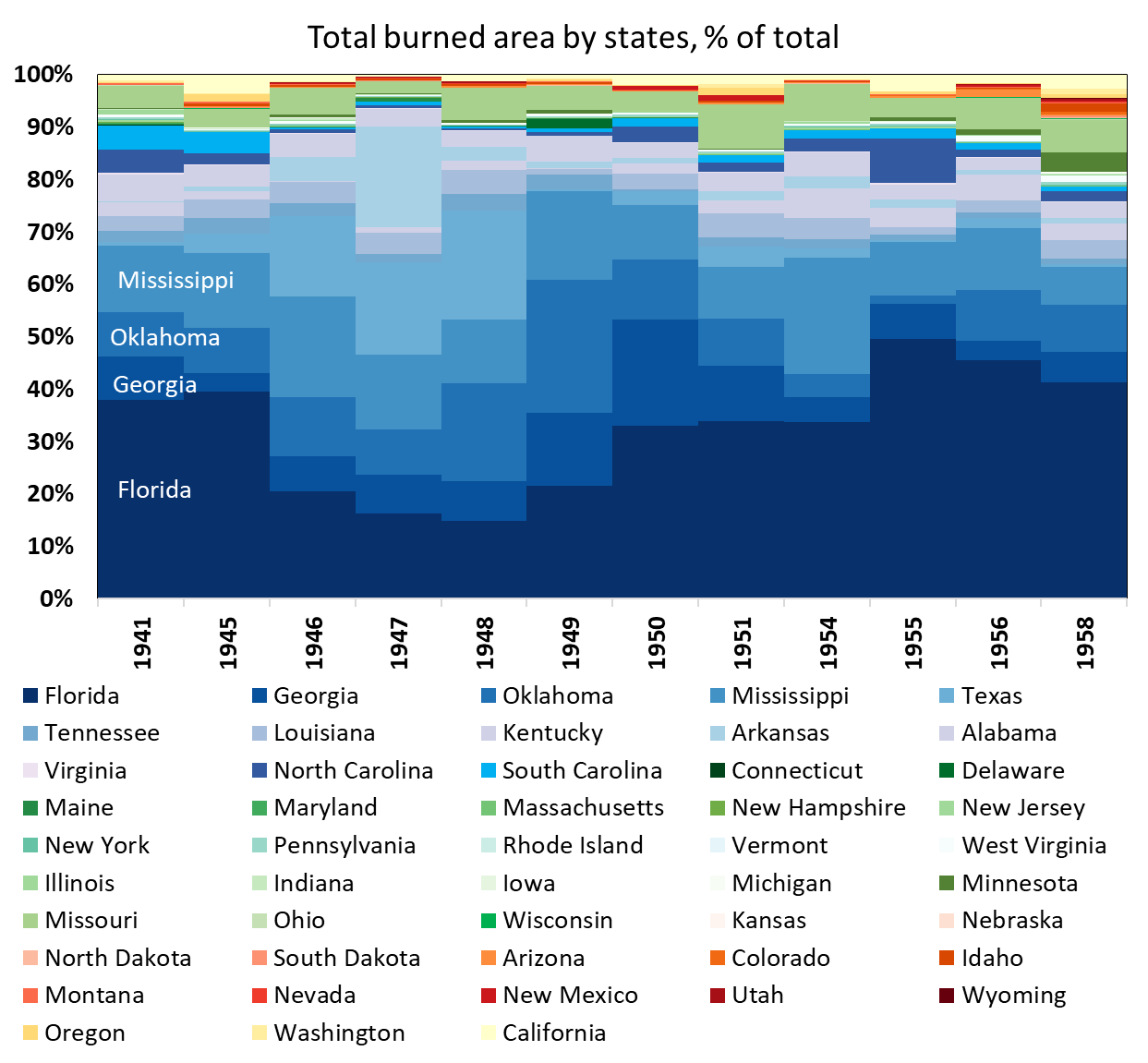


**Figure S3.** Total burned forest area (federal and state & private forests, other forests) by states and RPA Regions, Contiguous U.S. 1941-1960; Blue shades: South-eastern states; Green shades: North-eastern states; Red shades: Rocky Mountain states; Yellow shades: Pacific Coast states.

| **a)**  **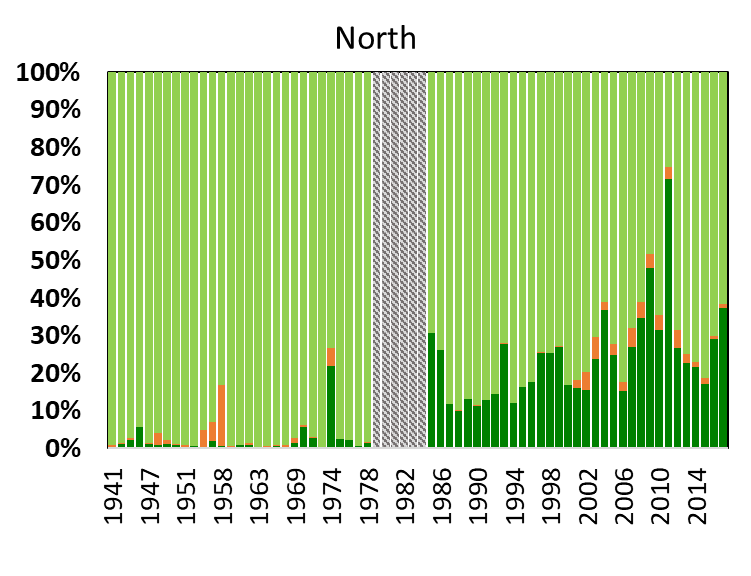** | **b)**  **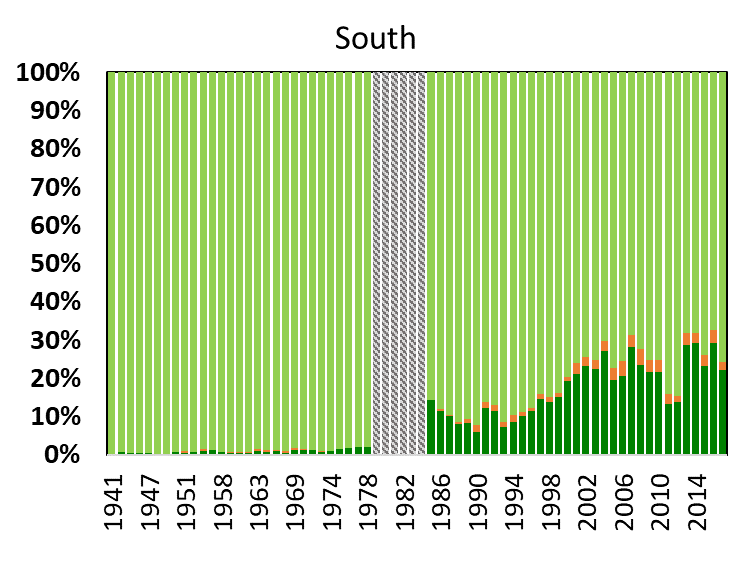** |
| --- | --- |
| **c)**  **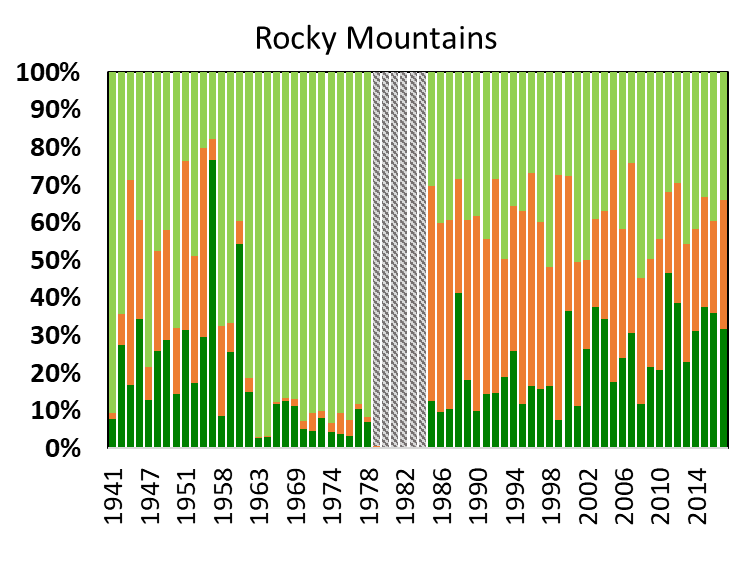** | **d)**  **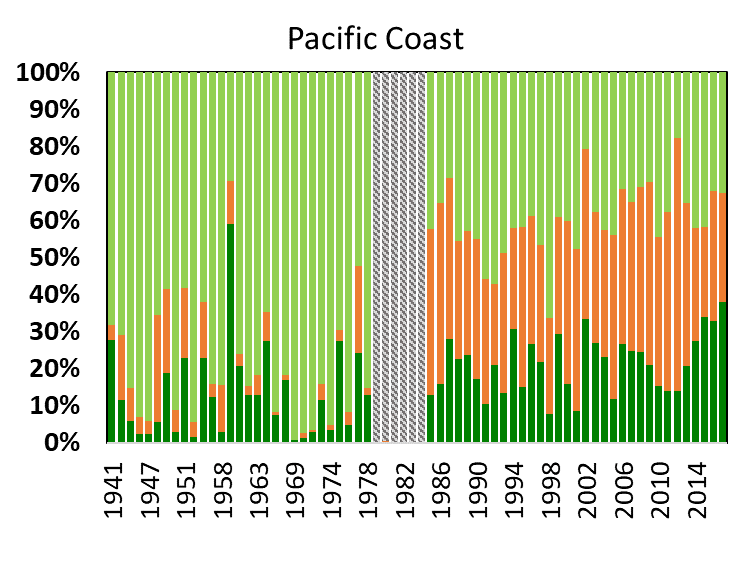** |
| 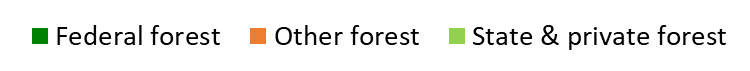  **Figure S4.** Total reconstructed burned area, by regions and forest categories. **a**) Northeast **b**) Southeast **c**) Rocky Mountains **d**) Pacific Coast, contiguous U.S. 1941-2017. Refer to main text section 2 for data sources and methodology used to create this figure. Note: 1979-1984 excluded due to data gap. | |

## Wood harvest estimation

| **a**  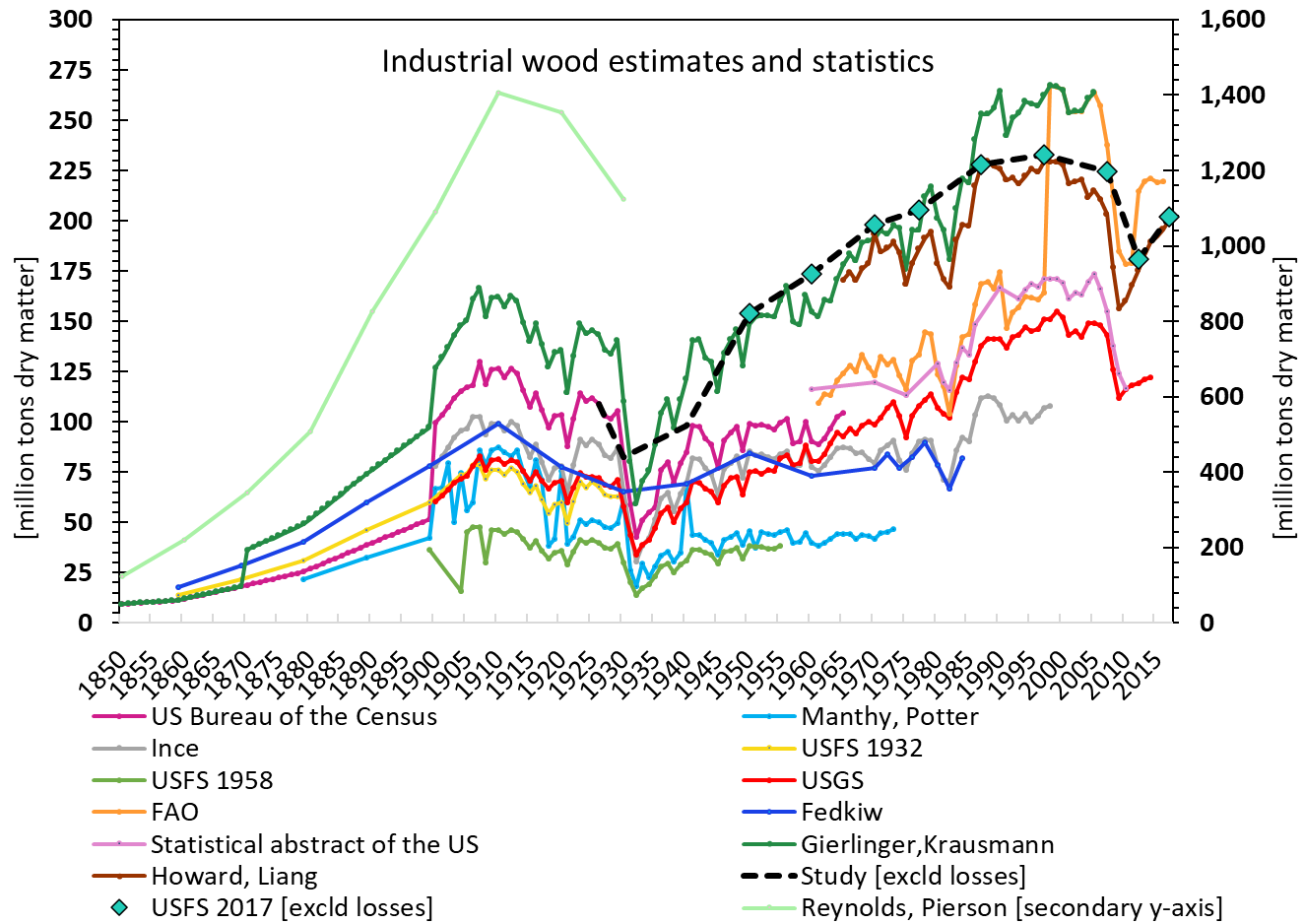 |
| --- |
| **b**  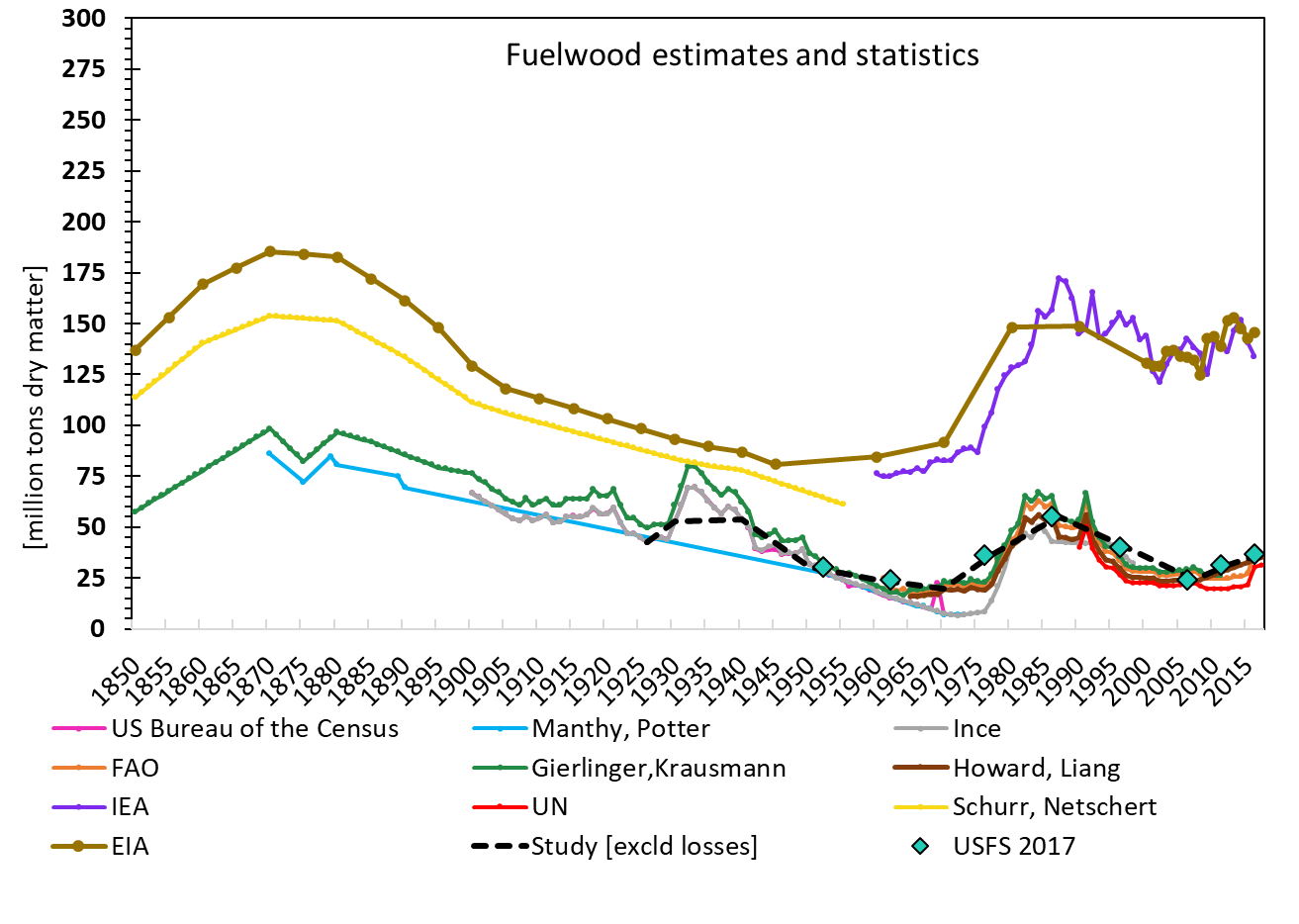 |

**Figure S5.** Data sources used for comparison and reconstruction of total U.S. wood harvest. **a**) Total industrial wood production **b**) total fuelwood production. 1850-2017. See table S3 for references.

| \| **Abbreviation** \| **Reference** \| \| --- \| --- \| \| Gierlinger, Krausmann \| (Gierlinger & Krausmann, 2012) \| \| Manthy, Potter \| (Manthy & Potter, 1978) \| \| Ince \| (Ince, 2000) \| \| Schurr, Netschert \| (Schurr & Netschert, 1960) \| \| Fedkiw \| (Fedkiw, 1989) \| \| Reynolds, Pierson \| (Reynolds & Pierson, 1942) \| \| Howard, Liang \| (Howard & Liang, 2019) \| \| US Bureau of the Census \| (United States Bureau of the Census, 1975) \| \| USFS 1932 \| (USDA Forest Service, 1932) \| \| USFS 1958 \| (United States Forest Service, 1958) \| \| USFS 2017 \| (Oswalt et al., 2018) \| \| USGS \| (U.S. Geological Survey, 2018) \| \| Statistical abstract of the United States \| (U.S. Bureau of the Census, 1984; U.S. Department of Commerce and Labor, 1908, 1921, 1930) \| \| UN \| (United Nations Statistics Division, 2020) \| \| IEA \| (IEA, 2015) \| \| EIA \| (U.S. Energy Information Administration, 2020) \| \| FAO \| (FAO, 2020) \|   **Table S3.** Abbreviations and references for the data sources used in this study (Figure S 3) for comparing and reconstructing historical industrial wood and fuelwood harvest in the contiguous United States. |
| --- | --- | --- | --- | --- | --- | --- | --- | --- | --- | --- | --- | --- | --- | --- | --- | --- | --- | --- | --- | --- | --- | --- | --- | --- | --- | --- | --- | --- | --- | --- | --- | --- | --- | --- | --- | --- |
| Text S2. **Figure S5** and **Table S3** represents, to the best of our knowledge, all known data sources on historical timber production and harvest in the U.S. at the national level. All data have been converted to tonnes of dry matter using the factors presented in **Table S4**. The majority of sources show a similar trend, but at different levels. As Warde (2019) showed, Reynolds and Pierson’s estimate, which is most likely a best-guess estimate inferred from per capita wood consumption, is almost 10 times higher than all other data sources, and is, although widely used in many studies, most likely an overestimation. The differences in the other sources can be explained by the fact that not all of them report data for all products for which wood was harvested. For example, the USFS 1958 series reports only lumber, but not the amounts of wood harvested for paper and pulp, veneer, and other products.  In contrast, the USFS 2017 reports all wood products harvested (saw logs, veneer, pulpwood and composites, and other products like poles, pilings, posts), hence we assume it is the most comprehensive and complete record among all those examined. We used this data source for our reconstruction of industrial wood harvest, as it also provides regionally disaggregated data for the four regions considered in this study for the period 1953-2017 in roughly 10-year intervals. For 1970, we added data from the USDA Forest Service's timber outlook report (1974, not shown in figure S5 and Table S3), which is consistent with the later USFS forest inventory report (Oswalt et al., 2018). For the period 1926 to 1950, we used data from the U.S. Bureau of the Census, which also reports complete wood products for this period. Gierlinger and Krausmann's calculation shows the same trajectory, as it is also largely based on this source, but is higher than the Bureau of the Census data. This can be explained by the fact that the former also includes an estimate of apparent consumption of miscellaneous timber products. However, as our reconstruction calculates the total timber *harvest*, we explicitly excluded consumption figures (as they often also include imported products).  Similarly, we compared some of the sources already used for industrial wood harvest plus additional ones as starting point for reconstructing fuelwood harvest (**Figure S5b**). EIA, Schurr & Netschert, IEA and Howard and Liang’s data all lie above our reconstruction, because they include mill and agricultural residues used for energy purposes. These residues would represent a double counting of originally harvested material or lie outside of our system boundaries, hence, we did not choose for these data sources. Just as for Industrial wood harvest, our reconstruction is based on the regionally disaggregated USFS 2017 data for the 1953-2017 period and U.S. Bureau of the Census for 1926-1953. Factors presented in **table S5** and **S6** were used to convert wood harvest figures from volume to weight and expanding for harvest losses.   \| **Factors for converting units of measurement to metric and English units**  Ref.: (Howard & Liang, 2019) \| \| \| \| --- \| --- \| --- \| \| **Unit** \| **Conversion factor** \| **Metric and English unit** \| \| square foot \| 0.09290 \| square meter \| \| cubic foot (log trade) \| 0.02832 \| cubic meter \| \| short tons (chips) \| 0.01850 \| 1,000 cubic feet \| \| board foot (hardwood lumber) \| 0.00236 \| cubic meter \| \| board foot (softwood lumber) \| 0.00170 \| cubic meter \| \| board foot (lumber export and imports) \| 0.00236 \| cubic meter \| \| board foot (logs) \| 0.00453 \| cubic meter \| \| 1,000 square feet (1/8-in. panels) \| 0.29500 \| cubic meter \| \| 1,000 square feet (1/4-in. panels) \| 0.59000 \| cubic meter \| \| 1,000 square feet (3/8-in. panels) \| 0.88500 \| cubic meter \| \| 1,000 square feet (3/8-in. panels) \| 2.03600 \| square foot (surface measure) \| \| 1,000 square feet (1/2-in. panels) \| 1.18000 \| cubic meter \| \| 1,000 square feet (3/4-in. panels) \| 1.77000 \| cubic meter \| \| cubic meter \| 0.00230 \| million square feet (surface measure) \| \| square meter \| 10.76390 \| square foot (surface measure) \| \| cord \| 2.27000 \| cubic meter \| \| cord \| 2.65000 \| green ton \| \| ton \| 0.00030 \| 1,000 cords \| \| ton (short ton) \| 0.90700 \| metric ton \|   **Table S4.** Conversion factors used in this study for different weight and volume units of reported industrial wood products and fuelwood for the contiguous U.S. |
| \| **Standard factors to convert quantities given in volume (cubic meter) into weight (tons)**  Ref.: (Krausmann et al., 2013) \| \| \| \| --- \| --- \| --- \| \| **Wood type and unit** \| **Conversion factor** \| **Unit** \| \| coniferous/softwood [cubic meter] \| 0.405 \| metric ton \| \| non-coniferous/hardwood [cubic meter] \| 0.576 \| metric ton \| \| Average [cubic meter] \| 0.491 \| metric ton \|   **Table S5.** Conversion factors used in this study for converting from volume of wood to specific weight per wood types for industrial wood products and fuelwood the contiguous U.S.   \| **Industrial & fuelwood harvest loss factors**  Ref.: (Krausmann et al., 2013) \| \| \| \| \| --- \| --- \| --- \| --- \| \| **Bark factor** \| **Recovery rate** \| **belowground (root-to-shoot) ratio** \| **species** \| \| 88% \| 90% \| 79% \| coniferous \| \| 89% \| 79% \| 79% \| non-coniferous \|   **Table S6.** Expansion factors used in this study for estimating harvest losses for industrial and fuel wood harvest for the contiguous U.S. |

## Scatterplots of state-level removals and stock densities

| **a**  **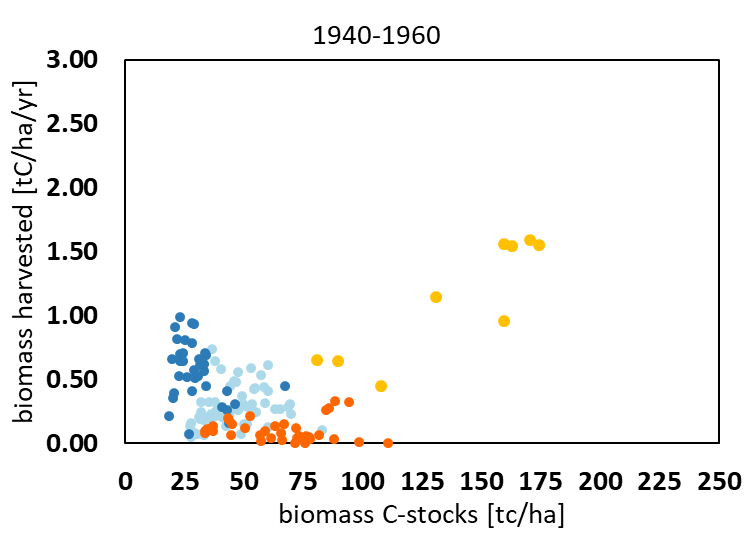** | **b**  **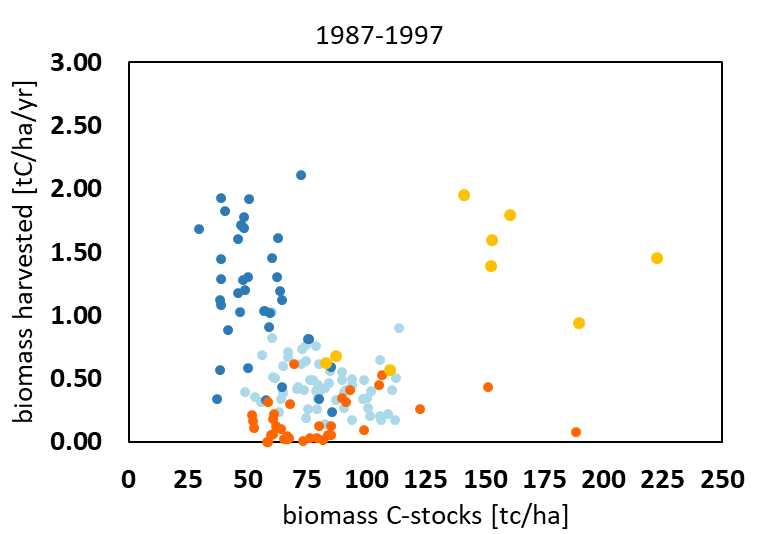** | **c**  **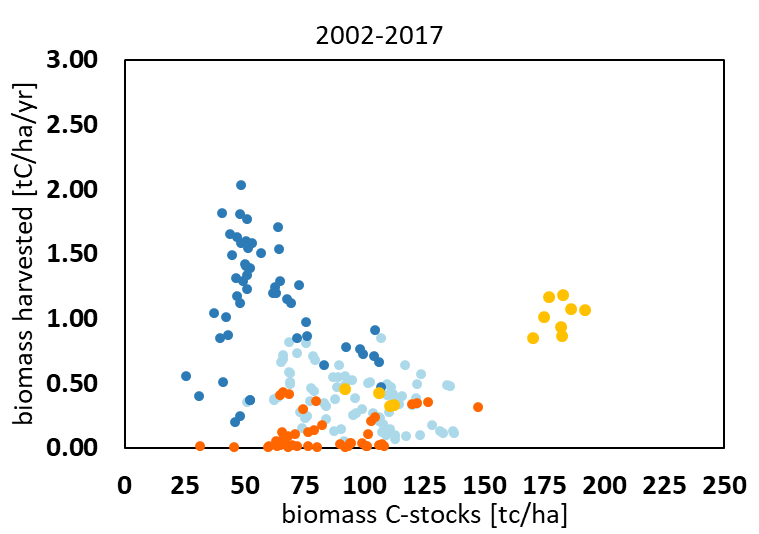** |
| --- | --- | --- |
| **d**  **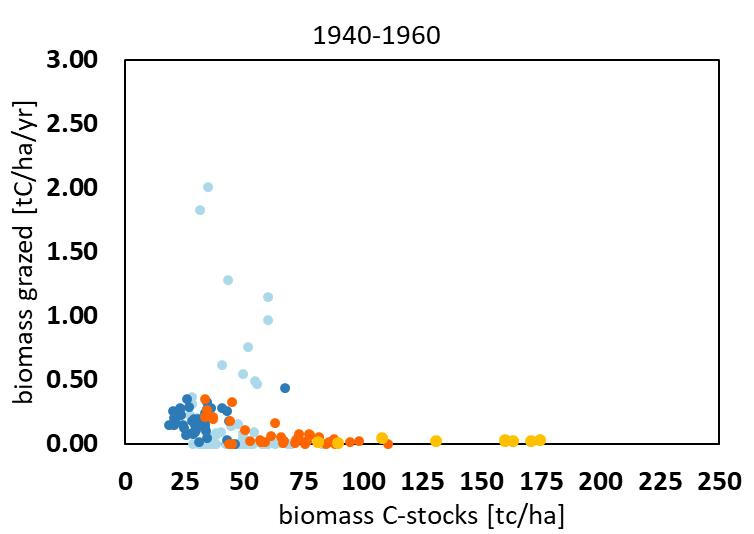** | **e**  **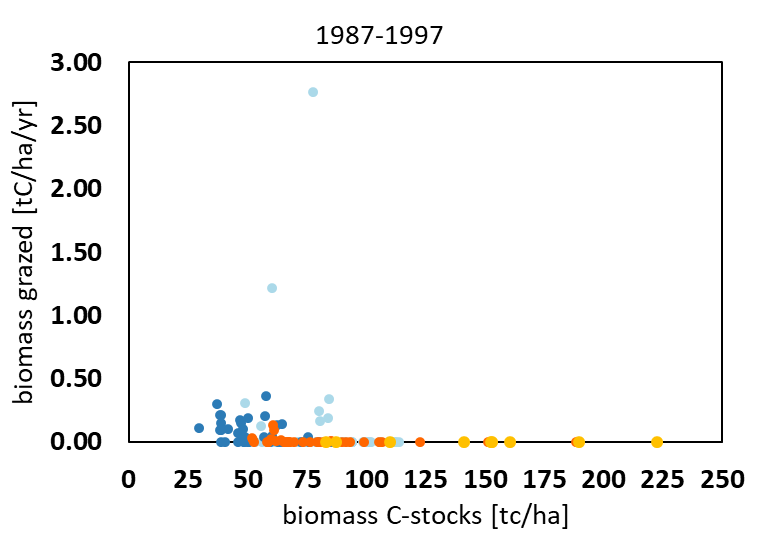** | **f**  **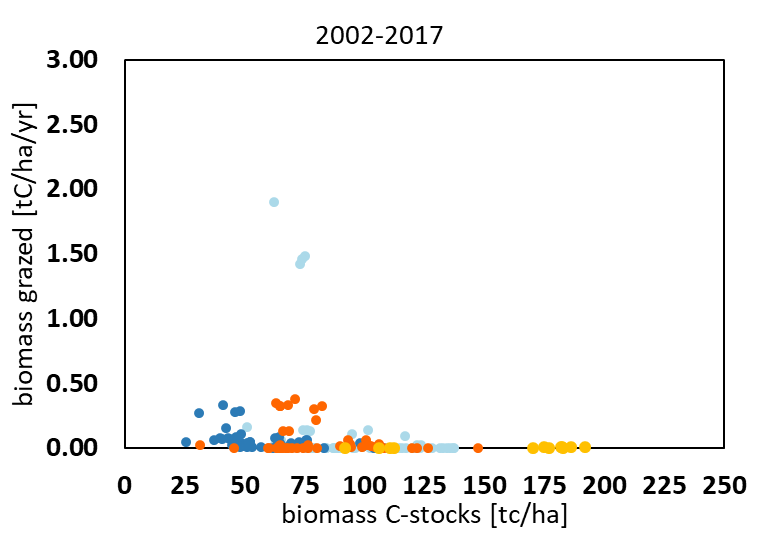** |
| **Figure S6**. Scatterplots of biomass harvest [y-axis, tC/ha/yr] and biomass stock density [x-axis, tC/ha] (upper row) and scatterplots of biomass grazed [y-axis, tC/ha/yr] and biomass stock density [x-axis, tC/ha] (lower row) for the contiguous 48 states **a, d**) 1940-1960 **b, e**) 1987-1997 **c, f**) 2002-2017. Time frames are determined by the availability of complete state-level data. Light blue dots: North; Dark blue dots: South; Orange dots: Rocky Mountains; Yellow dots: Pacific Coast. | | |

## NPP_pot_ calculation

Text S3**.** To consider the possible relative effect of changing climatic conditions on the forest C stock trajectories, we analysed the potential Net Primary Productivity (NPP_pot_) of forests. NPP_pot_ data calculated with LPJ-GUESS version 4.0.1., was taken from Kastner et al. (2022). NPP_pot_ is defined as the NPP of the potential vegetation of ecosystems that would prevail under the hypothetical absence of human land use but with current climatic conditions (Haberl et al., 2014). We removed NPP_pot_ values below zero (n=15) and calculated 5-year averages from the yearly NPP_pot_ model output. To combine the land-use data at 5 arcmin resolution from Kastner et al. (2021) with NPP_pot_ data at 30 arcmin resolution, the NPP_pot_ value in gC/m² per 30 arcmin grid cell was used for all 5 arcmin cells contained per 30 arcmin grid cell. Based on visual inspection, wilderness areas from Kastner et al. (2021) in the contiguous U.S. were found to also cover sparsely forested regions in the Rocky Mountains and Pacific Coast regions that are included in national forest statistics (i.e., “other forests” in this study) and thus were also considered in the average forest NPP_pot_ per ha calculation.

NPP_pot_ per area increased in all regions throughout the contiguous U.S over the 20^th^ century, due to changing climate, with slightly steeper slopes in the Northeast and Southeast than in the Rocky Mountains and the Pacific Coast (**Figure S7**). The low r² of the linear correlations between C densities and NPP_pot_ per area indicate generally weak trends, except for the Northeast, suggesting that increased growth conditions were not the most important driver for the observed increase in the national forest C stock sink. Evidence for plant response to enhanced CO_2_ concentration in the atmosphere has been found across dynamic vegetation models and in eddy-covariance towers measurements (Haverd et al., 2020; Walker et al., 2021). While this development may contribute to altered growth conditions, to what extent this increase is responsible for actual enhanced vegetation growth is unclear and hotly debated, as it is not directly translatable into observed changes in forest biomass (Wang et al., 2020). Additionally, this element cannot be straightforwardly incorporated into a mass balance approach as implemented in this study. However, we want to underline the importance of this aspect for biomass growth and argue for more in-depth analysis of future studies investigating forest biomass C dynamics.

| **a**  **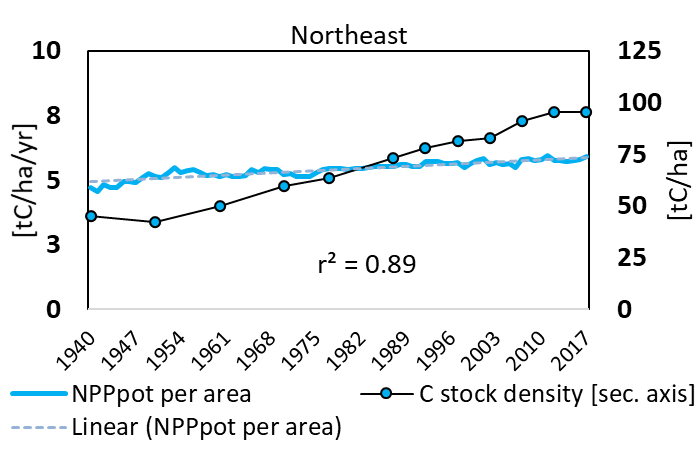** | **b**  **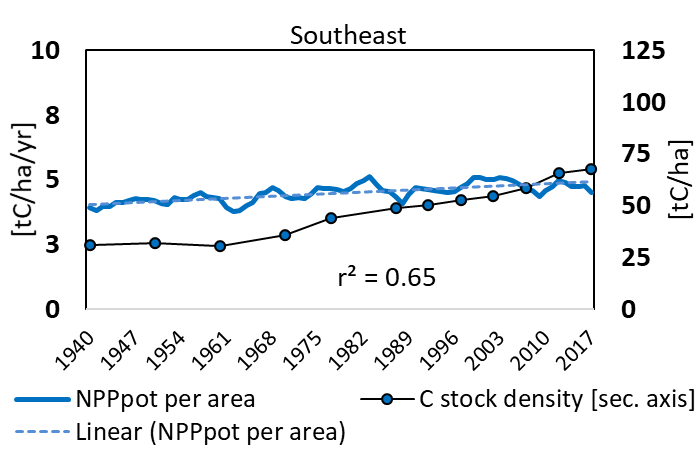** |
| --- | --- |
| **c**  **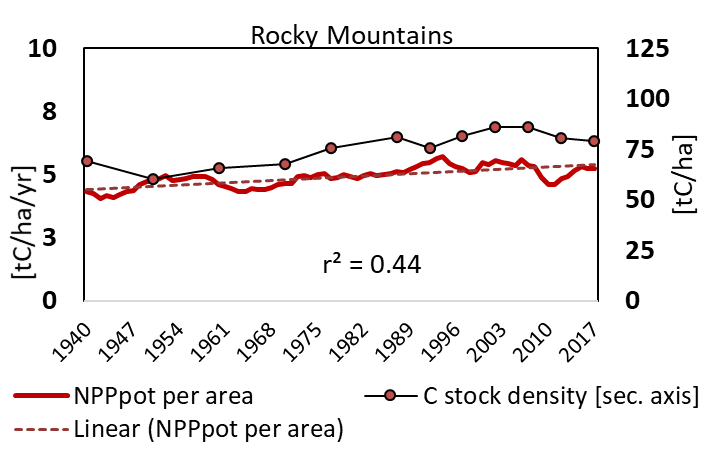** | **d**  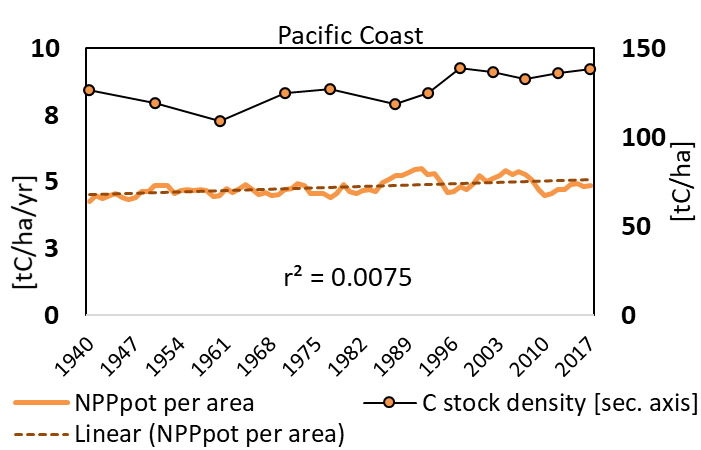 |
| **Figure S7**. Trajectories of NPP_pot_ (Potential Net Primary Production) per area and C stock densities in forests, and linear correlations between them in the four regions of the contiguous U.S. **a**) Northeast **b**) Southeast **c**) Rocky Mountains **d**) Pacific Coast. Annual data in tons C per hectare, 1910-2017. significance level for all correlations α = 0.05 | |


## Uncertainty analysis

Text S4**.** We re-estimated burned biomass using published minimum and maximum fuel load- and combustion completeness factors, both static and dynamic, to assess the sensitivity range of our results. **Table S7** displays the fuel loads, **Table S8** the combustion completeness factors used in the sensitivity analysis. We calculated 4 different variants (**Table S9**), determined by combining the different fuel loads, combustion completeness factors, and levels of aggregation of forest categories. For each of these 4 variants, we calculated 3 sub-variants (a-c), yielding in total 48 variations for biomass burned for the years 1941-2017 on the level of the four aggregated regions (Oswalt et al., 2018, Northeast, Southeast, Rocky Mountains, Pacific Coast).

Variants 1 and 2 are based on fuel-loadings modelled by LPJ-GUESS SPITFIRE. For variant 1, we used average SPITFIRE fuel loads for total burned forest area, whereas for variant 2 we attributed these fuel loads to the separate forest categories as explained in the main text, section 2.

Variants 3 and 4 were based on static fuel loads from Urbanski et al. (2018) in comparison to the dynamic fuel loads used for the main study result, in order to account for changing fuel loads due to climate variability. For variant 4 we used the static Urbanski fuel loads again but excluded 1hr and duff fuel-loadings for better comparison with the SPITFIRE derived assessment: The 1hr, litter and duff values modelled by SPITFIRE were much lower than those reported by Urbanski while the 10, 100, 1000hr deadwood and grasses/herbs fuel where fairly similar. We additionally tested the influence of different combustion completeness levels on the 4 variants, using published low (1a-4a, dotted lines), moderate (1b-4b, dashed lines), and high (1c-4c, dashed and dotted lines) severity combustion completeness factors derived from Yang et al., (2015).

|  | | **1941** | **1985** | **2017** |  |  | | **2017** |
| --- | --- | --- | --- | --- | --- | --- | --- | --- |
| **LPJGUESS-SPITFIRE, dynamic** | | | | |  | **Urbanski et al., 2018, static** | | |
|  | | | | |  | **Duff + Litter** | | |
|  |  |  |  |  |  |  | Northeast | 52.30 |
|  |  |  |  |  |  |  | Southeast | 24.25 |
|  |  |  |  |  |  |  | Rocky Mountains | 16.45 |
|  |  |  |  |  |  |  | Pacific Coast | 21.55 |
| **1hr + litter** | | | | |  | **1hr** | | |
|  | Northeast | 3.20 | 3.34 | 3.00 |  |  | Northeast | 9.45 |
|  | Southeast | 0.92 | 1.03 | 1.03 |  |  | Southeast | 26.78 |
|  | Rocky Mountains | 2.30 | 2.63 | 2.46 |  |  | Rocky Mountains | 10.35 |
|  | Pacific Coast | 1.62 | 2.02 | 1.79 |  |  | Pacific Coast | 16.78 |
| **10hr** | | | | |  | **10hr** | | |
|  | Northeast | 1.91 | 1.72 | 1.50 |  |  | Northeast | 1.08 |
|  | Southeast | 0.43 | 0.39 | 0.41 |  |  | Southeast | 1.00 |
|  | Rocky Mountains | 1.20 | 0.95 | 0.80 |  |  | Rocky Mountains | 1.10 |
|  | Pacific Coast | 0.70 | 0.67 | 0.64 |  |  | Pacific Coast | 1.23 |
| **100hr** | | | | |  | **100hr** | | |
|  | Northeast | 2.56 | 2.31 | 2.02 |  |  | Northeast | 3.60 |
|  | Southeast | 0.58 | 0.52 | 0.55 |  |  | Southeast | 3.00 |
|  | Rocky Mountains | 1.61 | 1.28 | 1.08 |  |  | Rocky Mountains | 3.70 |
|  | Pacific Coast | 0.94 | 0.89 | 0.86 |  |  | Pacific Coast | 3.85 |
| **1000hr** | | | | |  | **1000hr** | | |
|  | Northeast | 35.17 | 31.73 | 27.73 |  |  | Northeast | 7.78 |
|  | Southeast | 7.95 | 7.15 | 7.56 |  |  | Southeast | 3.33 |
|  | Rocky Mountains | 22.13 | 17.61 | 14.82 |  |  | Rocky Mountains | 26.79 |
|  | Pacific Coast | 13.00 | 12.30 | 11.84 |  |  | Pacific Coast | 15.05 |
| **Grasses/Shrubs** | | | | |  | **Grasses/Shrubs** | | |
|  | Northeast | 0.12 | 0.12 | 0.30 |  |  | Northeast | 0.30 |
|  | Southeast | 0.18 | 0.15 | 0.25 |  |  | Southeast | 0.33 |
|  | Rocky Mountains | 0.14 | 0.12 | 0.22 |  |  | Rocky Mountains | 1.39 |
|  | Pacific Coast | 0.17 | 0.09 | 0.14 |  |  | Pacific Coast | 1.58 |
| **Canopy** | | | | |  | **Canopy** | | |
|  | Northeast | 0.24 | 0.26 | 0.22 |  |  | Northeast | 5.20 |
|  | Southeast | 0.22 | 0.25 | 0.26 |  |  | Southeast | 1.60 |
|  | Rocky Mountains | 0.15 | 0.17 | 0.14 |  |  | Rocky Mountains | 9.59 |
|  | Pacific Coast | 0.12 | 0.15 | 0.12 |  |  | Pacific Coast | 4.93 |

**Table S7.** Fuel-loadings by fuel compartment and region used for sensitivity analyses. See Figure S1 for full temporal dynamics of ‘LPJGUESS-SPITFIRE, dynamic’.

| **Severity** | **Litter (<7.6 cm diameter)** | **Deadwood (>7.6 cm diameter)** | **Canopy (stem and leaf average)** | **Grasses/Shrubs (average)** |
| --- | --- | --- | --- | --- |
| Low | 0.64 | 0.35 | 0.20 | 0.41 |
| Moderate | 0.64 | 0.42 | 0.49 | 0.58 |
| High | 0.99 | 0.55 | 0.75 | 0.96 |

**Table S8**. Combustion completeness factors for average forest fuels, obtained from Yang et al., (2015), Table 1.

| **Estimation** | **Fuel loads** | **Combustion completeness** | **Forest categories** |
| --- | --- | --- | --- |
| **Study Results** | Urbanski et al. 2018, dynamic | SPITFIRE, dynamic | State & private, federal, other forest |
|  | | | |
| **Sensitivity Variants** |  | | |
| **1** | SPITFIRE, dynamic | SPITFIRE, dynamic | aggregated forest |
| 1a | SPITFIRE, dynamic | Yang et al., 2015, low severity | aggregated forest |
| 1b | SPITFIRE, dynamic | Yang et al., 2015, moderate severity | aggregated forest |
| 1c | SPITFIRE, dynamic | Yang et al., 2015, high severity | aggregated forest |
|  | | | |
| **2** | SPITFIRE, dynamic | SPITFIRE, dynamic | State & private, federal, other forest |
| 2a | SPITFIRE, dynamic | Yang et al., 2015, low severity | aggregated forest |
| 2b | SPITFIRE, dynamic | Yang et al., 2015, moderate severity | aggregated forest |
| 2c | SPITFIRE, dynamic | Yang et al., 2015, high severity | aggregated forest |
|  | | | |
| **3** | Urbanski et al. 2018, static | SPITFIRE, dynamic | State & private, federal, other forest |
| 3a | Urbanski et al. 2018, static | Yang et al., 2015, low severity | aggregated |
| 3b | Urbanski et al. 2018, static | Yang et al., 2015, moderate severity | aggregated |
| 3c | Urbanski et al. 2018, static | Yang et al., 2015, high severity | aggregated |
|  | | | |
| **4** | Urbanski et al. 2018, static, excluding duff | SPITFIRE, dynamic | State & private, federal, other forest |
| 4a | Urbanski et al. 2018, static, excluding duff | Yang et al., 2015, low severity | aggregated forest |
| 4b | Urbanski et al. 2018, static, excluding duff | Yang et al., 2015, moderate severity | aggregated forest |
| 4c | Urbanski et al. 2018, static, excluding duff | Yang et al., 2015, high severity | aggregated forest |

**Table S9.** Overview table of fuel loads, combustion completeness factors, and forest categories used for the sensitivity analyses (Figure S 7)

| **a**  **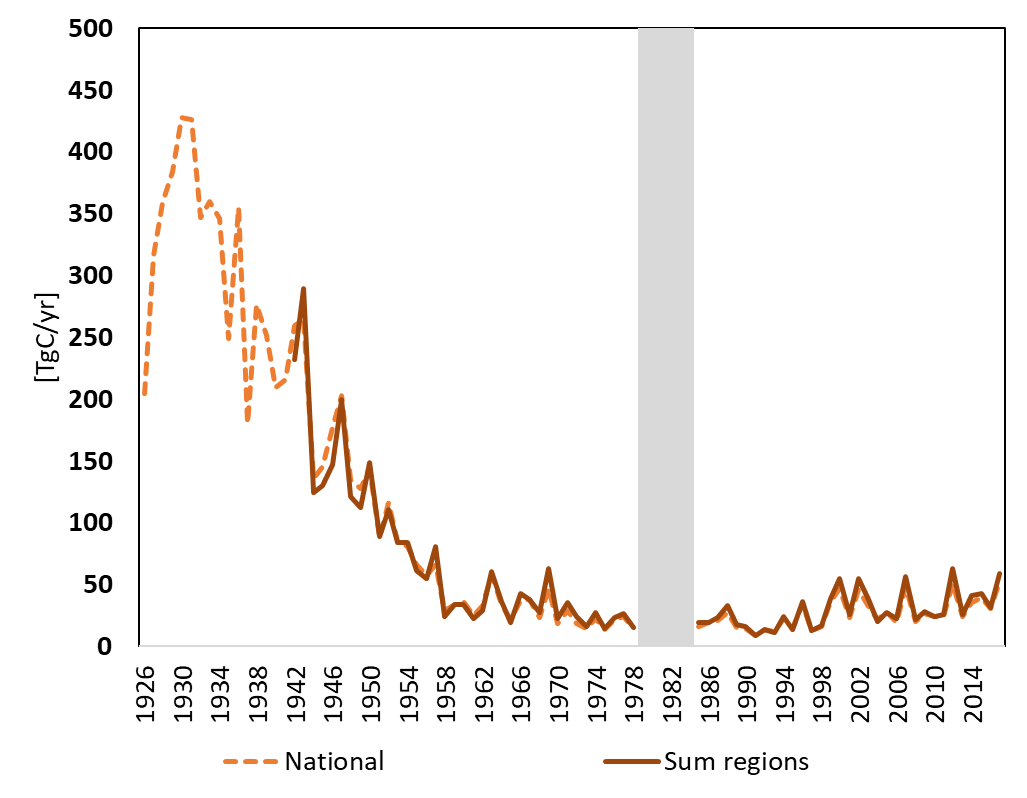** | **b**  **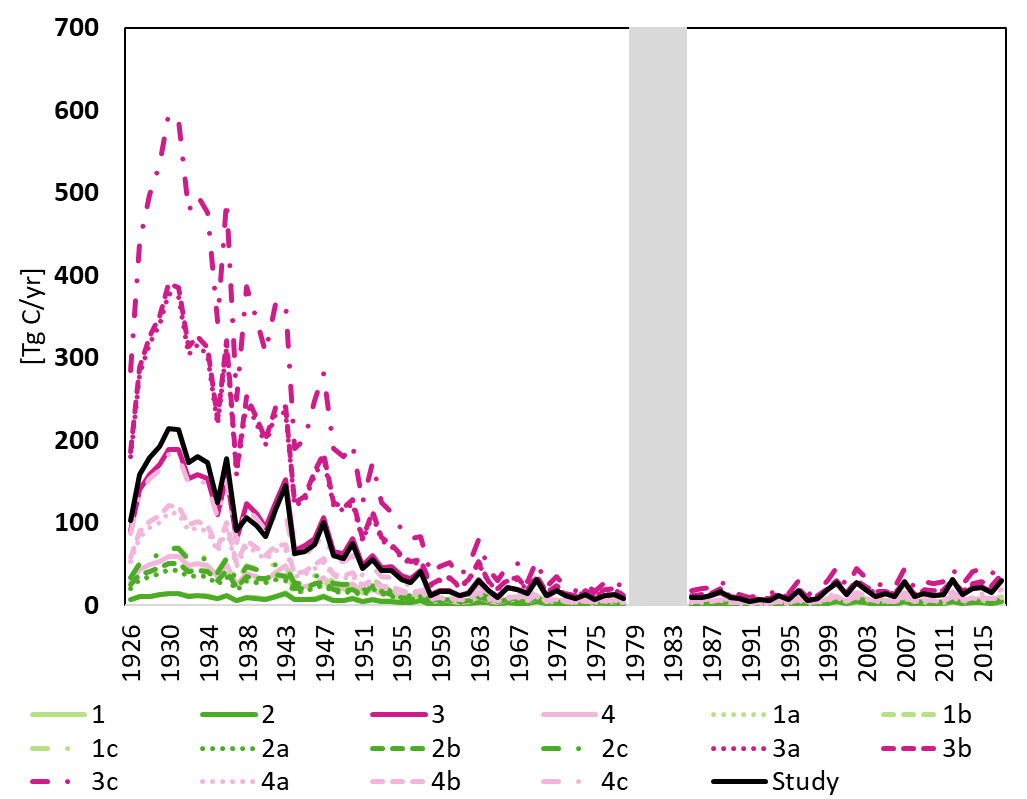** |
| --- | --- |
| **c**  **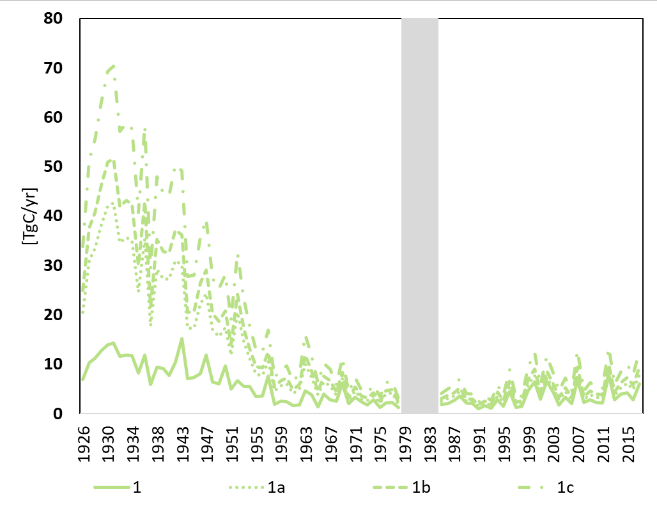** | **d**  **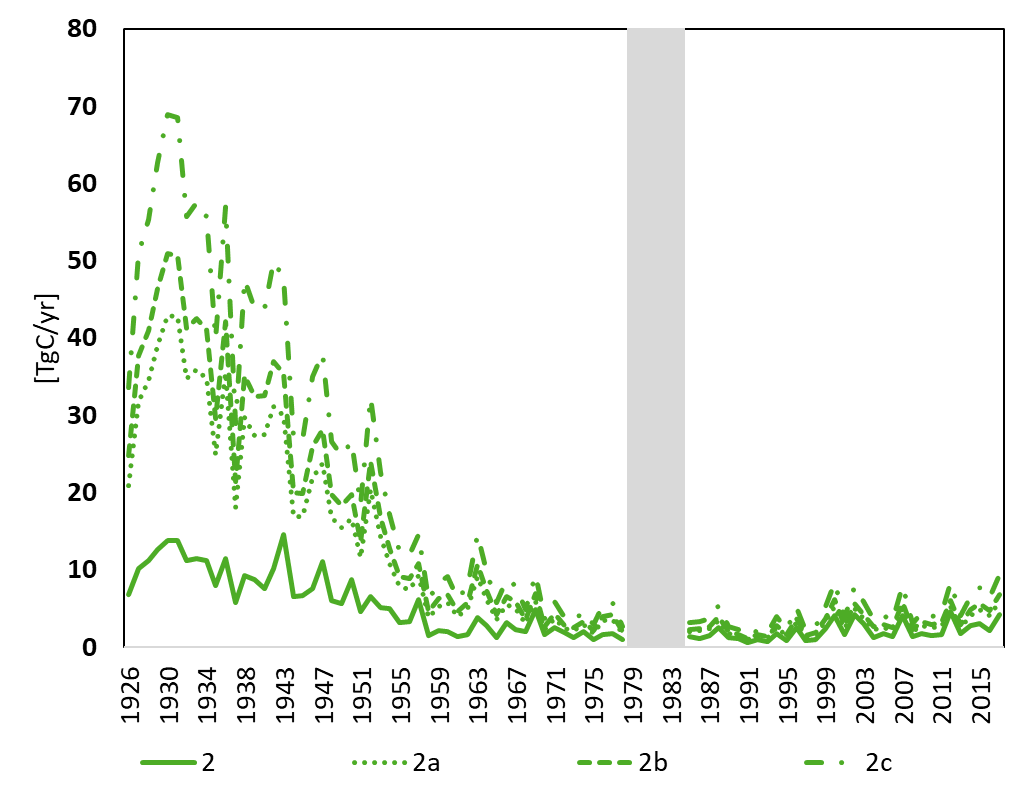** |
| **e**  **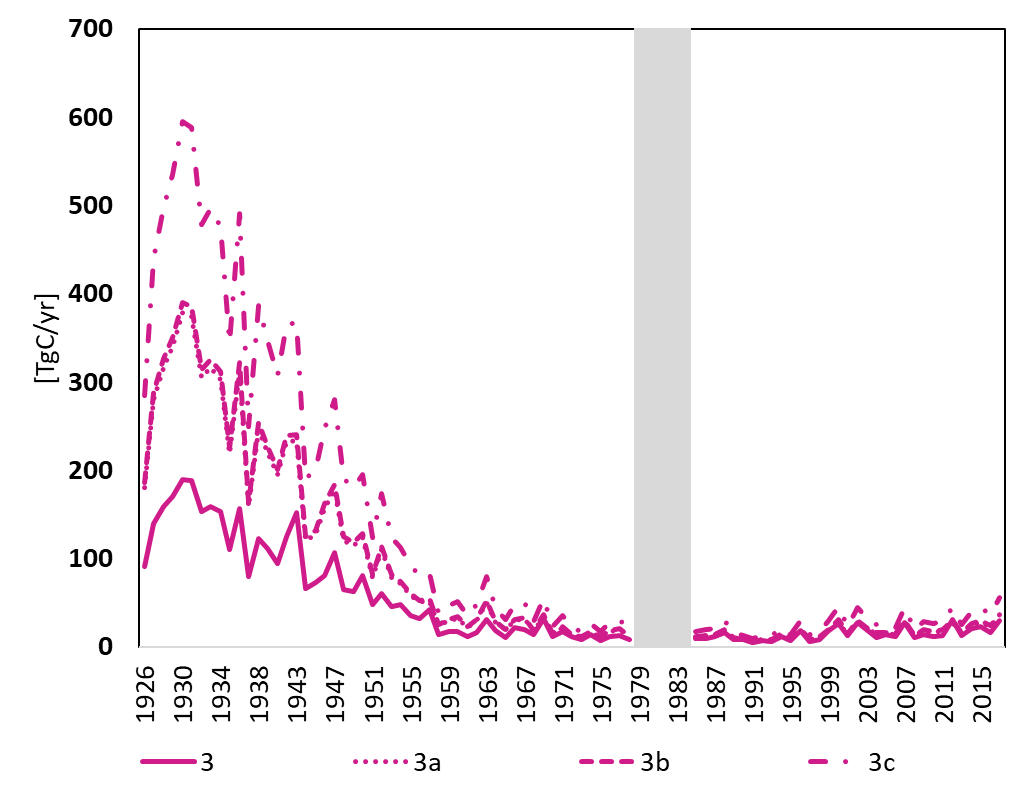** | **f**  **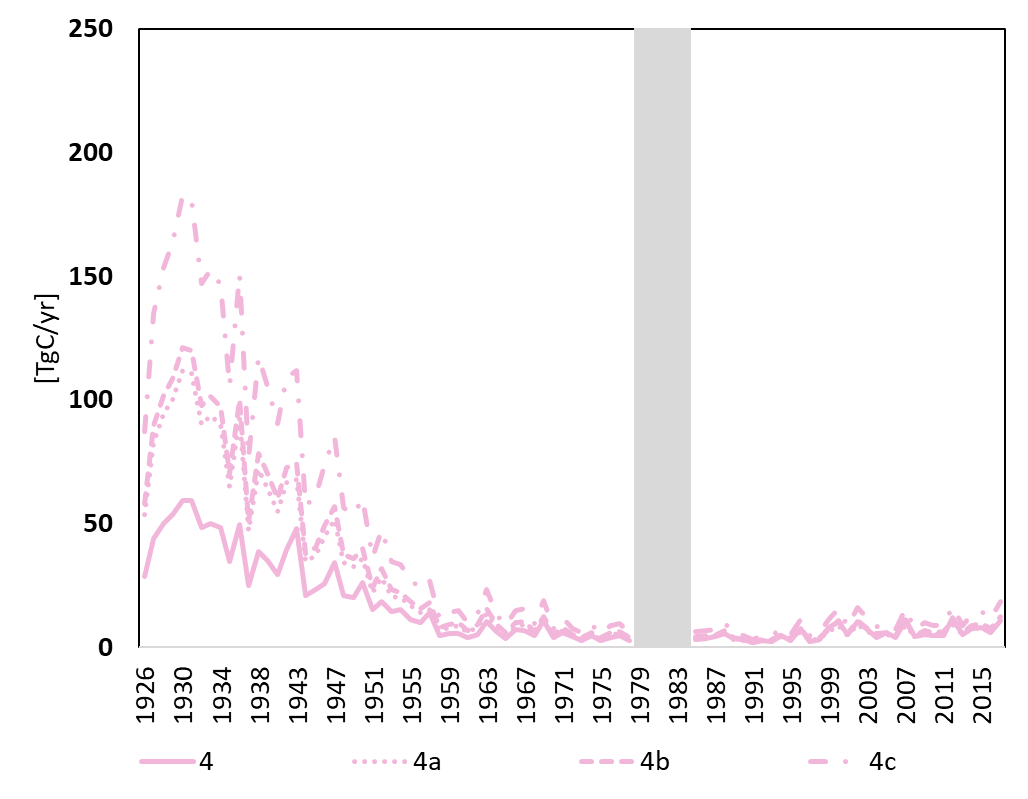** |
| **Figure S8.** Scaling effects and aggregated sensitivity analysis estimation results for burned biomass **a)** Scaling effect assessment of national total (based on average fuel loads and combustion completeness) vs sum of regional estimates (based on region specific factors) **b)** All 16 sensitivity variants of the sensitivity analysis and study result **c-f)** Main sensitivity variants 1-4 and sub-variants (1-4a,b,c). Variant 1 and 2 are estimated using SPITFIRE average dynamic fuel loadings. Variant 1 uses total aggregated burned area (figure **b**, **c**, solid lines). Variant 2 uses burned area for separate forest categories (Figure b, d, solid lines). Variants 3 and 4 (Figure e, f, solid lines) are based on static Urbanski et al. 2018 fuel loadings, Variant 4 excludes duff full loadings. Sub-variants for main variants 1-4 used static combustion completeness factors from Yang et al., 2015 for low (b-f, dotted lines), moderate (b-f, dashed lines), and high (b-f, dashed and dotted lines) severity. See text for details. Note the different y axis. | |

Text S5. To test for possible scaling effects, we compared burned biomass estimated using the national aggregated total burned area and average fuel-loadings and combustion completeness factors (**Figure S8**a, dashed line) for 1926-2017 with our regional study estimates using region specific fuel-loadings and combustion completeness factors (**Figure S8**a, solid line) for the period 1941-2017. On average, for the overlapping period, the two estimations agree 99% (for single years, the largest negative deviation was 20%, the largest positive deviation 26%). We conclude that overall, no relevant scaling effects exist and that the estimation method for the period 1926-1941 produces plausible results.

The sensitivity analysis (**Figure S8**b-f) shows a maximum deviation of burned biomass by +223 (4x larger, variant 3c) and -75 (11x smaller, variant 2) TgC from the best guess study result. Using different combustion completeness factors and fuel loadings had the largest impact on burned biomass, while the effect of disaggregating burned area into different forest categories was less pronounced. Results vary only slightly between variants 1, 2, and 4. The combination of dynamic SPITFIRE combustion completeness factors and inclusion of duff fuel loads by Urbanski et al (2018) (variant 3) resulted in 11-12 times higher burned biomass compared to the other three estimates. The use of the published low-to-high combustion completeness factors (1a-c, 2a-c, 3a-c, 4a-c) yielded 2-11x higher numbers compared to using LPJ-GUESS SPITFIRE modelled combustion completeness (1-4). Using static instead of dynamic fuel loadings (Variant 3) had little impact on the results, showing an average 3% higher biomass burned (maximum + 14%, minimum -6%) over the entire time in comparison to our main result. The estimated sensitivity range is quite large, but the maximum and minimum variants represent rather extreme scenarios. For example, variant 3c assumes that all fires burned with high severity, meaning that 99% of grasses, small litter and 1hr deadwood, 55% of 10-1000hr deadwood, and 75% of canopy fuels would burn in each fire event.

| **Density** | **Bark factor** | **Recovery rate** | **Belowground (root-to-shoot) ratio** |
| --- | --- | --- | --- |
| **Industrial wood** | | | |
| **Minimum** | | | |
| Softwood | | | |
| 0.41 | 0.78 | 0.46 | 0.78 |
| Hardwoods | | | |
| 0.58 | 0.75 | 0.46 | 0.78 |
| **Maximum** | | | |
| Softwood | | | |
| 0.43 | 0.98 | 0.98 | 0.79 |
| Hardwoods | | | |
| 0.60 | 0.99 | 1.0 | 0.79 |
| **Fuelwood** | | | |
| **Minimum** | | | |
| Softwood | | | |
| 0.41 | 0.80 | 0.74 | 0.78 |
| Hardwoods | | | |
| 0.58 | 0.75 | 0.69 | 0.78 |
| **Maximum** | | | |
| Softwood | | | |
| 0.43 | 0.98 | 0.90 | 0.79 |
| Hardwoods | | | |
| 0.60 | 0.99 | 1.00 | 0.79 |

**Table S10.** Overview table of wood density and harvest losses expansion factors used for the sensitivity analyses of wood harvest (Figure S8)

To assess the sensitivity of our wood harvest reconstruction, we calculated minimum and maximum variants for industrial wood and fuelwood for each wood type (hardwood/softwood). We used minimum and maximum wood density and expansion factors from Kastner et al. (2021, **Table S10**) to estimate a range for possible deviations of total above and belowground harvest losses from our study result.

As we did for the biomass burned, we estimated the sensitivity from 1926-1941 on the national scale and additionally from 1941-2017 on the regional scale (**Figure S9**). Not surprisingly, the largest deviations are found for the Southeast, as total harvest rates in this region are the highest across the contiguous U.S. and thus, applying different expansion factors caused the largest deviations. Overall, the average deviation from our study result was -34% and +64% over the period 1926-2017, with a largest minimum deviation from our study result of -47% or 80 TgC in 1926 and maximum deviation of +96% or 149 TgC in 1970.

The 2017 USFS forest inventory also provides data for harvest losses, which lie within our range of variation. Our best guess estimate is on average 24% higher than the USFS estimate, probably because the harvest expansion factors used additionally estimate the total above and below biomass killed during harvest (including leaves and roots). In contrast, the USFS define logging residues as “The unused portions of growing-stock trees cut or killed by logging and left in the woods” (Oswalt et al., 2018). Although this includes “limbs, treetops and stumps”, it is not clear whether total foliage and roots of felled trees are included as well. In this regard, while our best-guess estimate may be considered more progressive than the USFS estimate, by using the same factors for the whole time-period, we ensure consistency across time.

| **a**  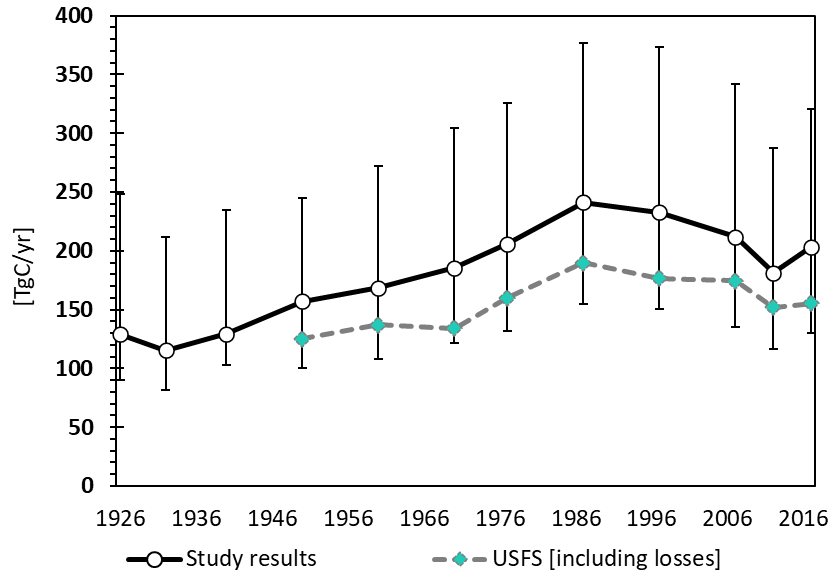 | |
| --- | --- |
| **b**  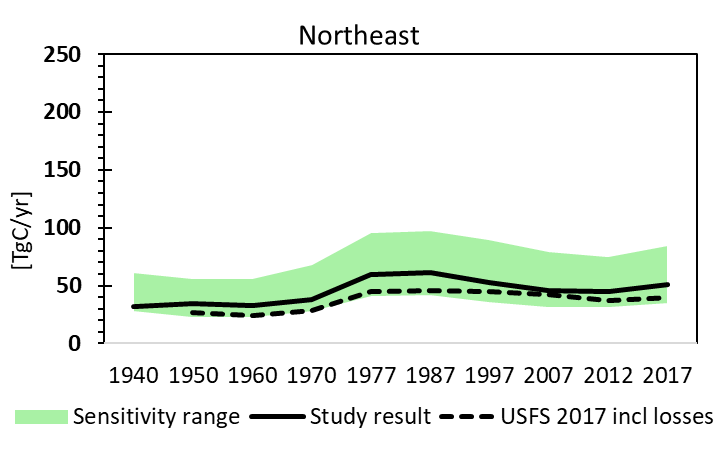 | **c**  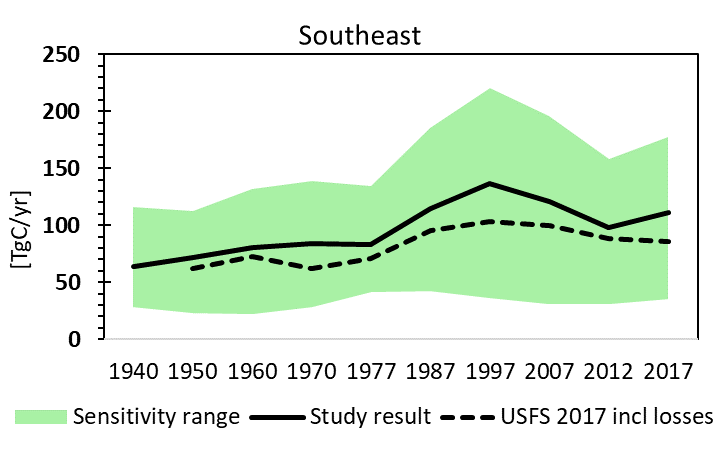 |
| **d**  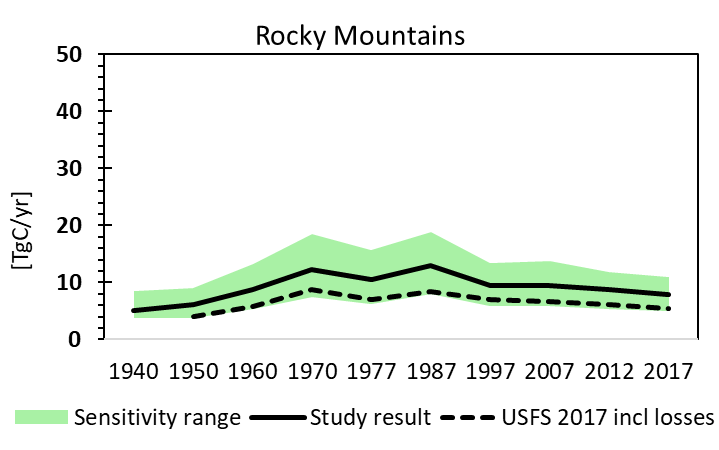 | **e**  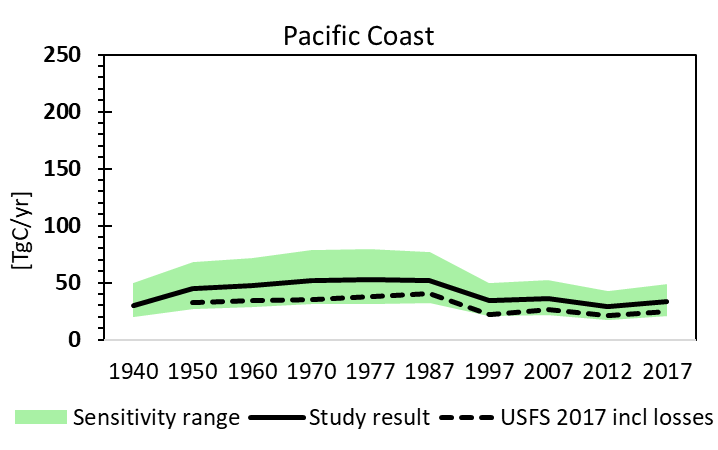 |

**Figure S9.** Sensitivity ranges for total wood harvest reconstruction (industrial wood + fuelwood), including losses vs USFS 2017 harvest estimate including losses for the contiguous U.S. **a**) National total 1926-2017 **b**) Northeast 1941-2017 **c**) Southeast 1941-2017 **d**) Rocky Mountains 1941-2017 **e**) Pacific Coast 1941-2017. Notes: Solid lines represent the reconstruction used in this study. Dashed lines represent USFS 2017 harvest data including losses. Whiskers and green areas represent sensitivity ranges.

## Other Landsat burned area

| **a**  **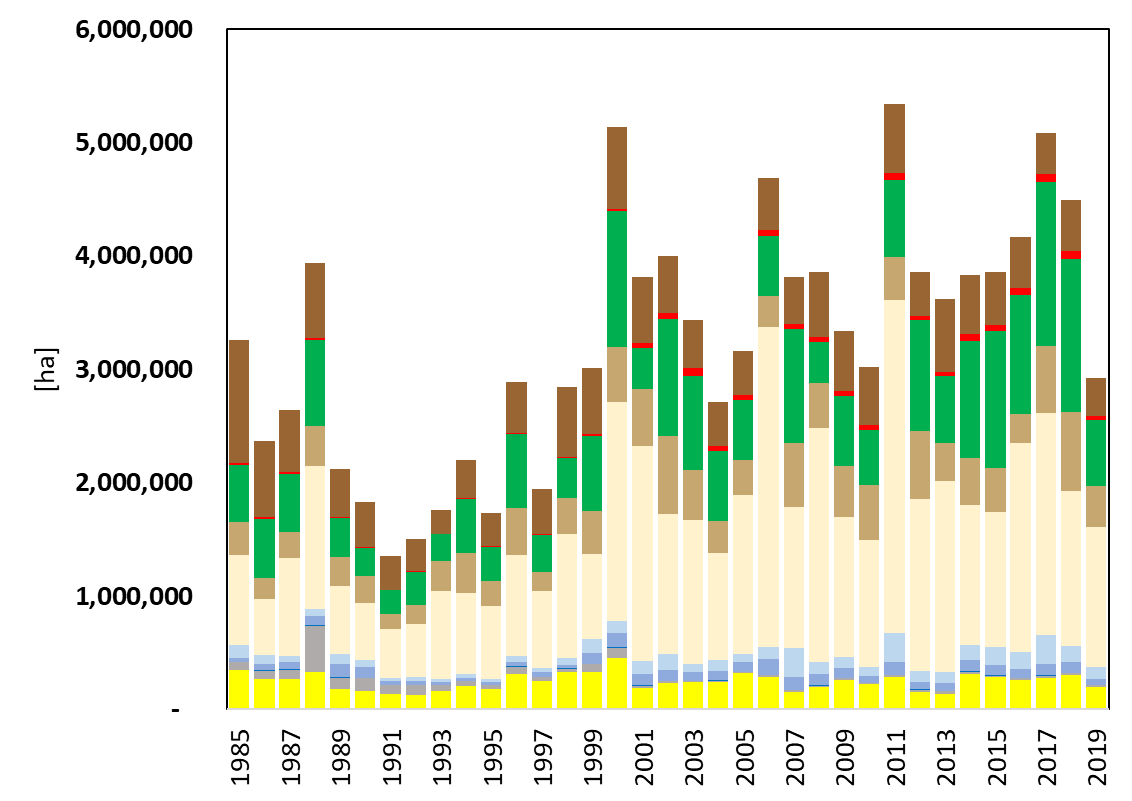** | **b**  **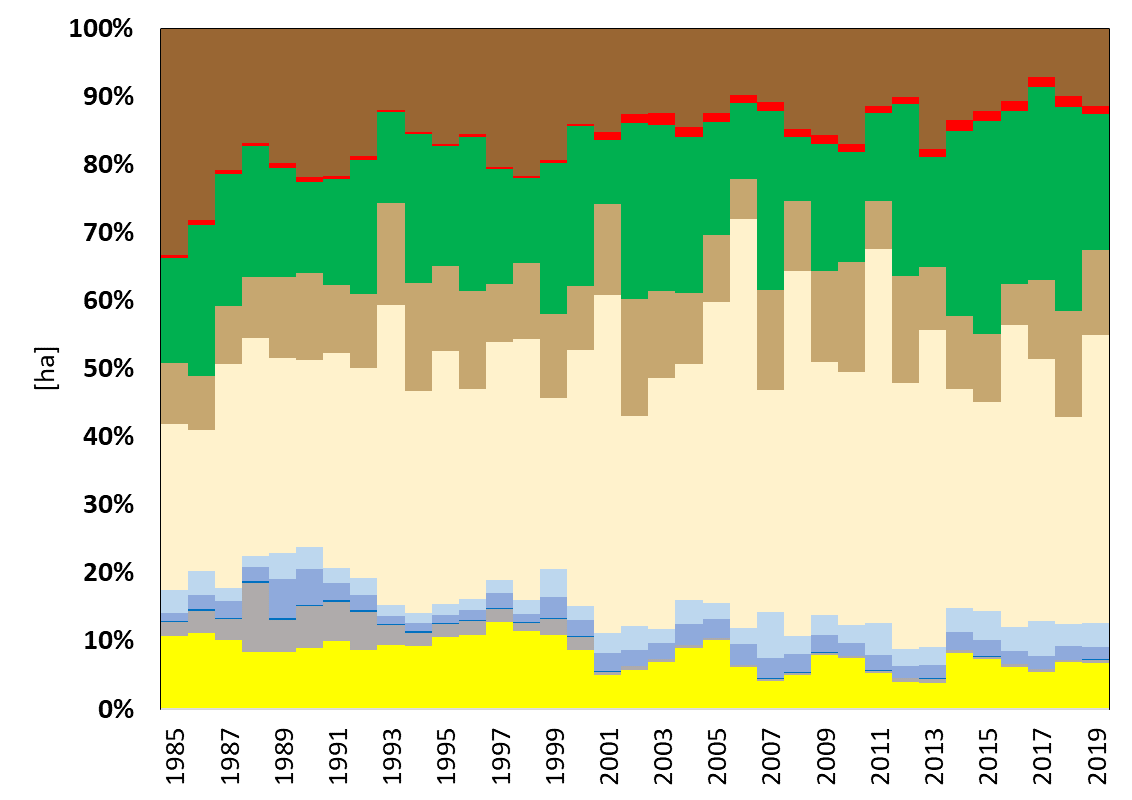** |
| --- | --- |
| **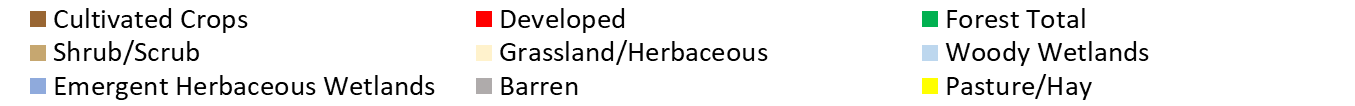** | |
| **Figure S10.** Total burned area by NLCD Land cover type for the contiguous U.S., as reported by Landsat burned area algorithm (Hawbaker et al., 2020) **a**) absolute values, [ha/yr] **b**) relative values, shares of total | |

## References

FAO. (2020). *FAOSTAT Statistical Database*. http://www.fao.org/faostat/en/#home

Fedkiw, J. (1989). *The Evolving Use and Management of the Nation’s Forests, Grasslands, Croplands, and Related Resources. A Technical Document Supporting the 1989 USDA Forest Service RPA Assessment* (Vol. 175). US Department of Agriculture, Forest Service, Rocky Mountain Forest and Range Experiment Station.

Gierlinger, S., & Krausmann, F. (2012). The Physical Economy of the United States of America: Extraction, Trade, and Consumption of Materials from 1870 to 2005. *Journal of Industrial Ecology*, *16*(3), 365–377. https://doi.org/10.1111/j.1530-9290.2011.00404.x

Haberl, H., Erb, K.-H., & Krausmann, F. (2014). Human Appropriation of Net Primary Production: Patterns, Trends, and Planetary Boundaries. *Annual Review of Environment and Resources*, *39*(1), 363–391. https://doi.org/10.1146/annurev-environ-121912-094620

Haverd, V., Smith, B., Canadell, J. G., Cuntz, M., Mikaloff‐Fletcher, S., Farquhar, G., Woodgate, W., Briggs, P. R., & Trudinger, C. M. (2020). Higher than expected CO _2_ fertilization inferred from leaf to global observations. *Global Change Biology*, *26*(4), 2390–2402. https://doi.org/10.1111/gcb.14950

Hawbaker, T. J., Vanderhoof, M. K., Schmidt, G. L., Beal, Y.-J., Picotte, J. J., Takacs, J. D., Falgout, J. T., & Dwyer, J. L. (2020). The Landsat Burned Area algorithm and products for the conterminous United States. *Remote Sensing of Environment*, *244*, 111801. https://doi.org/10.1016/j.rse.2020.111801

Howard, J. L., & Liang, S. (2019). US timber production, trade, consumption, and price statistics, 1965-2017. *Res. Pap. FPL-RP-701. Madison, WI: US Department of Agriculture, Forest Service, Forest Products Laboratory. 96 p.*, *701*, 1–96.

IEA. (2015). *Extended world energy balances*. OECD Publishing. http://www.oecd-ilibrary.org/energy/data/iea-world-energy-statistics-and-balances/extended-world-energy-balances_data-00513-en

Ince, P. J. (2000). *Industrial wood productivity in the United States, 1900-1998* (Vol. 272). US Department of Agriculture, Forest Service, Forest Products Laboratory.

Kastner, T., Chaudhary, A., Gingrich, S., Marques, A., Persson, U. M., Bidoglio, G., Le Provost, G., & Schwarzmüller, F. (2021). Global agricultural trade and land system sustainability: Implications for ecosystem carbon storage, biodiversity, and human nutrition. *One Earth*. https://doi.org/10.1016/j.oneear.2021.09.006

Kastner, T., Matej, S., Forrest, M., Gingrich, S., Haberl, H., Hickler, T., Krausmann, F., Lasslop, G., Niedertscheider, M., Plutzar, C., Schwarzmüller, F., Steinkamp, J., & Erb, K. (2022). Land use intensification increasingly drives the spatiotemporal patterns of the global human appropriation of net primary production in the last century. *Global Change Biology*, *28*(1), 307–322. https://doi.org/10.1111/gcb.15932

Krausmann, F., Erb, K.-H., Gingrich, S., Haberl, H., Bondeau, A., Gaube, V., Lauk, C., Plutzar, C., & Searchinger, T. D. (2013). Global human appropriation of net primary production doubled in the 20th century. *Proceedings of the National Academy of Sciences*, *110*(25), 10324–10329. https://doi.org/10.1073/pnas.1211349110

Manthy, R. S., & Potter, N. (1978). *Natural resource commodities: A century of statistics: Prices, output, consumption, foreign trade, and employment in the United States, 1870-1973*. Published for Resources for the Future by the Johns Hopkins University Press.

Oswalt, S. N., Miles, P. D., Pugh, S. A., & Smith, W. Brad. (2018). *Forest Resources of the United States, 2017: A technical document supporting the Forest Service 2020 update of the RPA Assessment* (Resources Planning Act (RPA) Assessment, p. 146) [Forest resource statistics]. U.S. Department of Agriculture, Forest Service.

Reynolds, R. V. R., & Pierson, A. H. (1942). *Fuel wood used in the United States, 1630-1930* (Issue 641). US Department of Agriculture.

Ruefenacht, B., Finco, M. V., Nelson, M. D., Czaplewski, R., Helmer, E. H., Blackard, J. A., Holden, G. R., Lister, A. J., Salajanu, D., Weyermann, D., & Winterberger, K. (2008). Conterminous U.S. and Alaska Forest Type Mapping Using Forest Inventory and Analysis Data. *Photogrammetric Engineering & Remote Sensing*, *74*(11), 1379–1388. https://doi.org/10.14358/PERS.74.11.1379

Schurr, S. H., & Netschert, B. C. (1960). *Energy in the American economy, 1850-1975, an economic study of its history and prospects*. Johns Hopkins Press.

United Nations Statistics Division. (2020). *UN fuelwood database*. https://data.un.org/Data.aspx?d=EDATA&f=cmID%3AFW%3BtrID%3A1231

United States Bureau of the Census. (1975). *Historical statistics of the United States, colonial times to 1970*. US Department of Commerce, Bureau of the Census.

United States Forest Service. (1958). *Forest Fire Statistics 1958* (Forest Fire Statistics, p. 14) [Statistical Report]. United States Department of Agriculture; University of Florida.

Urbanski, S. P., Reeves, M. C., Corley, R. E., Silverstein, R. P., & Hao, W. M. (2018). Contiguous United States wildland fire emission estimates during 2003–2015. *Earth System Science Data*, *10*(4), 2241–2274. https://doi.org/10.5194/essd-10-2241-2018

U.S. Bureau of the Census. (1984). *Statistical Abstract of the United States: 1985* (No. 105). Government Printing Office. https://www2.census.gov/library/publications/1984/compendia/statab/105ed/1985-01.pdf

U.S. Department of Commerce and Labor. (1908). *Statistical Abstract of the United States: 1907*. Government Printing Office. https://www.census.gov/library/publications/1908/compendia/statab/30ed.html

U.S. Department of Commerce and Labor. (1921). *Statistical Abstract of the United States: 1920* (No. 43). Government Printing Office. https://www.census.gov/library/publications.html

U.S. Department of Commerce and Labor. (1930). *Statistical Abstract of the United States: 1930* (No. 52). Government Printing Office. https://www2.census.gov/library/publications/1930/compendia/statab/52ed/1930-01.pdf

U.S. Energy Information Administration. (2020). *Total End-Use Energy consumption 1960-2017 United States*.

U.S. Geological Survey. (2018). *USGS Commodity Statistics and Information*. U.S. Department of the Interior. https://minerals.usgs.gov/minerals/pubs/commodity/

USDA Forest Service. (1932). *Forest Situation in the United States* (p. 101) [Special Report]. United States Department of Agriculture.

USDA Forest Service. (1974). *The Outlook for Timber in the United States* (General Technical Report No. 20; Forest Resource Report, p. 390). U.S. Department of Agriculture, Forest Service. https://www.fs.fed.us/research/docs/rpa/pre-1989/1974%20US%20Timber%20Outlook.pdf

Walker, A. P., De Kauwe, M. G., Bastos, A., Belmecheri, S., Georgiou, K., Keeling, R. F., McMahon, S. M., Medlyn, B. E., Moore, D. J. P., Norby, R. J., Zaehle, S., Anderson‐Teixeira, K. J., Battipaglia, G., Brienen, R. J. W., Cabugao, K. G., Cailleret, M., Campbell, E., Canadell, J. G., Ciais, P., … Zuidema, P. A. (2021). Integrating the evidence for a terrestrial carbon sink caused by increasing atmospheric CO _2_. *New Phytologist*, *229*(5), 2413–2445. https://doi.org/10.1111/nph.16866

Wang, S., Zhang, Y., Ju, W., Chen, J. M., Ciais, P., Cescatti, A., Sardans, J., Janssens, I. A., Wu, M., Berry, J. A., Campbell, E., Fernández-Martínez, M., Alkama, R., Sitch, S., Friedlingstein, P., Smith, W. K., Yuan, W., He, W., Lombardozzi, D., … Peñuelas, J. (2020). Recent global decline of CO2 fertilization effects on vegetation photosynthesis. *Science*, *370*(6522), 1295–1300. https://doi.org/10.1126/science.abb7772

Warde, P. (2019). Firewood consumption and energy transition: A survey of sources, methods and explanations in Europe and North America. *Historia Agraria: Revista de Agricultura e Historia Rural*, *77*, 7–32. https://doi.org/10.26882/histagrar.077e02w

Yang, J., Tian, H., Tao, B., Ren, W., Pan, S., Liu, Y., & Wang, Y. (2015). A growing importance of large fires in conterminous United States during 1984–2012. *Journal of Geophysical Research: Biogeosciences*, *120*(12), 2625–2640. https://doi.org/10.1002/2015JG002965
